# Unique Phenanthrenes from *Juncus ensifolius* and Their Antiproliferative and Synergistic Effects with the Conventional Anticancer Agent Doxorubicin against Human Cancer Cell Lines

**DOI:** 10.3390/pharmaceutics14030608

**Published:** 2022-03-10

**Authors:** Dóra Stefkó, Norbert Kúsz, Nikoletta Szemerédi, Anita Barta, Gabriella Spengler, Róbert Berkecz, Judit Hohmann, Andrea Vasas

**Affiliations:** 1 Department of Pharmacognosy, Interdisciplinary Excellence Centre, University of Szeged, 6720 Szeged, Hungary; stefko.dori@gmail.com (D.S.); kusznorbert@gmail.com (N.K.); bartaanita96@gmail.com (A.B.); hohmann.judit@szte.hu (J.H.); 2 Department of Medical Microbiology, Albert Szent-Györgyi Health Center, Albert Szent-Györgyi Medical School, University of Szeged, 6725 Szeged, Hungary; szemeredi.nikoletta@med.u-szeged.hu (N.S.); spengler.gabriella@med.u-szeged.hu (G.S.); 3 Institute of Pharmaceutical Analysis, University of Szeged, 6720 Szeged, Hungary; berkecz.robert@szte.hu; 4 Interdisciplinary Centre of Natural Products, University of Szeged, 6720 Szeged, Hungary

**Keywords:** *Juncus ensifolius*, Juncaceae, phenanthrene, antiproliferative, doxorubicin, combination assay, synergism

## Abstract

Phenanthrenes are the main special metabolites of Juncaceae species from phytochemical, pharmacological, and chemotaxonomical points of view. The present study focused on the isolation, structure determination, and pharmacological investigation of phenanthrenes from *Juncus ensifolius*. Nineteen compounds, including 17 phenanthrenes, were identified from the methanol extract of the plant. Thirteen compounds, namely, ensifolins A–M (**1**–**13**), were obtained for the first time from natural sources. Four phenanthrenes [2-hydroxy-1,7-dimethyl-5-vinyl-9,10-dihydrophenanthrene (**14**), juncuenin B (**15**), juncatrin B (**16**), and sylvaticin A (**17**)], 4-hydroxybenzaldehyde (**18**) and luteolin (**19**) were isolated for the first time from *J. ensifolius*. Ensifolins A (**1**) and B (**2**) are structurally unique phenanthrenes, considering that they are flavonoid- (**1**) or benzaldehyde-adducts (**2**). The antiproliferative activity of all isolated compounds against HeLa, COLO 205, and COLO 320 cancer cells and a non-tumor (MRC-5) cell line was tested using the 3-(4,5-dimethylthiazol-2-yl)-2,5-diphenyl-2H-tetrazolium bromide (MTT) viability assay. The luteolin-substituted phenanthrene ensifolin A (**1**) proved to be the most active against all three cancer cell lines (IC_50_ values 3.9–12.7 μM) and showed good selectivity (SI = 4.95) in the case of COLO 205. The best selectivity was recorded for ensifolins D (**4**, SI > 5.15, HeLa), H (**8**, SI > 8.13, HeLa), and **17** (SI > 9.43, HeLa). The synergistic activity of the compounds with doxorubicin was also tested on HeLa cells, and ensifolins E (**5**) and H (**8**) exhibited very strong synergism (CI < 0.1). In conclusion, these phenanthrenes are worthy of further investigation.

## 1. Introduction

Cancer is one of the leading causes of death globally, and the development of new anticancer agents is the focus of research worldwide. Natural products are still the best options for finding novel agents/active templates and offer the potential to discover novel structures that can lead to effective agents for a variety of human diseases [1]. Novel biomolecules have an advantage in terms of biosafety and they can serve as leads for synthetic chemists and pharmacologists. The effective anticancer drugs often work by inhibiting angiogenesis, inducing apoptosis, and blocking cancer cells from proliferating. A common feature of phytochemicals is attenuating cancer progression by inhibition of inflammation and induction of apoptosis through caspase-dependent mechanisms or induction of intracellular oxidative stress. Several molecular targets and the action mechanisms of these molecules have already been explored, and great efforts are performed regarding their efficiency by using structure-based drug-design strategies. Ligand-based drug design is used when the target is unknown in order to identify the features of potential receptors. The molecular docking of natural compounds with the receptor targets followed by ADMET (absorption, distribution, metabolism, excretion, toxicity) analysis could help to increase the hit probability of effective drugs [2]. Moreover, natural compounds can target multiple key regulators, e.g., safranal, a metabolite of *Crocus sativus*, in the case where tumor angiogenesis significantly affects the strong interplay of hepatocellular carcinoma cells, endothelial cells, and multiple signaling molecules involved in tumor angiogenesis by downregulation of the in vitro expression of HIF-1α, VEGF, VEGFR2, p-AKT, p-ERK1/2, MMP9, p-FAK, and p-STAT3 [3]. Ginger and its active ingredients (e.g., gingerols and shogaols) could protect rat liver from cancer via synergistic multi targeted effects including antioxidants and anti-inflammatory by down regulating NF-κB. This effect is related to promoting apoptosis, inhibiting the proliferation of cells, preventing oxidative stress, and reducing COX-2, iNOS, and NF-κB p65 expressions [4].

Conventional chemotherapy plays an important role in the treatment of cancers, but clinical limitations exist because of dose-limiting side effects and drug resistance. Therefore, combination treatment of chemotherapeutic agents and natural compounds is considered to be a promising therapeutic strategy with a higher clinical efficacy. Doxorubicin is routinely used as a single drug for the treatment of patients with different types of cancer. It intercalates into DNA, stabilizes the topoisomerase II protein, and causes cell death via inhibition of topoisomerase II and the generation of reactive oxygen species and free radicals by redox reactions [5]. Although doxorubicin is an effective antineoplastic agent and has cytotoxic effects, resistance limits its use in chemotherapy [6]. A growing body of combination treatments with natural products has been reported to synergistically prevent tumor growth [5]. Besides combination with standard drugs, the efficacy and bioavailability of natural compounds can further increase by applying different formulation techniques. Recent advances in drug delivery systems describe the use of nanoemulsions, nanoparticles, liposomes, and films to carry various phytochemicals such as berberine, curcumin, resveratrol, camptothecins, and celastrol, showing a promising improved anticancer action [7,8].

A promising group of natural small molecules are phenanthrenes. The occurrence of these compounds in nature is limited to only a few plant families. Among them, Orchidaceae and Juncaceae are the most abundant sources of these specific metabolites. Phenanthrenes have chemotaxonomical significance since the presence of certain substituents in them are apparently restricted to certain families; e.g., almost all of the stilbene- and *p*-hydroxybenzyl-substituted compounds have been reported in Orchidaceae species, while vinyl substitution occurs only in Juncaceae phenanthrenes [9,10]. Moreover, phenanthrenes possess noteworthy pharmacological activities, such as antiproliferative, anti-inflammatory, and antimicrobial properties [11]. Among Juncaceae phenanthrenes, dehydroeffusol, juncusol, and juncuenin B seem to be the most promising. All of them showed a noteworthy antiproliferative effect against different human cancer cell lines. Dehydroeffusol dose dependently (12–48 µM) inhibited gastric cancer cell-mediated vasculogenic mimicry in SGC-7901 cells. It also decreased VE-cadherin expression and exposure, suppressed the MMP2 protease expression and activity, and inhibited gastric cancer cell adhesion, migration, and invasion [12]. Moreover, it inhibited the gastric cell growth and the tumorigenicity by inducing tumor-suppressive ER stress responses [13]. The flow cytometric cell-cycle analysis of juncusol showed that juncusol treatment of HeLa cells for 24 h increased the cell population in the G2/M and sub-G1 phases. It also showed pro-apoptotic properties through the presence of active caspase-3, 8, and 9 in HeLa cells, suggesting that juncusol causes cell death by apoptosis induction and inhibition of tubulin polymerization in vitro [14]. Juncuenin possessed promising antiproliferative activity (IC_50_ 2.9 µM) against HeLa cells. One of its semisynthetic derivatives, differing only in the presence of a methoxy group at C-8a and a carbonyl group at ring C, showed an even higher inhibitory effect (IC_50_ 0.9 µM) [15]. In a superoxide anion generation assay, remarkable anti-inflammatory activity was determined for juncusol (IC_50_ 3.1 µM) and juncuenin B (IC_50_ 4.9 µM). The latter also inhibited elastase release in human neutrophils (IC_50_ of 5.5 µM) in response to fMLP/CB activation [16].

In continuation of our work aiming at the isolation of biologically active compounds from Juncaceae species, *Juncus ensifolius* Wikstr. was investigated. *J. ensifolius* (swordleaf rush) is a ruderal species of rush that occurs from near sea level to subalpine elevations throughout western North America and East Asia [17]. In Europe, Australia, New Zealand, and Hawaii, *J. ensifolius* became naturalized in the 20th century. It is used as an attractive plant in garden ponds [17]. Historically, the plant was used by indigenous people in western North America for weaving mats and baskets and as food, fodder, and medicine [18,19]. The phytochemistry and pharmacology of this plant have not been previously investigated. In this work, we report on the isolation, structure determination, and antiproliferative investigation of phenanthrenes from swordleaf rush, as well as evaluation of its synergistic effects with the conventional anticancer agent doxorubicin.

## 2. Materials and Methods

### 2.1. General Procedures

Optical rotations were measured using a JASCO P-2000 polarimeter (JASCO, Tokyo, Japan). Vacuum liquid chromatography (VLC) was carried out on silica gel (15 μm, Merck); a LiChroprep RP-18 (40–63 μm, Merck) stationary phase was used for reversed-phase VLC. Medium-pressure liquid chromatography (MPLC) was processed with a Combi Flash Rf+ Lumen instrument (Teledyne Isco) on a reversed-phase RediSep Rf HP Gold (50 g) column. Sephadex LH-20 (25–100 μm, Sigma–Aldrich) was used for gel filtration. HPLC was carried out on a Waters HPLC, using a reversed-phase (Phenomenex, Kinetex 5 μm C18 100A) column. For the investigation of compounds with chiral carbon atoms, a Lux amylose-1 column (250 × 21.2 mm) (Phenomenex, Torrance, CA, USA) was used with cyclohexane-isopropanol 85:15 as the mobile phase. All solvents used for column chromatography (CC) were of at least analytical grade (VWR Ltd., Debrecen, Hungary).

NMR spectra (Figures S1–S78) were recorded in CDCl_3_ and methanol-*d*_4_ on a Bruker Avance DRX 500 spectrometer at 500 MHz (^1^H) and 125 MHz (^13^C). The signals of the deuterated solvents were considered reference points. Chemical shifts (*δ*) of the reported compounds are given in ppm, and coupling constant values (*J*) are reported in Hz. The high-resolution MS spectra were acquired on a Thermo Scientific Q-Exactive Plus Orbitrap mass spectrometer equipped with an ESI ion source in positive ionization mode. The data were acquired and processed with MassLynx software.

### 2.2. Plant Material

*Juncus ensifolius* Wikstr. was bought from a horticultural company (Mocsáry Évelőkertészet, Tárnok, Hungary) in August 2019. A voucher specimen (No. 890) has been deposited in the Herbarium of Department of Pharmacognosy, University of Szeged, Szeged, Hungary.

### 2.3. Extraction and Isolation

The air-dried whole plant of *J. ensifolius* (1.62 kg) was thoroughly percolated with methanol (MeOH, 50 L) at room temperature. The methanolic extract was concentrated (300 g) under reduced pressure, and solvent–solvent partitioning was applied by using hexane (8 × 0.5 L), chloroform (CHCl_3_, 10 × 0.5 L), and ethyl acetate (EtOAc, 8 × 0.5 L).

The concentrated CHCl_3_-soluble fraction (12 g) was separated by VLC on silica gel using a gradient solvent system of cyclohexane–EtOAc–MeOH [from 98:2:0 to 1:1:1] to collect 14 major fractions (A–N). The result of the fractionation procedure was investigated by a TLC chromatograph, and fractions containing phenanthrenes of similar polarity were combined accordingly. In the second step, all major fractions were further separated by gel chromatography on a Sephadex LH-20 stationary phase using CH_2_Cl_2_–MeOH (1:1) as the eluent. Fractions A/2, F/3, and H/4 were pure and yielded compounds **14** (5.8 mg) from B/2, **15** (51 mg) from F/3, and compound **9** (8.4 mg) from H/4. Fraction B/2 was separated by reversed-phase (RP) HPLC under isocratic conditions, using MeOH–H_2_O (78:22 in 10 min; flow rate 1 mL/min) as the mobile phase, and one compound was obtained **4** (*t*_R_ = 6.8 min, 2.5 mg). Purification of fraction D/2 was performed by RP-HPLC under gradient conditions using MeOH–H_2_O (from 8:2 to 93:3 in 10 min, then washed with pure MeOH in 1 min; flow rate 1 mL/min) as the mobile phase to yield compounds **11** (*t*_R_ = 8.4 min, 1.8 mg), **2** (*t*_R_ = 9.8 min, 3.0 mg), and **10** (*t*_R_ = 10.4 min, 1.5 mg). After gel filtration of fraction E on Sephadex LH-20, fraction E/4 was further separated by RP-MPLC by MeOH–H_2_O gradient elution (from 1:9 to 1:0), and then subfraction E/4/3 was purified by RP-HPLC using a MeOH–H_2_O solvent system (from 82:18 to 86:14 in 10 min; flow rate 1 mL/min) as the mobile phase to yield compound **12** (*t*_R_ = 10.6 min, 1.1 mg). Fraction E/5 was separated by RP-HPLC under gradient conditions using MeOH–H_2_O (from 75:25 to 81:19 in 10 min; flow rate 1 mL/min) as the mobile phase to yield compounds **18** (*t*_R_ = 5.5 min, 100.3 mg) and **5** (*t*_R_ = 6.4 min, 13.0 mg). Fraction E/6 was also purified by RP-HPLC using MeOH–H_2_O (from 78:22 to 1:0 in 10 min; flow rate 1 mL/min) as the mobile phase to yield compound **13** (*t*_R_ = 7.2 min, 1.0 mg). Fraction F/2 was purified by RP-MPLC using MeOH–H_2_O (from 1:9 to 1:0), and then subfraction F/2/3 was further purified by RP-HPLC under gradient conditions using MeCN–H_2_O (from 1:1 to 7:3 in 10 min; flow rate 1 mL/min) as the mobile phase to yield compounds **3** (*t*_R_ = 3.7 min, 7.3 mg) and **17** (*t*_R_ = 5.2 min, 8.2 mg). Fraction G/3 was separated by RP-HPLC under gradient conditions using MeCN–H_2_O (from 4:6 to 55:45 in 10 min; flow rate 1 mL/min) as the mobile phase to yield compound **7** (*t*_R_ =5.3 min, 2.1 mg). Fraction G/4 was also separated by RP-HPLC using MeOH–H_2_O (from 4:6 to 65:35 in 10 min; flow rate 1 mL/min) as the mobile phase to yield compound **16** (*t*_R_ = 7.75 min, 2.1 mg). Purification of fraction I/3 by RP-HPLC under gradient conditions (MeCN–H_2_O from 1:1 to 8:2 in 10 min; flow rate 1 mL/min) yielded compound **8** (*t*_R_ = 2.7 min, 2.5 mg). Fraction L/3 was separated by RP-HPLC under gradient conditions using MeCN–H_2_O (from 35:65 to 7:3 in 12 min; flow rate 1 mL/min) as the mobile phase to yield compounds **6** (*t*_R_ = 5.15 min, 4.7 mg) and **1** (*t*_R_ = 10.75 min, 2.0 mg). After gel filtration, fraction M was pure and yielded luteolin (4.2 mg) from M/2.

### 2.4. Physical Characteristics of New Compounds

*Ensifolin A* (**1**): light-yellow amorphous solid; [α${]}_{D}^{25}$ +9 (*c* 0.10, MeOH); ^1^H and ^13^C-NMR data (CD_3_OD, see Table 1); HRESIMS *m*/*z* 551.1708 [M − H_2_O + H]^+^ (calcd for C_33_H_27_O_8_, 551.1706).

*Ensifolin* B (**2**): light-yellow amorphous solid; [α${]}_{D}^{25}$ +2 (*c* 0.10, MeOH); ^1^H and ^13^C-NMR data, see Table 1; HRESIMS *m*/*z* 369.1497 [M − H_2_O − H]^−^ (calcd for C_25_H_21_O_3_, 369.1491).

*Ensifolin* C (**3**): white amorphous solid; [α${]}_{D}^{25}$ +2 (*c* 0.20, MeOH); ^1^H and ^13^C-NMR data, see Table 1; HRESIMS *m*/*z* 265.1265 [M + H]^+^ (C_18_H_19_O_2_, calcd for 265.1234).

*Ensifolin* D (**4**): light-yellow amorphous powder; ^1^H and ^13^C-NMR data, see Table 1; HRESIMS *m*/*z* 281.1540 [M + H]^+^ (C_19_H_21_O_2_, calcd for 281.1536).

*Ensifolin* E (**5**): yellow amorphous solid; ^1^H and ^13^C-NMR data, see Table 2; HRESIMS *m*/*z* 267.1379 [M + H]^+^ (C_18_H_19_O_2_, calcd for 267.1380).

*Ensifolin* F (**6**): light-yellow amorphous powder; ^1^H and ^13^C-NMR data, see Table 2; HRESIMS *m*/*z* 281.1214 [M + H]^+^ (C_18_H_19_O_3_, calcd for 281.1183).

*Ensifolin* G (**7**): light-yellow amorphous granules; ^1^H and ^13^C-NMR data, see Table 3; HRESIMS *m*/*z* 281.1213 [M + H]^+^ (C_18_H_19_O_3_, calcd for 281.1183).

*Ensifolin* H (**8**): yellow amorphous powder; ^1^H and ^13^C-NMR data, see Table 3; HRESIMS *m*/*z* 281.1174 [M + H]^+^ (C_18_H_17_O_3_, calcd for 281.1172).

*Ensifolin* I (**9**): yellow amorphous powder; ^1^H and ^13^C-NMR data, see Table 3; HRESIMS *m*/*z* 263.1069 [M + H]^+^ (C_18_H_15_O_2_, calcd for 263.1067).

*Ensifolin* J (**10**): light-yellow amorphous powder; ^1^H and ^13^C-NMR data, see Table 4; HRESIMS *m*/*z* 531.2516 [M + H]^+^ (C_36_H_34_O_4_, calcd for 531.2530).

*Ensifolin* K (**11**): light-yellow amorphous powder; ^1^H and ^13^C-NMR data, see Table 5; HRESIMS *m*/*z* 529.2366 [M + H]^+^ (C_36_H_33_O_4_, calcd for 529.2373).

*Ensifolin* L (**12**): light-yellow amorphous powder; ^1^H and ^13^C-NMR data, see Table 5; HRESIMS *m*/*z* 529.2437 [M + H]^+^ (C_36_H_35_O_4_, calcd for 529.2384).

Ensifolin M (**13**): light-yellow amorphous powder; ^1^H and ^13^C-NMR data, see Table 4; HRESIMS *m*/*z* 525.2118 [M + H]^+^ (C_36_H_30_O_4_, calcd for 525.2071).

### 2.5. Antiproliferative Assays

#### 2.5.1. Cell Lines

The human colon adenocarcinoma cells (COLO 205 sensitive and the resistant COLO 320/MDR-LRP expressing P-gp), namely, ATCC-CCL-220.1 (COLO 320) and CCL-222 (COLO 205) were cultured in RPMI 1640 medium supplemented with 10% heat-inactivated fetal bovine serum, 2 mM L-glutamine, 1 mM Na pyruvate, and 100 mM HEPES. HeLa (ATCC^®^ CCL-2™) human cervix carcinoma cells, and MRC-5 (ATCC^®^ CCL-171) human embryonal lung fibroblast cells were cultured in Eagle’s minimal essential medium (EMEM, containing 4.5 g/L glucose) supplemented with a non-essential amino acid mixture, a selection of vitamins, and 10% heat-inactivated fetal bovine serum. The cell lines were detached with 0.25% trypsin and 0.02% EDTA for 5 min at 37 °C. All cell lines were purchased from LGC Promochem, Teddington, England.

#### 2.5.2. Antiproliferative Assay

In the study, human colonic adenocarcinoma cell lines (doxorubicin-sensitive COLO 205 and multidrug resistant COLO 320 colonic adenocarcinoma cells), HeLa human cervix carcinoma cells and the MRC-5 non-cancerous human embryonic lung fibroblast cell line were used to determine the effect of the compounds on cell growth. The effects of increasing concentrations of compounds on cell growth were tested in 96-well flat-bottomed microtiter plates. The stock solutions of the compounds were prepared in DMSO, and in the final samples, the DMSO content was always lower than 1%. The compounds were diluted in a volume of 100 μL of the medium. The adherent cells were cultured in 96-well flat-bottomed microtiter plates using EMEM supplemented with 10% heat-inactivated fetal bovine serum. The density of the cells was adjusted to 6 × 10^3^ cells in 100 µL per well, the cells were seeded prior to the assay for 24 h at 37 °C, with 5% CO_2_, and then the medium was removed from the plates, and fresh medium (100 µL per well) was added to the cells. The effects of increasing concentrations of compounds on cell proliferation were tested in 96-well flat-bottomed microtiter plates. The compounds were diluted in the appropriate medium; the dilutions of compounds were performed in separate plates and then added to the cells. The starting concentration of the compounds was 100 µM, and two-fold serial dilution was performed (concentration range: 100–0.19 µM). The culture plates were incubated at 37 °C for 72 h; at the end of the incubation period, 20 µL of MTT (thiazolyl blue tetrazolium bromide, Sigma) solution (from a stock solution of 5 mg/mL) was added to each well. After incubation at 37 °C for 4 h, 100 µL of sodium dodecyl sulfate (SDS) (Sigma) solution (10% in 0.01 M HCI) was added to each well, and the plates were further incubated at 37 °C overnight. Cell growth was determined by measuring the optical density (OD) at 540/630 nm, with a Multiscan EX ELISA reader (Thermo Labsystems, Cheshire, WA, USA). Inhibition of cell growth (expressed as IC_50_: inhibitory concentration that reduces the growth of the cells exposed to the tested compounds by 50%) was determined from the sigmoid curve where 100 − ((OD_sample_ − OD_medium control_)/(OD_cell control_ − OD_medium control_)) × 100 was plotted against the logarithm of the compound concentrations. Curves were fitted by Prism5 software (GraphPad Software Inc., San Diego, CA, USA) from four parallel experiments for each cell line. Results are expressed in terms of IC_50_, defined as the inhibitory dose that reduces the proliferation of the cells exposed to the tested compounds by 50% [20].

#### 2.5.3. Drug Combination Assay

The HeLa cell line was used to perform this assay. Doxorubicin (2 mg/mL, Teva Pharmaceuticals, Budapest, Hungary) was serially diluted in the horizontal direction, starting with 8.6 µM. The resistance modifier was subsequently diluted in the vertical direction, and the starting concentration was determined based on the IC_50_. The dilutions of doxorubicin were made in a horizontal direction in 100 µL, and the dilutions of the resistance modifiers occurred vertically in the microtiter plate in a 50 µL volume. The compounds and doxorubicin were diluted separately. The density of the cells was 6 × 10^3^ cells in 100 µL per well, the cells were seeded prior to the assay for 24 h at 37 °C with 5% CO_2_, and then the medium was removed from the plates, and fresh medium, 50 µL per well, was added to the cells. Then, diluted compounds with a volume of 50 μL were added to each well to reach a final volume of 200 μL. The plates were incubated for 72 h at 37 °C in a CO_2_ incubator, and at the end of the incubation period, the cell growth was determined by the MTT staining method, as described earlier. Drug interactions were evaluated using CompuSyn software [21]. Each dose-response curve (for individual agents as well as combinations) was fit to a linear model using the median effect equation in order to obtain the median effect value (corresponding to the IC_50_) and slope (m) [22,23]. The goodness-of-fit was assessed using the linear correlation coefficient, r, and only data from analysis with r > 0.90 are presented. The extent of the interaction between drugs was expressed using the combination index in which a CI value close to 1 indicates additivity, while CI < 1 is defined as synergy and CI > 1 as antagonism.

## 3. Results and Discussion

**Figure 1:**
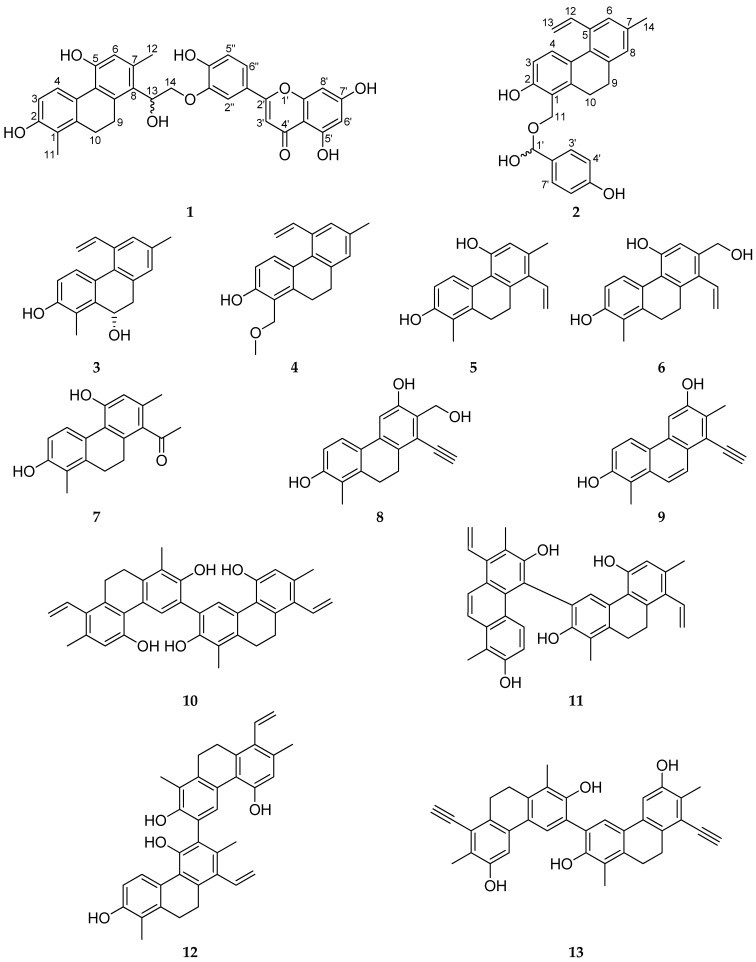
Structures of new compounds (**1**–**13**) isolated from *J. ensifolius*.

The dried whole-plant material (1.62 kg) was extracted with MeOH at room temperature. After evaporation, the extract was dissolved in 50% aqueous MeOH, and solvent–solvent partition was performed with hexane, CHCl_3_, and EtOAc. The CHCl_3_ phase was separated by a combination of different chromatographic methods, including VLC, MPLC, gel filtration, and HPLC to yield 19 compounds (Figure 1). The structure elucidation was carried out by extensive spectroscopic analysis, using NMR and HRESIMS measurements, and comparison of the spectral data with literature values.

### 3.1. Structure Elucidation of the Isolated Compounds

#### 3.1.1. Ensifolin A (**1**)

Compound **1** was isolated as light-yellow amorphous solid. Its HRESIMS peak at *m*/*z* 551.1708 [M - H_2_O + H]^+^ (calcd for 551.1706) suggested a molecular formula C_33_H_27_O_8_. The ^1^H NMR spectrum (Table 1) exhibited resonances of an *ortho*- (*δ*_H_ 7.97 and 6.66, each 1H, d, *J* = 8.6 Hz) and a *meta*-coupled (*δ*_H_ 6.47 and 6.23, each 1H, d, *J* = 2.1 Hz) pair of aromatic protons, the signals of a 1,3,4-trisubstituted aromatic ring (*δ*_H_ 7.57, 1H, d, *J* = 2.2 Hz; *δ*_H_ 7.53, 1H, dd, *J* = 8.6 and 2.2 Hz; *δ*_H_ 7.06, 1H, d, *J* = 8.6 Hz), two aromatic singlets (*δ*_H_ 6.67 and 6.63), two methylenes (*δ*_H_ 2.87 and 2.77, each 1H, m; *δ*_H_ 2.64, 2H, m), two methyls (*δ*_H_ 2.38 and 2.18, each 3H, s), and a mutually coupled oxymethine (*δ*_H_ 5.65, 1H, dd, *J* = 9.9 and 2.9 Hz) and oxymethylene group (*δ*_H_ 4.42, 1H, dd, *J* = 11.9 and 9.9 Hz; *δ*_H_ 4.32, 1H, dd, *J* = 11.9 and 2.9 Hz). The 33 carbon resonances detected in the ^13^C JMOD NMR spectrum were categorized based on their HSQC correlations and chemical shifts. A keto group at *δ*_C_ 183.9, the aforementioned *meta*-coupled aromatic methines (*δ*_H_ 6.47 d and 6.23 d, ring A) and a lone proton singlet (*δ*_H_ 6.63, ring C) attached to upfield shifted *sp*^2^ carbons (*δ*_C_ 95.2, 100.3, and 104.9, respectively), as well as the presence of a 1,3,4-trisubstituted benzene ring (C-1″–C-6″, ring B) suggested that compound **1** contains a 5,7,3′,4′-tetrahydroxyflavone structural portion. The polyphenol was readily identified as luteolin, a common tetrahydroxyflavone previously described from various *Juncus* species [24,25]. Its ^1^H and ^13^C carbon assignments were in strong agreement with literature values with the exception of small differences observed for ring B, implying that luteolin is connected to the other part of the molecule through its OH-3′ or OH-4′ group [26].

**Figure 2:**
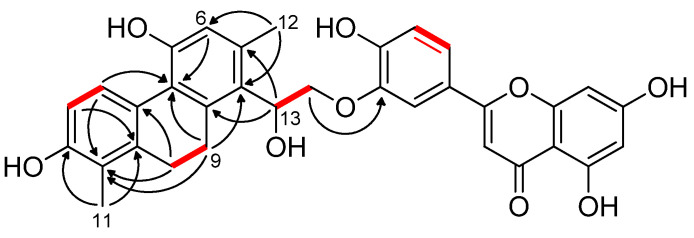
Key ^1^H-^1^H COSY (**–**) and HMBC (H→C) correlations of ensifolin A (**1**).

The remaining 18 carbons, including two saturated methylenes at *δ*_C_ 27.8 and 26.5 were reminiscent of a 9,10-dihydrophenanthrene derivative. The ^1^H–^1^H COSY spectrum defined four sequences of correlated protons, namely, a –CH=CH– (*δ*_H_ 6.66 d and 7.97 d; H-3/H-4), a –CH_2_–CH_2_– (*δ*_H_ 2.87 m (1H), 2.77 m (1H), and 2.64 m (2H); H_2_-9/H_2_-10), and a –CH(OR)–CH_2_(OR)– (*δ*_H_ 5.65 dd, 4.42 dd, and 4.32 dd; H-13/H_2_-14) fragment (Figure 2). The HMBC correlations from H-4, H_2_-9, H_2_-10, and H_3_-11 to C-1a (*δ*_C_ 139.5), from H-3 and H_2_-10 to C-4a (*δ*_C_ 125.8), and from H-4, H-6, and H_2_-9 to C-5a (*δ*_C_ 123.2) established the phenanthrene skeleton (Figure 2). According to the long-range heteronuclear correlations between H_3_-11 and C-1a, C-1 (*δ*_C_ 121.1), C-2 (*δ*_C_ 155.0), and between H-4 and C-2, a methyl and a hydroxy group was placed onto C-1 and C-2, respectively. In a similar manner, HMBC interactions of H-6 with C-5 (*δ*_C_ 155.3) and of H_3_-12 (*δ*_H_ 2.38) with C-6 (*δ*_C_ 118.5), C-7 (*δ*_C_ 136.8), and C-8 (*δ*_C_ 122.1) revealed the presence of a further hydroxy on C-5 and a methyl group on C-7. Additional HMBC correlations H-13/C-7, H-13/C-8, H-13/C-8a (*δ*_C_ 140.9), H-6/C-8, and H_2_-9/C-8 dictated that the H-13–H_2_-14 [–CH(OH)–CH_2_(OR)–] moiety is situated on C-8. The side chain presumably originated from a vinyl group, which is characteristic of many phenanthrenes isolated from Juncaceae plants. The structure of this new phenanthrene found in compound **1** was determined as 2,5-dihydroxy-8-(1-hydroxyethyl)-1,7-dimethyl-9,10-dihydrophenanthrene. The NOE cross peaks H_3_-11/H_2_-10, H_3_-12/H-6, H_3_-12/H-13, and H-13/H_2_-9 were consistent with the proposed structure, as depicted in Figure 2. Furthermore, a three-bond HMBC correlation between H_2_-14b (*δ*_H_ 4.32) and C-3” (*δ*_C_ 145.0) demonstrated that the phenanthrene and luteolin units are linked together by an ether bond formed between C-14 and C-3”.

Compound **1** has an asymmetric carbon atom (C-14). The specific rotation value [α${]}_{D}^{25}$ of the compound was +9 (*c* 0.1, MeOH). When ensifolin A (**1**) was injected onto a chiral HPLC column, it eluted with two well-separated peaks with a peak ratio area of 1:1. The peaks exhibited the same UV spectra, suggesting that **1** is a racemic mixture, with the structure shown in Figure 2. To the best of our knowledge, this is the first time that a naturally occurring phenanthrene-flavonoid conjugate is reported from the plant kingdom.

#### 3.1.2. Ensifolin B (**2**)

Compound **2** (ensifolin B) has the molecular formula C_25_H_24_O_4_ compatible with the fragment ion in the HRESIMS at *m*/*z* 369.1497 [M − H_2_O − H]^−^ (calcd for 369.1491). The ^1^H NMR spectrum displayed the typical signals of a vinyl-substituted 9,10-dihydrophenanthrene, together with resonances of an isolated oxymethylene (*δ*_H_ 5.19 and 5.09, each 1H, d, *J* = 14.5 Hz; *δ*_C_ 66.2), a hemiacetal group (*δ*_H_ 5.94, 1H, s; *δ*_C_ 98.7), and a *para*-disubstituted benzene ring (*δ*_H_ 7.50 and 6.89, each 2H, d, *J* = 8.5 Hz). The structure of the phenanthrene skeleton was assembled through 2D NMR analysis. It was concluded that the phenanthrene core of compound **2** is identical to sylvaticin A, a 9,10-dihydrophenanthrene recently described from *Luzula sylvatica* [27]. However, NMR characteristics of the H_2_-11 oxymethylene in **2** are different compared to those of sylvaticin A, including its upfield shifted carbon (*δ*_C_ 66.2 vs. 60.2) and magnetically inequivalent protons (*δ*_H_ 5.19 and 5.09, vs. *δ*_H_ 5.01, 2H, s). These findings, in conjunction with HMBC interactions from H_2_-11 to the deshielded hemiacetal carbon (*δ*_C_ 98.7), from H-7′ (*δ*_H_ 5.94) to C-3′/C-7′ (*δ*_C_ 128.2) and from H-3′/H-7′ (*δ*_H_ 7.50) to C-5′ (*δ*_C_ 156.7), unequivocally demonstrated that OH-11 and a 4-hydroxybenzaldehyde unit participated in the formation of an acyclic hemiacetal moiety. Similar to compound **1**, ensifolin B (**2**) also has an asymmetric carbon atom (C-1′). The specific rotation value [α${]}_{D}^{25}$ of the compound was +2 (*c* 0.1, MeOH). When compound **2** was injected onto a chiral HPLC column, it eluted with two well-separated peaks with a peak ratio area of 1:1. The peaks exhibited the same UV spectra, suggesting that **2** is a racemic mixture. Intermolecular hemiacetals are intrinsically unstable with respect to their parent alcohols and aldehydes. Indeed, the initially pure phenanthrene showed signs of decomposition, as two sets of proton signals (in an approximate 1:0.6 ratio) emerged in the ^1^H NMR spectrum when measured again one day later. Considering that sylvaticin A and 4-hydroxybenzaldehyde, the minor compounds of the mixture were also isolated from other fractions (compound **16** and **18**, respectively), it is unclear whether these phytochemicals originally presented in the harvested plant material or whether they are just by-products of the decomposition of compound **2**.

#### 3.1.3. Ensifolin C (**3**)

Compound **3** was obtained as a white, amorphous solid. The HRESIMS peak of the protonated molecule at *m*/*z* 265.1265 [M + H]^+^ (C_18_H_19_O_2_, calcd for 265.1234) established a molecular formula C_18_H_18_O_2_. Analysis of the NMR spectra yielded a 9,10-dihydrophenanthrene skeleton containing a rare 10-OH group (*δ*_H10_ 5.10, 1H, br t, *J* = 2.9 Hz; *δ*_c10_ 64.3). Comparison with literature data showed that ensifolin C is the 2-demethyl derivative of sylvaticin B, which was isolated from *L. sylvatica* [27]. The structure of compound **3** was therefore determined to be 2,10-dihydroxy-1,7-dimethyl-5-vinyl-9,10-dihydrophenanthrene. Investigation of the compound on a chiral HPLC column resulted in only one peak. According to literature data on similar 10-hydroxyphenanthrenes, the configuration of C-10 can be assumed as (*S*) [27,28].

#### 3.1.4. Ensifolin D (**4**)

Compound **4** was isolated as a light-yellow amorphous powder, and the formula C_19_H_20_O_2_ was assigned to it based on its protonated molecular peak at *m*/*z* 281.1540 [M + H]^+^ (calcd for 281.1536) in the HRESIMS. The 1D NMR spectra implied that the chemical structure of compound **4** is very similar to that of sylvaticin A. The upfield shifted C-11 (*δ*_C_ 66.5 vs. 56.7 in methanol-*d*_4_), as well as a diagnostic HMBC interaction between a methoxy function (*δ*_H_ 3.40, 3H, s; *δ*_C_ 66.5) and H_2_-11 (*δ*_H_ 4.66, 2H, s) dictated that ensifolin D is the 11-methoxy derivative of sylvaticin A.

#### 3.1.5. Ensifolin E (**5**)

Compound **5** has the molecular formula C_18_H_18_O_2_ according to its protonated molecular peak at *m*/*z* 267.1379 [M + H]^+^ in the HRESIMS (calcd for 267.1380). The ^1^H and ^13^C JMOD NMR spectra displayed the characteristic signals of a 9,10-dihydrophenanthrene scaffold (*δ*_H_ 2.60 and 2.72, each 2H, m; *δ*_C_ 26.7 and 28.5) substituted with two methyls, a vinyl side-chain, and two hydroxy groups (*δ*_C_ 153.8 and 154.7). Apart from these resonances, a lone aromatic singlet (*δ*_H_ 6.61, 1H) and two *ortho*-coupled aromatic protons (*δ*_H_ 6.65 and 7.98, each 1H, d, *J* = 8.6 Hz) were also detected in the ^1^H NMR data. The HMBC interactions of H_3_-11 with C-1, C-1a and C-2 and of H-4 with C-1a and C-2 assembled ring A. Correlations of the saturated methylenes H_2_-9 and H_2_-10 with C-1, C-1a, and C-4a connected rings A and B. The second methyl and the vinyl group are attached to the core at C-7 and C-8, respectively, as demonstrated by the H_3_-12/C-6, H_3_-12/C-7, H_3_-12/C-8, H-13/C-8a, H-14/C-8, and H_2_-9/C-8 heteronuclear long-range correlations. Further two- and three-bond HMBC interactions of H-6 (*δ*_H_ 6.61) with C-5a, C-5 (*δ*_C_ 154.7), and C-8 allowed the placement of an -OH group onto C-5 and established the final structure of **5** as 2,5-dihydroxy-1,7-dimethyl-8-vinyl-9,10-dihydrophenanthrene. The NOE cross-peaks H_2_-10/H_3_-11, H-6/H_3_-12, H-13/H_3_-12, H-13/H_2_-9, and H-14b/H_2_-9 corroborated with the proposed structure of ensifolin E.

#### 3.1.6. Ensifolin F (**6**)

The isolation process yielded compound **6** (ensifolin F) as a light-yellow amorphous powder. The molecular formula C_18_H_18_O_3_ of **6** was deduced from the HRESIMS peak at *m*/*z* 281.1214 [M + H]^+^ (calcd for 281.1183). The 1D NMR data suggested that compounds **5** and **6** are closely related to each other, with the only difference being the presence of a hydroxymethyl function in **6** (*δ*_H_ 4.58, 2H, s; *δ*_C_ 63.3) instead of a methyl. The oxymethylene protons showed HMBC correlations with C-6, C-7, and C-8, and NOE cross-peaks with H-6, H-13, and H-14b; therefore, it must be situated on C-7.

#### 3.1.7. Ensifolin G (**7**)

Compound **7** was obtained as light-yellow amorphous granules. Its HRESIMS suggested the molecular formula C_18_H_18_O_3_ through the presence of a peak at *m*/*z* 281.1213 [M + H]^+^ (calcd for 281.1183). In the 1D NMR spectra, the lack of resonances of a vinyl group and the appearance of an upfield shifted methyl (*δ*_H_ 2.46, 3H, s) and a keto carbon at *δ*_C_ 211.4 demonstrated that the vinyl part of ensifolin E (**5**) was biosynthetically converted to an acetyl moiety. Its position at C-8 (*δ*_C_ 134.3) was shown by the HMBC correlations from H_3_-14, H-6, H_3_-12, and H_2_-9 to this particular carbon. Careful analysis of the 2D NMR spectra led to the conclusion that ensifolin G is a structural isomer of juncatrin A, a 9,10-dihydrophenanthrene previously isolated from *Juncus atratus*, in which the H-6 proton and the OH-5 group are interchanged [24].

#### 3.1.8. Ensifolin H (**8**)

HRESIMS data provided the molecular formula of C_18_H_16_O_3_ for compound **8** through the peak of the protonated molecule at *m*/*z* 281.1174 (calcd for C_18_H_17_O_3_ 281.1172). Upon comparison of its 1D NMR data with those of juncatrin B, a 9,10-dihydrophenanthrene described from *J. atratus* by our research group [24], we found that the C-7 methyl group adjacent to an acetylene substituent was oxidized into a hydroxymethyl side chain. This assumption was substantiated by the HMBC correlations H_2_-12/C-6, H_2_-12/C-7, H_2_-12/C-8, H-5/C-7, and H-14/C-8 and by the absence of 12-methyl.

#### 3.1.9. Ensifolin I (**9**)

The molecular formula C_18_H_14_O_2_ was assigned to compound **9** according to the HRESIMS peak of the protonated molecule at *m*/*z* 263.1069 [M + H]^+^ (calcd for 263.1067). The signals in the ^1^H NMR spectrum were similar to those of juncatrin B except for the replacement of its saturated H_2_-9/H_2_-10 structural part by two mutually coupled olefinic protons (*δ*_H_ 7.77 and 8.16, each 1H, *J* = 9.4 Hz). The presence of a double bond between C-9 and C-10 was supported by the HMBC correlations recorded between H-9 (*δ*_H_ 8.16) and C-1a, C-5a, and C-8 and between H-10 (*δ*_H_ 7.77) and C-1, C-4a, and C-8a. The H-4/H-5, H_3_-12/H-14, H-9/H-14, and H-10/H_3_-11 NOE cross-peaks were in good agreement with the structure depicted in Figure 1.

#### 3.1.10. Ensifolin J (**10**)

Compound **10** has the molecular formula C_36_H_34_O_4_ as suggested by its protonated molecule appearing at *m*/*z* 531.2516 [M + H]^+^ (calcd for 531.2530) in the HRESIMS. The 1D NMR spectra of ensifolin J were almost superimposable with those of ensifolin E (**5**) (Table 3). However, instead of *ortho*-coupled aromatic protons, it exhibited only one proton singlet in the aromatic region at *δ*_H_ 8.12, and an additional nonprotonated *sp*^2^ carbon was also seen at *δ*_C_ 124.9. These findings, in conjunction with the HRESIMS data, clearly indicated that ensifolin J is a symmetric dimeric phenanthrene comprised of two ensifolin E units. In order to confirm the connection between them, a series of 2D NMR experiments were conducted. The upfield shifted singlet of H-4 (*δ*_H_ 8.12) gave three-bond heteronuclear correlations to C-1a, C-2, C-5a, and, most importantly, to the above-mentioned carbon (C-3′) resonating at *δ*_C_ 124.9. In conclusion, it was determined that the two ensifolin E monomers are linked together via their C-3 carbons resulting in a symmetrical dimer.

#### 3.1.11. Ensifolin K (**11**)

Compound **11**, obtained as a light-yellow amorphous powder, is a phenanthrene heterodimer with the molecular formula C_36_H_32_O_4_, as inferred by the HRESIMS peak at *m*/*z* 529.2366 [M + H]^+^ (calcd for 529.2373) and the 36 carbon resonances detected in the ^13^C JMOD NMR spectrum (Table 4). It was apparent that one of the building blocks of compound **11** is ensifolin E (**5**). The other phenanthrene monomer was identified as dehydrojuncuenin B by means of evaluation of the 2D NMR data and then by comparison of our assignments with reported literature values [29]. Taking into account that H-5 of dehydrojuncuenin B was missing, and a nonprotonated carbon at *δ*_C_ 118.6 (C-5) correlated only with the deshielded H-4′ (*δ*_H_ 7.96, 1H, s) of ensifolin E, it was concluded that the monomers are connected through a C–C bond formed between C-5 of dehydrojuncuenin B and C-3′ of ensifolin E (**5**).

#### 3.1.12. Ensifolin L (**12**)

Compound **12** has the molecular formula C_36_H_34_O_4_, suggested by its protonated molecular peak at *m*/*z* 529.2437 [M + H]^+^ (calcd for 529.2384) in the HRESIMS. A brief examination of the ^1^H NMR spectrum indicated that ensifolin L is a phenanthrene dimer composed of two ensifolin L (**5**) monomers (Table 4). Unlike ensifolin J (**10**), ensifolin L is not symmetrical, since its 1D NMR data provided two sets proton and carbon resonances ascribable to the two constructing subunits. The lack of H-6 and the presence of an upfield shifted singlet at *δ*_H_ 7.92 implied that the phenanthrene units are most likely connected by a C–C bond formed between C-6 and C-3′ of the corresponding aromatic rings C and A’. This presumption was proven unequivocally by the HMBC correlations from H_3_-12 (*δ*_H_ 2.03, 3H, s) and H-4′ (*δ*_H_ 7.92, ring A’) to C-6 (*δ*_C_ 125.3, ring C) and by a diagnostic NOE cross-peak of H_3_-12 with H-4′.

#### 3.1.13. Ensifolin M (**13**)

The molecular formula C_36_H_30_O_4_ was determined for compound **13** with the aid of an HRESIMS peak at *m*/*z* 525.2118 [M + H]^+^ (calcd for 525.2071). The ^1^H NMR spectrum contained a set of signals reminiscent of juncatrin B, but only two aromatic singlets were exhibited at *δ*_H_ 7.48 and 7.17 instead of three aromatic methines (the mutually coupled H-3/H-4, and H-5) that occur in the original compound (Table 3) [24]. This observation, in conjunction with the HRESIMS data, indicated ensifolin M to be a symmetrical phenanthrene dimer. The connectivity between C-3 and C-3′ was unambiguously determined by the HMBC correlation of H-4 (*δ*_H_ 7.48) with a nonprotonated carbon resonating at *δ*_C_ 126.8 (C-3,3′), which displayed no heteronuclear correlations with any of the other protons. The nuclear Overhauser effects H-4/H-5 and H_2_-10/H_3_-11 were in line with the depicted structure (Figure 1).

Besides the new compounds ensifolins A–M (**1**–**13**), four known phenanthrenes, namely, 2-hydroxy-1,7-dimethyl-5-vinyl-9,10-dihydrophenanthrene (**14**) [30], juncuenin B (**15**) [29], juncatrin B (**16**) [24], and sylvaticin A (**17**) [27], and 4-hydroxybenzaldehyde (**18**) and luteolin (**19**) were identified from *J. ensifolius*. The structural characterization was performed by means of HRESIMS and 1D and 2D NMR experiments and then by comparison of the ^1^H and ^13^C assignations with reported literature data. All compounds have been isolated for the first time from this plant.

### 3.2. Antiproliferative Activity of the Compounds

The antiproliferative activity of compounds (**1**–**19**) was investigated in human cancer cell lines, namely, cervical cancer (HeLa), doxorubicin-sensitive colonic adenocarcinoma COLO 205, multidrug resistant colonic adenocarcinoma COLO 320/MDR-LRP expressing P-gp (MDR1)-LRP, and human embryonal lung fibroblast MRC-5. The thiazolyl blue tetrazolium bromide (MTT) assay was used for each compound to assess the concentration required for 50% inhibition of viability of the cell population (IC_50_) (Table 6). The luteolin-substituted phenanthrene ensifolin A (**1**) was found to be the most promising component with substantial antiproliferative effects against all three tested cell lines (IC_50_ values 3.9–12.7 μM) and showed good selectivity (SI = 4.95) in the case of COLO 205 cells. It was more than ten-fold as active as the positive control cisplatin in COLO 205 cells. Interestingly, luteolin (**19**) alone and compound **7** (ensifolin G), structurally very similar to the phenanthrene unit of ensifolin A (**1**), were inactive for all tested cell lines. The lowest IC_50_ values against cervical carcinoma (HeLa) cells were found for compounds **15** (IC_50_ = 6.67 ± 0.03 µM) and **16** (IC_50_ = 6.65 ± 0.10 µM). The only difference between the two compounds is the substituent at C-8, which is a vinyl group in the case of **15**, and an acetylene group in **16**. Ensifolin E (**5**), differing from juncuenin B (**15**) only in the position of the hydroxy group (at C-5 in **5**, and at C-6 in **15**), resulted in a significant decrease in the activity against HeLa cells, while changing of the methyl group at C-7 in **5** to the hydroxymethylene group in ensifolin F (**6**) led to the loss of activity. Compounds **8** and **17** possessed moderate antiproliferative activity (IC_50_ values 12.31 ± 0.13 µM and 10.56 ± 0.09 µM, respectively) against HeLa cells. Ensifolin I (**9**) is the dehydro derivative of sylvaticin A (**17**), and this modification resulted in an increased activity in the cases of COLO 205 and COLO 320 adenocarcinoma cell lines, while a twofold decrease in HeLa cells. Finally, dimerization of phenanthrene monomers resulted in a decrease of the activity, as it can be seen in the case of compounds **9** and **13**, while in the case of **10** and **12**, which are the dimers of ensifolin E (**5**), neither the monomer nor its dimers showed antiproliferative activity.

The best selectivity was obtained for ensifolins D (**4**, SI > 5.15, HeLa) and H (**8**, SI > 8.13, HeLa) and for compounds **15** (SI > 5.37, HeLa), **16** (SI > 3.91, HeLa), and **17** (SI > 9.43, HeLa).

### 3.3. Drug Combination Assay

Many types of cancers are highly resistant to the currently available chemotherapeutic agents. Therefore, new effective and well-tolerated therapy strategies are needed. One of the possibilities is the identification of new bioactive natural products. Therefore, a chemosensitivity assay was carried out by studying the in vitro interactions between the compounds and the antineoplastic drug doxorubicin, known to be transported by P-gp. Therefore, a combination chemotherapy model on human HeLa cervical carcinoma cells was performed. The combination index (CI), based on the Chou and Talalay method, was the main parameter to assess drug–drug interactions as synergistic (CI < 1), additive (CI = 1) or antagonistic (CI > 1) (Table 7) [22].

As can be observed in Table 6, all derivatives tested were found to interact synergistically with doxorubicin (CI < 1) in the HeLa cell line. Very strong synergisms were observed for ensifolins E (**5**) and H (**8**), with CI values lower than 0.1. Both compounds showed weak or moderate activity (IC_50_s 25.2–31.2 μM for **5**, and 12.3–63.5 μM for **8**) in the case of antiproliferative investigation.

## 4. Conclusions

In this investigation, 17 phenanthrenes, among them ensifolins A–M (**1**–**13**) as new natural products, four known ones (**14**–**17**), and 4-hydroxybenzaldehyde (**18**) and luteolin (**19**), were characterized from the whole plant of *J. ensifolius*. Their planar structures were elucidated by comprehensive spectroscopic data. All compounds were determined for the first time from the plant. Compounds **1** and **15**–**17** displayed in vitro antiproliferative activity against different tumor cell lines. The luteolin-substituted phenanthrene (**1**) was found to be the most promising component with substantial antiproliferative effects against COLO 205 cells (IC_50_ value 3.9 μM). Moreover, compounds **5** and **8** possessed very strong synergism with doxorubicin in the drug combination assay. These findings not only enrich the chemical diversity of phenanthrenes but also provide new natural small molecules for further antiproliferative investigations.

## Figures and Tables

**Table 1:** ^1^H (500 MHz) and ^13^C (125 MHz) NMR data of compounds **1**–**3**.

Position	1 ^a^		2 ^b^		3 ^a^	
	δ_H_ (*J* in Hz)	δ_C_, Type	δ_H_ (*J* in Hz)	δ_C_, Type	δ_H_ (*J* in Hz)	δ_C_, Type
1		121.1, C		117.5, C		123.5, C
1a		139.5, C		135.9, C		139.4, C
2		155.0, C		152.4, C		155.9, C
3	6.66, d (8.6)	112.8, CH	6.86, d (8.6)	114.1, CH	6.76, d (8.4)	114.3, CH
4	7.97, d (8.6)	128.1, CH	7.53, d (8.6)	129.3, CH	7.38, d (8.4)	129.2, CH
4a		125.8, C		127.6, C		126.4, C
5a		123.2, C		130.6, C		132.0, C
5		155.3, C		135.3, C		136.0, C
6	6.67, s	118.5, CH	7.25, br s	127.7, CH	7.21, br s	128.5, CH
7		136.8, C		136.3, C		136.8, C
8		122.1, C	7.02, br s	127.9, CH	7.03, br s	130.5, CH
8a		140.9, C		138.5, C		134.8, C
9	2.77, m 2.87, m	27.8, CH_2_	2.73, m (2H)	29.6, CH_2_	2.84, dd (16.0, 3.0) 3.05, dd (16.0, 2.8)	39.1, CH_2_
10	2.64, m (2H)	26.5, CH_2_	2.54, m (2H)	24.1, CH_2_	5.10, br t (2.8)	64.3, CH
11	2.18, s (3H)	11.6, CH_3_	5.09, d (14.5) 5.19, d (14.5)	66.2, CH_2_	2.33 *, s (3H)	11.1, CH_3_
OCH_3_–11						
12	2.38, s (3H)	20.7, CH_3_	6.98, dd (17.4, 10.9)	138.9, CH	7.02, dd (17.4, 10.9)	140.9, CH
13	5.65, dd (9.9, 2.9)	75.6, CH	5.28, dd (10.9, 1.3) 5.72, dd (17.4, 1.3)	114.7, CH_2_	5.21, dd (10.9, 1.4) 5.66, dd (17.4, 1.4)	113.6, CH_2_
14	4.32, dd (11.9, 2.9) 4.42, dd (11.9, 9.9)	67.1, CH_2_	2.37, s (3H)	21.2, CH_3_	2.33 *, s (3H)	21.1, CH_3_

^a^ measured in methanol-*d*_4_; ^b^ measured in CDCl_3_; * overlapping signals; luteolin part of compound **1**: *δ*_H_—6.63 (H-3′; 1H, s), 6.23 (H-6′; 1H, d, *J* = 2.1 Hz), 6.47 (H-8′; 1H, d, *J* = 2.1 Hz), 7.57 (H-2″; 1H, d, *J* = 2.2 Hz), 7.06 (H-5″; 1H, d, *J* = 8.6 Hz), 7.53 (H-6″; 1H, dd, *J* = 8.6 and 2.2 Hz); *δ*_C_ (with types of carbons)—165.5 (C-2′; C), 104.9 (C-3′; CH), 183.9 (C-4′; C), 105.5 (C-4a’; C), 163.3 (C-5′; C), 100.3 (C-6′; CH), 166.3 (C-7′; C), 95.2 (C-8′; CH), 159.5 (C-8a’; C), 125.6 (C-1″; C), 116.6 (C-2″; CH), 145.0 (C-3″; C), 148.6 (C-4″; C), 119.2 (C-5″; CH), 121.3 (C-6″; CH); hemiacetal part originated from 4-hydroxybenzaldehyde of compound **2**: *δ*_H_—5.94 (H-1′; 1H, s), 7.50 (H-3′/H-7′; 2H, d, *J* = 8.5 Hz), 6.89 (H-4′/H-6′; 2H, d, *J* = 8.5 Hz); *δ*_C_ (with types of carbons)—98.7 (C-1′; CH), 129.8 (C-2′; C), 2 × 128.2 (C-3′/C-7′; 2 × CH), 2 × 115.5 (C-4′/C-6′; 2 × CH), 156.7 (C-5′; C).

**Table 2:** ^1^H (500 MHz) and ^13^C (125 MHz) NMR data of compounds **4**–**6** (in methanol-*d*_4_).

Position	4		5		6	
	δ_H_ (*J* in Hz)	δ_C_, Type	δ_H_ (*J* in Hz)	δ_C_, Type	δ_H_ (*J* in Hz)	δ_C_, Type
1		121.9, C		121.2, C		121.2, C
1a		142.8, C		139.5, C		139.7, C
2		156.5, C		154.7, C		154.9, C
3	6.71, d (8.5)	113.0, CH	6.65, d (8.6)	112.7, CH	6.65, d (8.6)	112.7, CH
4	7.40, d (8.5)	131.6, CH	7.98, d (8.6)	127.9, CH	8.03, d (8.6)	128.1, CH
4a		127.2, C		126.5, C		126.2, C
5a		132.4, C		121.7, C		123.0, C
5		135.9, C		153.8, C		154.2, C
6	7.22, br s	128.0, CH	6.61, s	117.0, CH	6.91, s	114.9, CH
7		136.8, C		135.6, C		138.54 ^#^, C
8	7.00, br s	128.5, CH		129.4, C		128.5, C
8a		140.0, C		138.4, C		138.57 ^#^, C
9	2.66, m (2H)	31.0, CH_2_	2.72, m (2H)	28.5, CH_2_	2.74, m (2H)	28.2, CH_2_
10	2.77, m (2H)	26.5, CH_2_	2.60, m (2H)	26.7, CH_2_	2.63, m (2H)	26.6, CH_2_
11	4.66, s (2H)	66.5, CH_2_	2.18, s (3H)	11.7, CH_3_	2.19, s (3H)	11.7, CH_3_
OCH_3_–11	3.40, s (3H)	58.1, CH_3_				
12	6.92, dd (17.5, 10.9)	140.3, CH	2.22, s (3H)	20.8, CH_3_	4.58, s (2H)	63.3, CH_2_
13	5.21, dd (10.9, 1.6) 5.68, dd (17.5, 1.6)	113.8, CH_2_	6.72, dd (17.9, 11.3)	136.5, CH	6.79, dd (17.8, 11.3)	135.5, CH
14	2.33, s (3H)	21.1, CH_3_	5.09, dd (17.9, 2.3) 5.48, dd (11.3, 2.3)	119.5, CH_2_	5.17, dd (17.8, 2.2) 5.51, dd (11.3, 2.2)	120.1, CH_2_

^#^ interchangeable signals.

**Table 3:** ^1^H (500 MHz) and ^13^C (125 MHz) NMR data of compounds **7–9**.

Position	7 ^a^		8 ^a^		9 ^b^	
	δ_H_ (J in Hz)	δ_C_, Type	δ_H_ (J in Hz)	δ_C_, Type	δ_H_ (J in Hz)	δ_C_, Type
1		121.5, C		122.7, C		121.5, C
1a		139.1, C		138.7, C		139.1, C
2		155.1, C		156.5, C		155.1, C
3	6.66, d (8.6)	112.9, CH	6.72, d (8.4)	114.1, CH	6.66, d (8.6)	112.9, CH
4	8.03, d (8.6)	128.1, CH	7.38, d (8.4)	123.2, CH	8.03, d (8.6)	128.1, CH
4a		125.5, C		127.0, C		125.5, C
5a		121.8, C		137.5, C		121.8, C
5		155.5, C	7.16, s	112.2, CH		155.5, C
6	6.61, s	117.4, CH		156.1, C	6.61, s	117.4, CH
7		132.4, C		127.2, C		132.4, C
8		134.3, C		122.2, C		134.3, C
8a		135.8, C		131.3, C		135.8, C
9	2.55, m (2H)	28.3, CH_2_	2.94, m (2H)	27.2, CH_2_	2.55, m (2H)	28.3, CH_2_
10	2.68, m (2H)	26.3, CH_2_	2.76, m (2H)	26.1, CH_2_	2.68, m (2H)	26.3, CH_2_
11	2.18, s (3H)	11.7, CH_3_	2.19, s (3H)	11.5, CH_3_	2.18, s (3H)	11.7, CH_3_
12	2.17, s (3H)	19.1, CH_3_	4.93, s (2H)	60.1, CH_2_	2.17, s (3H)	19.1, CH_3_
13		211.4, C		81.1, C		211.4, C
14	2.46, s (3H)	33.1, CH_3_	3.85, s	86.4, CH	2.46, s (3H)	33.1, CH_3_

^a^ measured in methanol-*d*_4_; ^b^ measured in CDCl_3_.

**Table 4:** ^1^H (500 MHz) and ^13^C (125 MHz) NMR data of symmetric dimers **10** and **13** in methanol-*d*_4_.

Position	10		13	
	δ_H_ (J in Hz)	δ_C_, Type	δ_H_ (J in Hz)	δ_C_, Type
1, 1′		122.9, C		124.5, C
1a, 1a′		139.0, C		137.7, C
2, 2′		151.4, C		153.3 ^+^, C
3, 3′		124.9, C		126.8, C
4, 4′	8.12, s	130.3, CH	7.48, s	125.3, CH
4a, 4a′		127.4, C		128.3 ^+^, C
5a, 5a′		121.5, C		134.9, C
5, 5′		153.9, C	7.17, s	111.3, CH
6, 6′	6.60, s	117.0, CH		155.0, C
7, 7′		136.0, C		126.4, C
8, 8′		129.5, C		123.0, C
8a, 8a′		138.6, C		131.2, C
9, 9′	2.82, m (2H)	28.5, CH_2_	2.99, m (2H)	27.5, CH_2_
10, 10′	2.70, m (2H)	26.9, CH_2_	2.84, m (2H)	26.4, CH_2_
11, 11′	2.32, s (3H)	12.4, CH_3_	2.32, s (3H)	12.3, CH_3_
12, 12′	2.23, s (3H)	20.8, CH_3_	2.33, s (3H)	14.1, CH_3_
13, 13′	6.76, dd (17.9, 11.3)	136.5, CH		82.2, C
14, 14′	5.13, dd (17.9, 2.2) 5.52, dd (11.3, 2.2)	119.6, CH_2_	3.85, s	86.1, C

^+^ only seen in the HMBC spectrum.

**Table 5:** ^1^H (500 MHz) and ^13^C (125 MHz) NMR data of compounds **11** and **12** in methanol-*d*_4_.

Position	11		12	
	δH (J in Hz)	δC, Type	δH (J in Hz)	δC, Type
1		117.4, C		121.2, C
1a		134.9, C		139.82 ^#^, C
2		153.47 ^#^, C		154.9, C
3	6.53, d (9.4)	115.4, CH	6.64, d (8.6)	112.7, CH
4	7.56, d (9.4)	127.1, CH	8.02, d (8.6)	128.2, CH
4a		125.82, C		126.5, C
5a		131.0, C		122.1, C
5		118.6, C		151.3, C
6		153.37 ^#^, C		125.3, C
7		123.3, C		135.4, C
8		138.8, C		130.49, C
8a		125.5, C		137.9, C
9	8.04, d (9.5)	125.80, CH	2.87, m (2H)	28.4, CH_2_
10	7.72, d (9.5)	120.9, CH	2.69 *, m (2H)	26.8, CH_2_
11	2.47, s (3H)	11.2, CH_3_	2.21, s (3H)	11.7, CH_3_
12	2.44, s (3H)	14.5, CH_3_	2.03, s (3H)	18.5, CH_3_
13	7.19, dd (17.9, 11.4)	136.9, CH	6.82, dd (17.8, 11.2)	137.3, CH
14	5.41, dd (17.9, 2.2) 5.85, dd (11.4, 2.2)	121.8, CH_2_	5.16, dd (17.8, 2.4) 5.54, dd (11.2, 2.4)	119.9, CH_2_
1′		123.4, C		122.7, C
1a′		140.5, C		139.89 ^#^, C
2′		152.2, C		152.6, C
3′		123.6, C		120.8, C
4′	7.96, s	130.0, CH	7.92, s	130.38, CH
4a′		128.6, C		127.3, C
5a′		121.2, C		121.4, C
5′		153.9, C		153.9, C
6′	6.55, s	117.0, CH	6.61, s	117.1, CH
7′		136.2, C		136.0, C
8′		129.4, C		129.5, C
8a′		138.4, C		138.5, C
9′	2.77, m 3.05, ddd (15.1, 6.9, 4.3)	28.6, CH_2_	2.78 *, m (2H	28.8, CH_2_
10′	2.67, m 2.94, ddd (11.1, 6.9, 4.3)	27.1, CH_2_	2.67 *, m 2.77 *, m	27.0, CH_2_
11′	2.32, s (3H)	12.4, CH_3_	2.30, s (3H)	12.3, CH_3_
12′	2.22, s (3H)	20.8, CH_3_	2.23, s (3H)	20.8, CH_3_
13′	6.78, dd (17.9, 11.3)	136.4, CH	6.76, dd (17.8, 11.3)	136.4, CH
14′	5.16, dd (17.9, 2.2) 5.54, dd (11.3, 2.2)	119.7, CH_2_	5.13, dd (17.8, 2.2) 5.52, dd (11.3, 2.2)	119.6, CH_2_

* overlapping signals; ^#^ interchangeable signals reflects the chemical diversity of phenanthrenes.

**Table 6:** Antiproliferative activity (IC_50_ values) of the isolated compounds (**1**–**19**) (SI is the selectivity index).

Compound	IC_50_ (µM) ± SD				SI MRC-5/COLO 205	SI MRC-5/COLO 320	SI MRC-5/HeLa
	COLO 205	COLO 320	HeLa	MRC-5			
**1**	3.86 ± 0.08	12.71 ± 0.05	8.25 ±0.51	19.29 ± 0.54	5.00	1.52	2.34
**2**	45.64 ± 0.50	37.24 ± 0.11	33.49 ± 0.29	51.87 ± 0.14	1.14	1.39	1.55
**3**	>100	>100	>100	>100			
**4**	65.61 ± 0.78	61.56 ± 9.95	19.40 ± 0.33	>100	>1.52	>1.62	>5.15
**5**	31.23 ± 0.66	25.17 ± 0.92	27.46 ± 1.19	44.31 ± 0.61	1.42	1.76	1.61
**6**	>100	93.71 ± 0.14	74.32 ± 2.98	>100		>1.07	>1.35
**7**	>100	>100	75.57 ± 0.94	>100			>1.32
**8**	>100	63.46 ± 2.70	12.31 ± 0.13	>100		>1.58	>8.12
**9**	18.21 ± 0.28	18.52 ± 0.06	24.09 ± 0.11	49.14 ± 0.83	2.70	2.65	2.04
**10**	44.48 ± 1.22	42.76 ± 1.28	33.54 ± 1.89	57.75 ± 1.32	1.30	1.35	1.72
**11**	31.38 ± 0.72	37.84 ± 1.05	29.53 ± 0.31	33.16 ± 0.05	1.06	0.88	1.12
**12**	26.91 ± 1.19	37.36 ± 2.13	30.22 ± 0.21	50.36 ± 1.30	1.87	1.35	1.67
**13**	42.72 ± 0.92	37.27 ± 0.55	31.51 ± 0.53	72.54 ± 1.56	1.70	1.95	2.30
**14**	32.92 ± 0.59	52.36 ± 0.77	58.09 ± 1.20	60.89 ± 0.25	1.85	1.16	1.05
**15**	37.08 ± 0.57	30.54 ± 0.93	6.67 ± 0.03	35.85 ± 1.23	0.97	1.17	5.37
**16**	34.42 ± 0.57	32.48 ± 0.75	6.65 ± 0.10	26.03 ± 0.85	0.76	0.80	3.91
**17**	56.73 ± 0.75	57.66 ± 0.92	10.56 ± 0.09	>100	>1.76	>1.73	>9.47
**18**	>100	>100	>100	>100			
**19**	>100	>100	>100	>100			
DMSO	>1%	>1%	>1%	>1%			
cisplatin	41.67 ± 1.62	2.14 ± 0.32	3.62 ± 0.16	2.36 ± 0.33			
doxorubicin	1.36 ± 0.36	0.22 ± 0.004	0.04 ± 0.004	0.53 ± 0.06			

Four parallel measurements were applied for all tested compounds. SI: selectivity index; The selectivity indexes (SI) were calculated as the ratio of the IC_50_ value in the non-tumour cells and the IC_50_ in the cancer cell lines. The compound’s activity towards cancer cells is considered strongly selective if the selectivity index (SI) value is higher than 6, moderately selective if 3 < SI < 6, slightly selective if 1 < SI < 3, and non-selective if SI is lower than 1.

**Table 7:** Interaction type between doxorubicin and phenanthrenes (**1**–**17**) in HeLa cells.

Compound	CI	SD	Ratio	Interaction
**1**	0.272	0.2124	9.28:1	strong synergism
**2**	0.584	0.0510	23.2:1	synergism
**3**	0.580	0.0387	13.92:1	synergism
**4**	0.643	0.1623	55.68:1	synergism
**5**	0.001	0.0003	9.28:1	very strong synergism
**6**	0.159	0.1414	23.2:1	strong synergism
**7**	0.568	0.0268	46.4:1	synergism
**8**	0.033	0.0106	185.6:1	very strong synergism
**9**	0.180	0.0675	9.28:1	strong synergism
**10**	0.454	0.0269	38.4:1	synergism
**11**	0.112	0.0387	10.44:1	strong synergism
**12**	0.120	0.0418	11.6:1	strong synergism
**13**	0.445	0.1202	46.4:1	synergism
**14**	0.579	0.0855	92.8:1	synergism
**15**	0.864	0.2338	27.84:1	slight synergism
**16**	0.682	0.3743	13.92:1	synergism
**17**	0.279	0.0574	13.92:1	strong synergism

Combination index (CI) values are expressed as the average of CI values calculated based on different drug ratios ± standard deviation (SD) for an inhibitory concentration of 50% (IC_50_). CI < 0.1: very strong synergism; 0.1 < CI < 0.3: strong synergism; 0.3 < CI < 0.7: synergism; 0.7 < CI < 0.9: moderate to slight synergism; 0.9 < CI < 1.1: nearly additive; 1.1 < CI < 1.45: slight to moderate antagonism; 1.45 < CI < 3.30: antagonism [31].

## Data Availability

Not applicable.

## Supplementary Materials

The following supporting information can be downloaded at: https://www.mdpi.com/article/10.3390/pharmaceutics14030608/s1, Figures S1–S78: NMR spectra of isolated compounds (**1**–**13**).
