# Supplementary material for: Unique Phenanthrenes from Juncus ensifolius and Their Antiproliferative and Synergistic Effects with the Conventional Anticancer Agent Doxorubicin against Human Cancer Cell Lines
Source: Pharmaceutics. 2022 Mar 10;14(3):608. doi: 10.3390/pharmaceutics14030608 (PMC8949129; doi:10.3390/pharmaceutics14030608)
Supplement: Supplementary file 1 [file pharmaceutics-14-00608-s001.zip › pharmaceutics-1614920-supplementary.pdf]

pharmaceutics

MDPI

Supplementary Materials: Unique Phenanthrenes from *Juncus ensifolius* and Their Antiproliferative and Synergistic Effects with the Conventional Anticancer Agent Doxorubicin against Human Cancer Cell Lines

TABLE OF CONTENTS

Figure S1. ^{1}H NMR spectrum of compound **1** (500 MHz, in CD_3OD). 4

Figure S2. ^{13}C (JMOD) NMR spectrum of compound **1** (125 MHz, in CD_3OD). 4

Figure S3. HSQC spectrum of compound **1** (in CD_3OD). 5

Figure S5. HMBC spectrum of compound **1** (in CD_3OD). 6

Figure S6. NOESY spectrum of compound **1** (in CD_3OD)....................7

Figure S7. ^{1}H NMR spectrum of compound **2** (in $CDCl_3$)....................7

Figure S8. ^{13}C (JMOD) NMR spectrum of compound **2** (in $CDCl_3$)....................8

Figure S9. HSQC spectrum of compound **2** (in $CDCl_3$)....................9

Figure S10. ^{1}H-^{1}H COSY spectrum of compound **2** (in $CDCl_3$). 9

Figure S11. HMBC spectrum of compound **2** (in $CDCl_3$). 10

Figure S12. ^{1}H NMR spectrum of compound **3** (in CD_3OD)....................11

Figure S13. ^{13}C (JMOD) NMR spectrum of compound **3** (in CD_3OD)....................11

Figure S14. HSQC spectrum of compound **3** (in CD_3OD). 12

Figure S15. ^{1}H-^{1}H COSY spectrum of compound **3** (in CD_3OD). 12

Figure S16. HMBC spectrum of compound **3** (in CD_3OD). 13

Figure S17. NOESY spectrum of compound **3** (in CD_3OD)....................14

Figure S18. ^{1}H NMR spectrum of compound **4** (in CD_3OD)....................14

Figure S19. ^{13}C (JMOD) NMR spectrum of compound **4** (in CD_3OD)....................15

Figure S20. HSQC spectrum of compound **4** (in CD_3OD). 16

Figure S21. ^{1}H-^{1}H COSY spectrum of compound **4** (in CD_3OD). 16

Figure S22. HMBC spectrum of compound **4** (in CD_3OD). 17

Figure S23. NOESY spectrum of compound **4** (in CD_3OD)....................18

Figure S24. ^{1}H NMR spectrum of compound **5** (in CD_3OD)....................18

Figure S25. ^{13}C (JMOD) NMR spectrum of compound **5** (in CD_3OD)....................19

Figure S25. HSQC spectrum of compound **5** (in CD_3OD). 20

Figure S26. ^{1}H-^{1}H COSY spectrum of compound **5** (in CD_3OD). 20

Figure S27. HMBC spectrum of compound **5** (in CD_3OD). 21

Figure S28. NOESY spectrum of compound **5** (in CD_3OD)....................22

Figure S29. ^{1}H NMR spectrum of compound **6** (in CD_3OD)....22
Figure S30. ^{13}C (JMOD) NMR spectrum of compound **6** (in CD_3OD)....23
Figure S31. HSQC spectrum of compound **6** (in CD_3OD). 24
Figure S32. ^{1}H-^{1}H COSY spectrum of compound **6** (in CD_3OD). 24
Figure S33. HMBC spectrum of compound **6** (in CD_3OD). 25
Figure S34. NOESY spectrum of compound **6** (in CD_3OD)....26
Figure S35. ^{1}H NMR spectrum of compound **7** (in CD_3OD)....26
Figure S36. ^{13}C (JMOD) spectrum of compound **7** (in CD_3OD)....27
Figure S37. HSQC spectrum of compound **7** (in CD_3OD). 28
Figure S38. ^{1}H-^{1}H COSY spectrum of compound **7** (in CD_3OD). 28
Figure S39. HMBC spectrum of compound **7** (in CD_3OD). 29
Figure S40. NOESY spectrum of compound **7** (in CD_3OD)....30
Figure S41. ^{1}H NMR spectrum of compound **8** (in CD_3OD)....30
Figure S42. ^{13}C (JMOD) NMR spectrum of compound **8** (in CD_3OD)....31
Figure S43. HSQC spectrum of compound **8** (in CD_3OD). 32
Figure S44. ^{1}H-^{1}H COSY spectrum of compound **8** (in CD_3OD). 32
Figure S45. HMBC spectrum of compound **8** (in CD_3OD). 33
Figure S46. NOESY spectrum of compound **8** (in CD_3OD)....34
Figure S47. ^{1}H NMR spectrum of compound **9** (in $CDCl_3$)....34
Figure S48. ^{13}C (JMOD) NMR spectrum of compound **9** (in $CDCl_3$)....35
Figure S49. HSQC spectrum of compound **9** (in $CDCl_3$)....36
Figure S50. ^{1}H-^{1}H COSY spectrum of compound **9** (in $CDCl_3$). 36
Figure S51. HMBC spectrum of compound **9** (in $CDCl_3$). 37
Figure S52. NOESY spectrum of compound **9** (in $CDCl_3$)....38
Figure S53. ^{1}H NMR spectrum of compound **10** (in CD_3OD)....38
Figure S54. ^{13}C (JMOD) NMR spectrum of compound **10** (in CD_3OD)....39
Figure S55. HSQC spectrum of compound **10** (in CD_3OD). 40
Figure S56. ^{1}H-^{1}H COSY spectrum of compound **10** (in CD_3OD). 40
Figure S57. HMBC spectrum of compound **10** (in CD_3OD). 41
Figure S58. NOESY spectrum of compound **10** (in CD_3OD)....42
Figure S59. ^{1}H NMR spectrum of compound **11** (in CD_3OD)....42
Figure S60. ^{13}C (JMOD) NMR spectrum of compound **11** (in CD_3OD)....43
Figure S61. HSQC spectrum of compound **11** (in CD_3OD). 44
Figure S62. ^{1}H-^{1}H COSY spectrum of compound **11** (in CD_3OD). 44
Figure S63. HMBC spectrum of compound **11** (in CD_3OD). 45
Figure S64. NOESY spectrum of compound **11** (in CD_3OD)....46

Figure S65. ^{1}H NMR spectrum of compound **12** (in CD_3OD)..46
Figure S66. ^{13}C (JMOD) NMR spectrum of compound **12** (in CD_3OD)..47
Figure S67. HSQC spectrum of compound **12** (in CD_3OD). 48
Figure S68. ^{1}H-^{1}H COSY spectrum of compound **12** (in CD_3OD). 48
Figure S69. HMBC spectrum of compound **12** (in CD_3OD). 49
Figure S72. NOESY spectrum of compound **12** (in CD_3OD)..50
Figure S73. ^{1}H NMR spectrum of compound **13** (in CD_3OD)...50
Figure S74. ^{13}C (JMOD) NMR spectrum of compound **13** (in CD_3OD)..51
Figure S75. HSQC spectrum of compound **13** (in CD_3OD). 52
Figure S76. ^{1}H-^{1}H COSY spectrum of compound **13** (in CD_3OD). 52
Figure S77. HMBC spectrum of compound **13** (in CD_3OD). 53
Figure S78. NOESY spectrum of compound **13** (in CD_3OD)..54

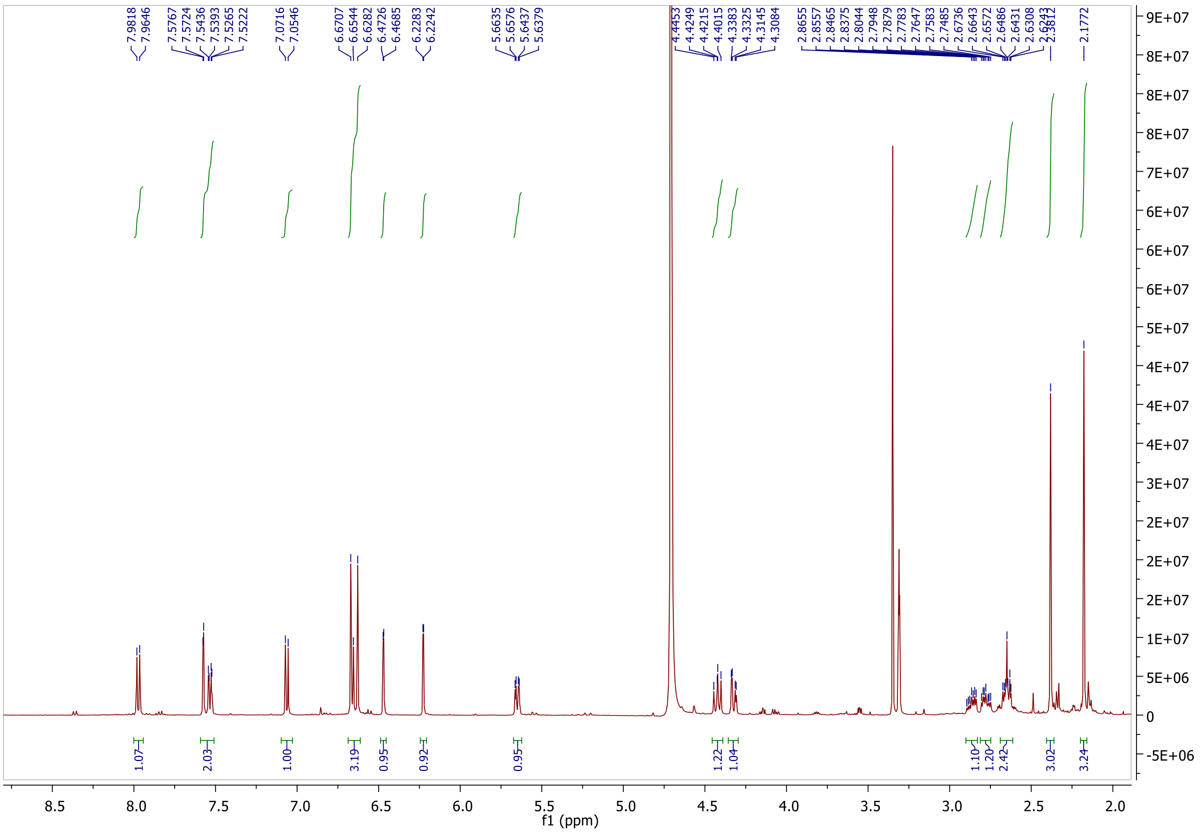

Figure S1. ^{1}H NMR spectrum of compound **1** (500 MHz, in CD_3OD).

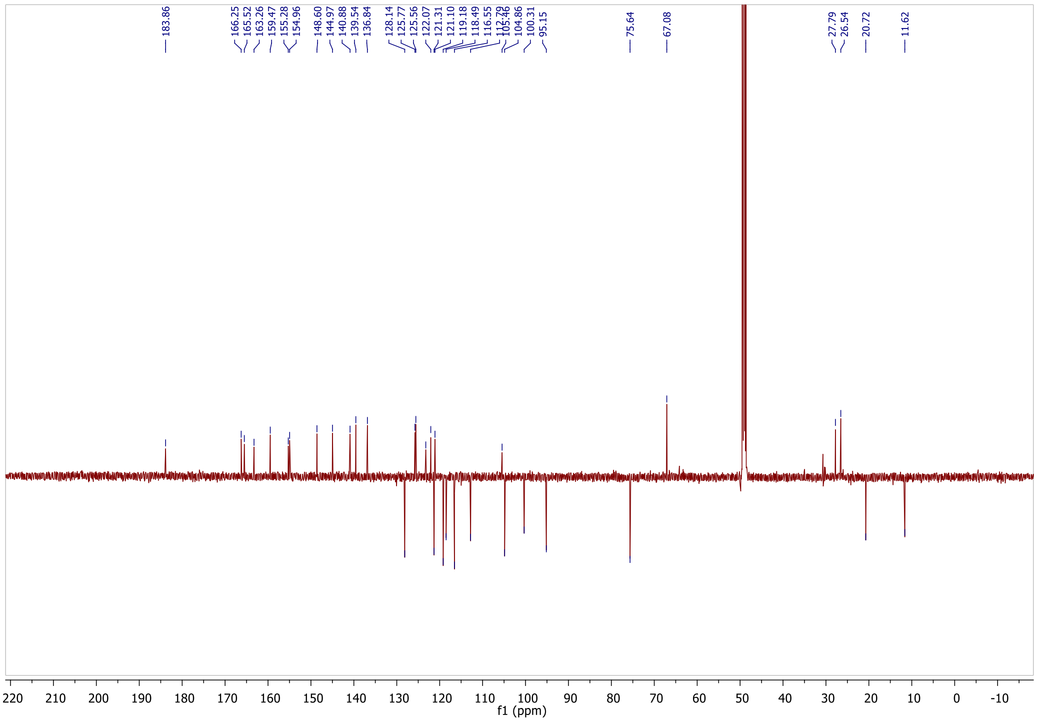

Figure S2. ^{13}C (JMOD) NMR spectrum of compound **1** (125 MHz, in CD_3OD).

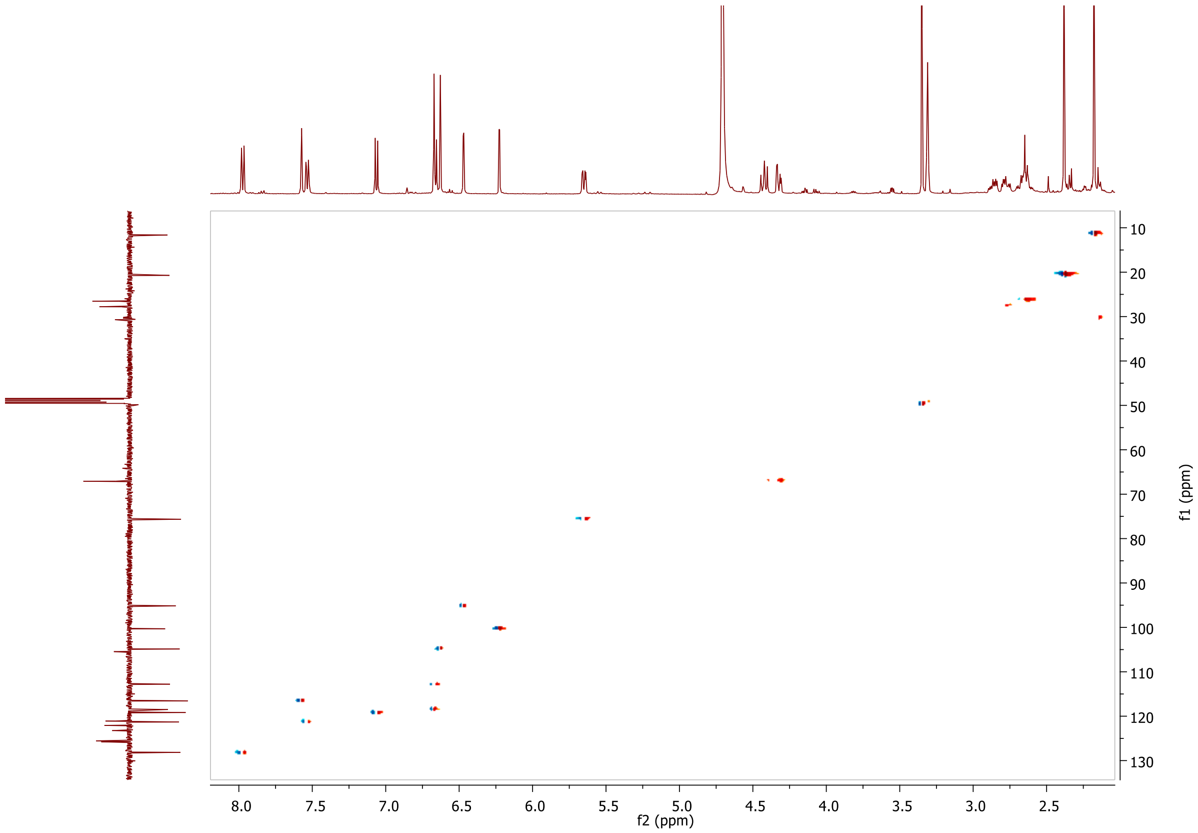

Figure S3. HSQC spectrum of compound **1** (in CD_3OD).

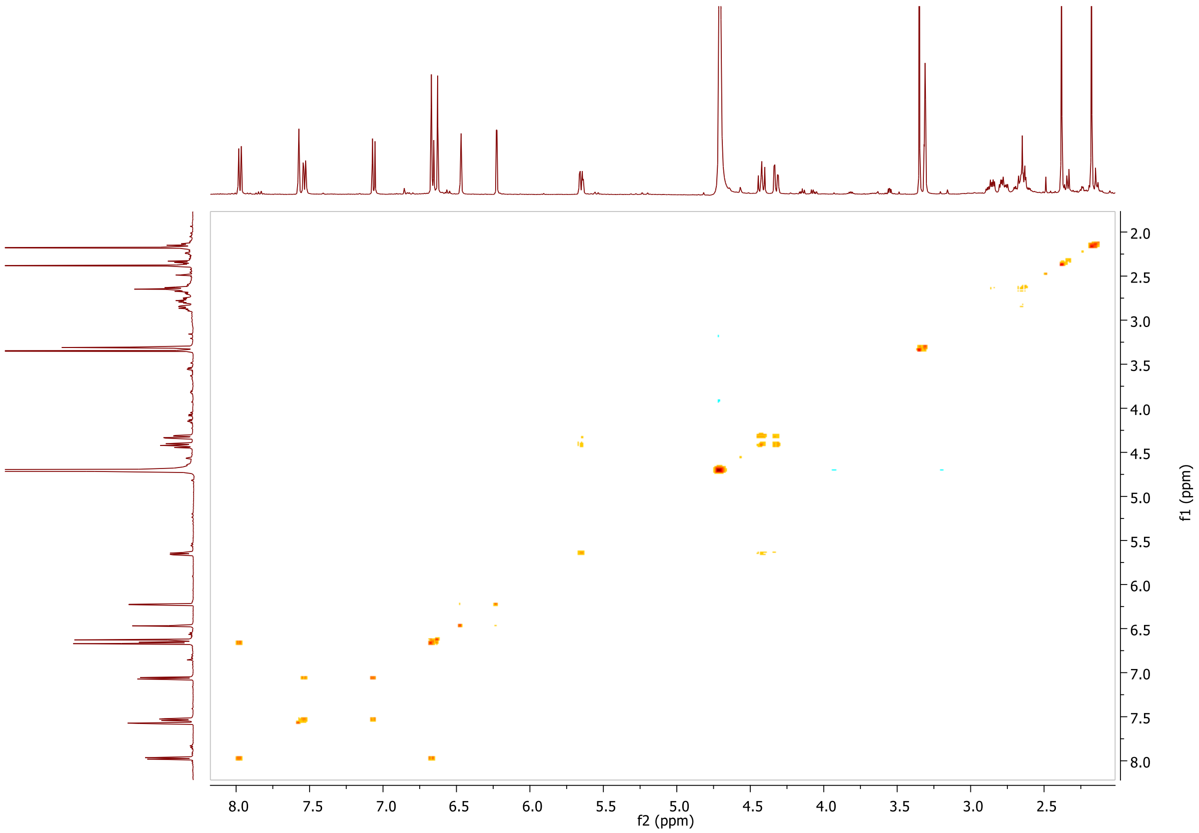

Figure S4. ^{1}H-^{1}H COSY spectrum of compound **1** (in CD_3OD).

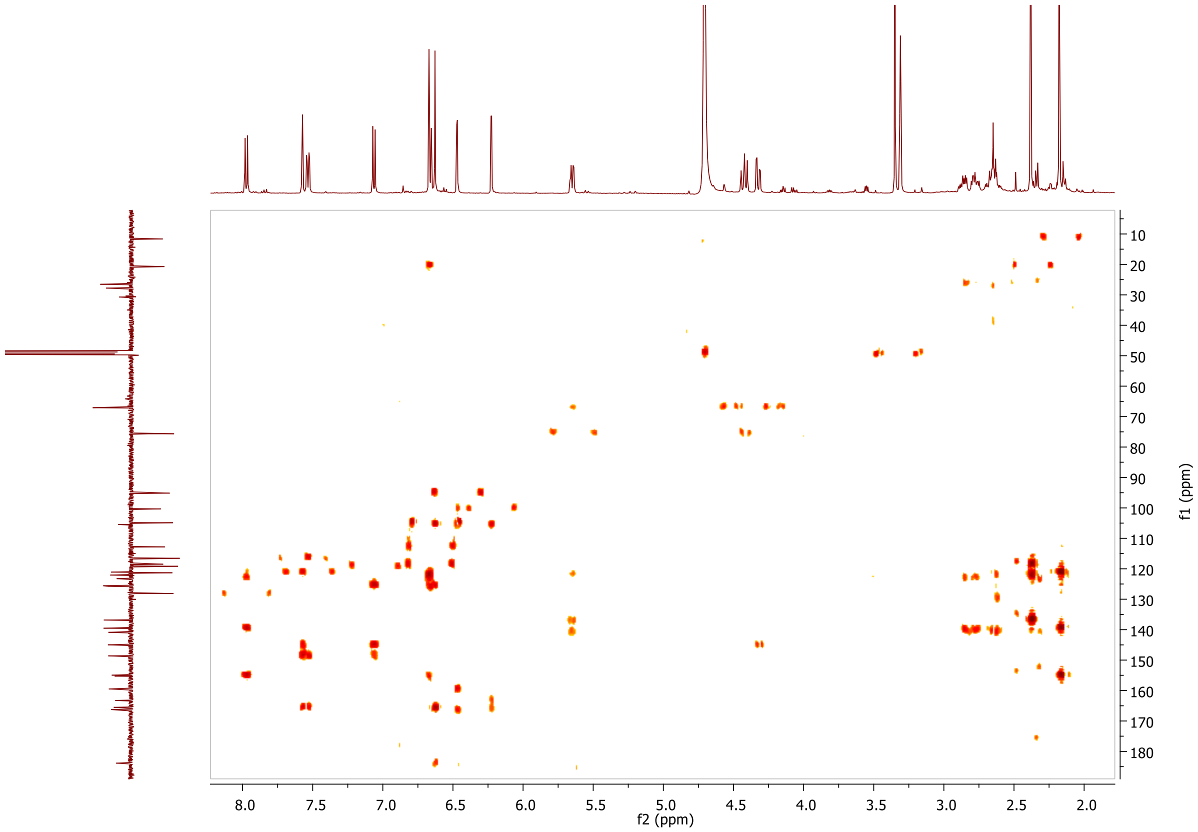

Figure S5. HMBC spectrum of compound **1** (in CD_3OD).

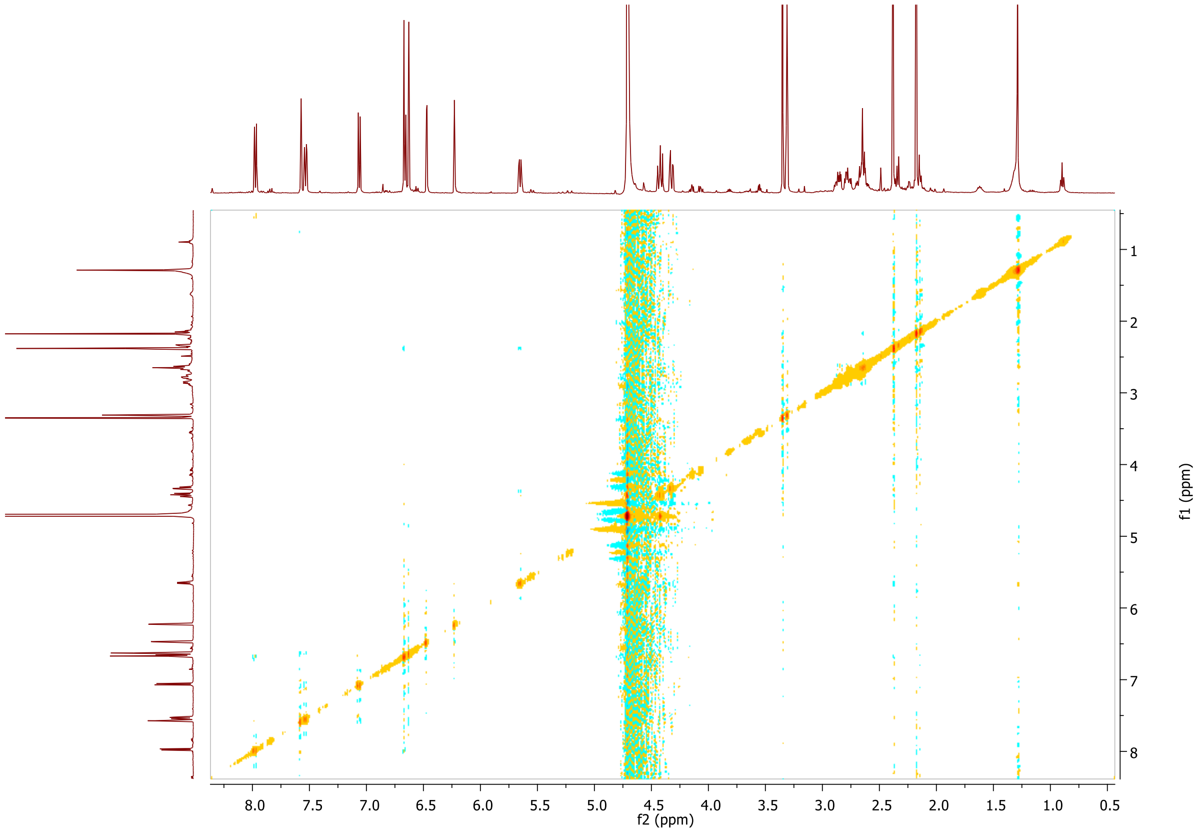

Figure S6. NOESY spectrum of compound **1** (in CD_3OD).

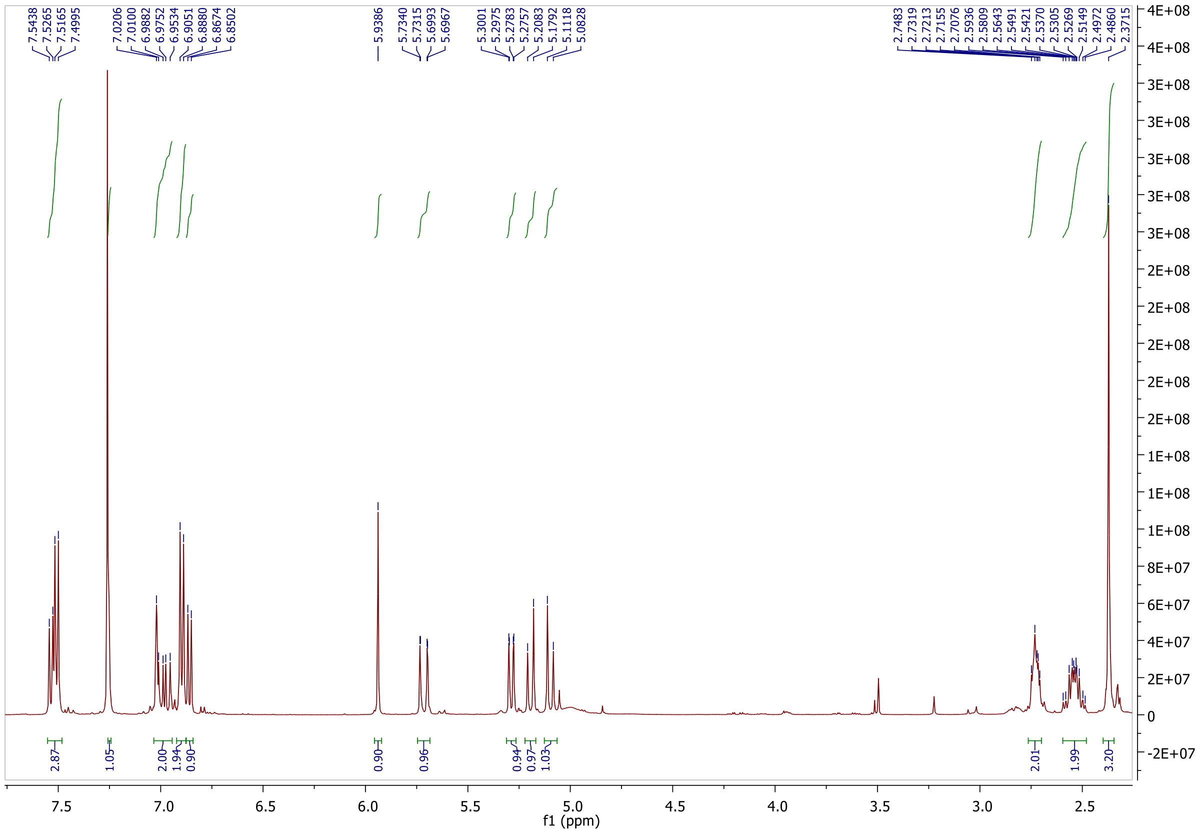

Figure S7. ^{1}H NMR spectrum of compound **2** (in $CDCl_3$).

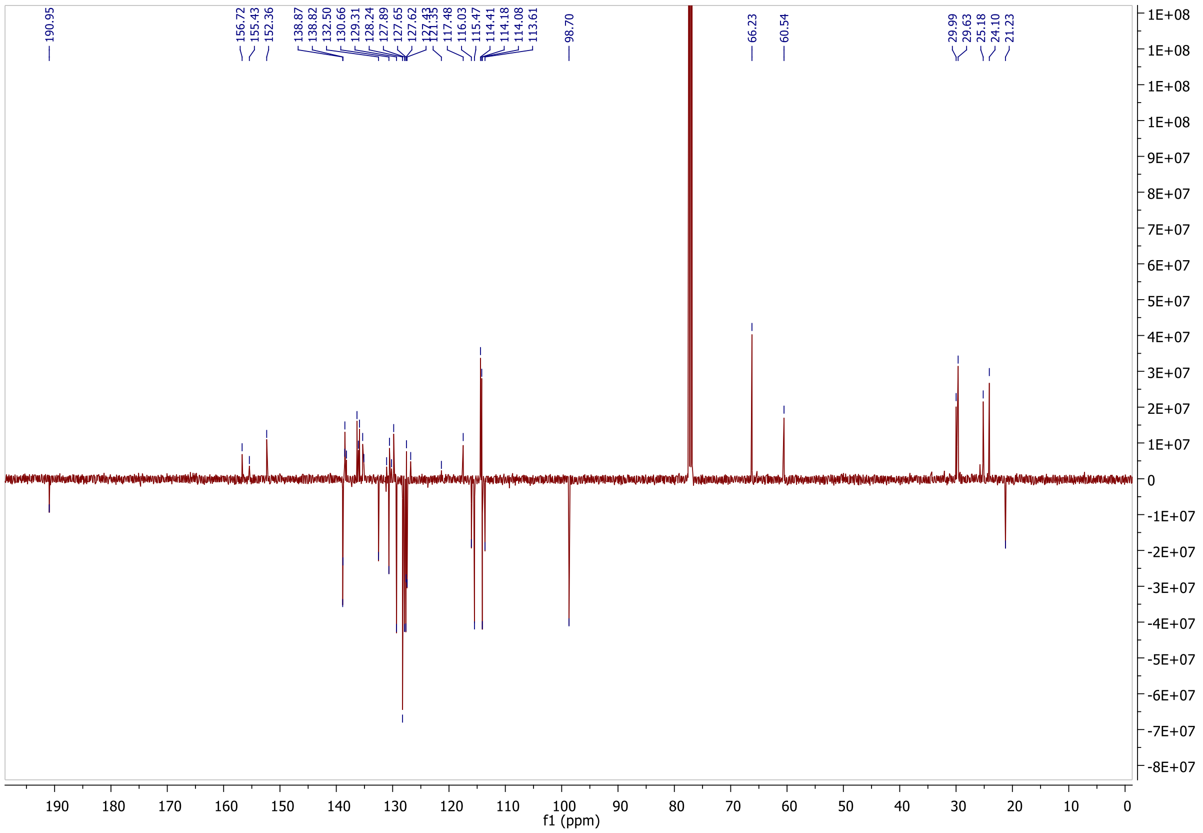

Figure S8. ^{13}C (JMOD) NMR spectrum of compound **2** (in $CDCl_3$).

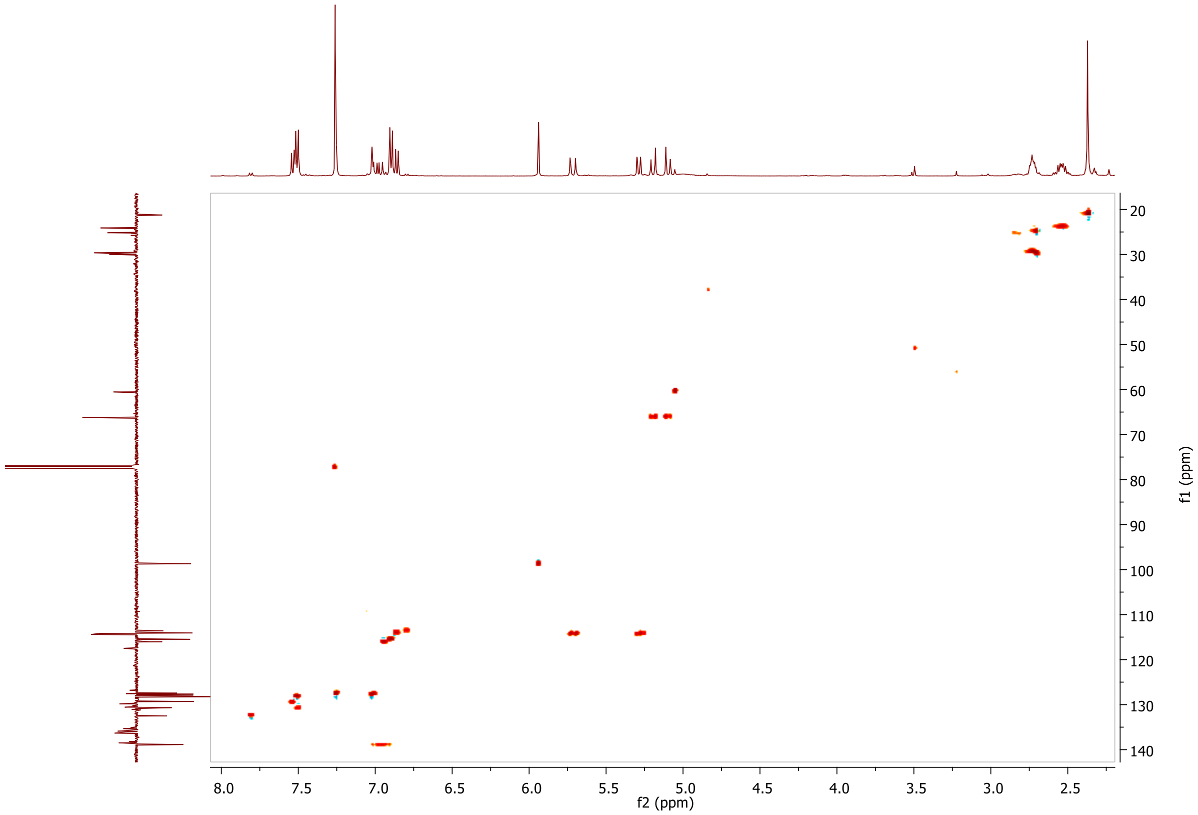

Figure S9. HSQC spectrum of compound **2** (in $CDCl_3$).

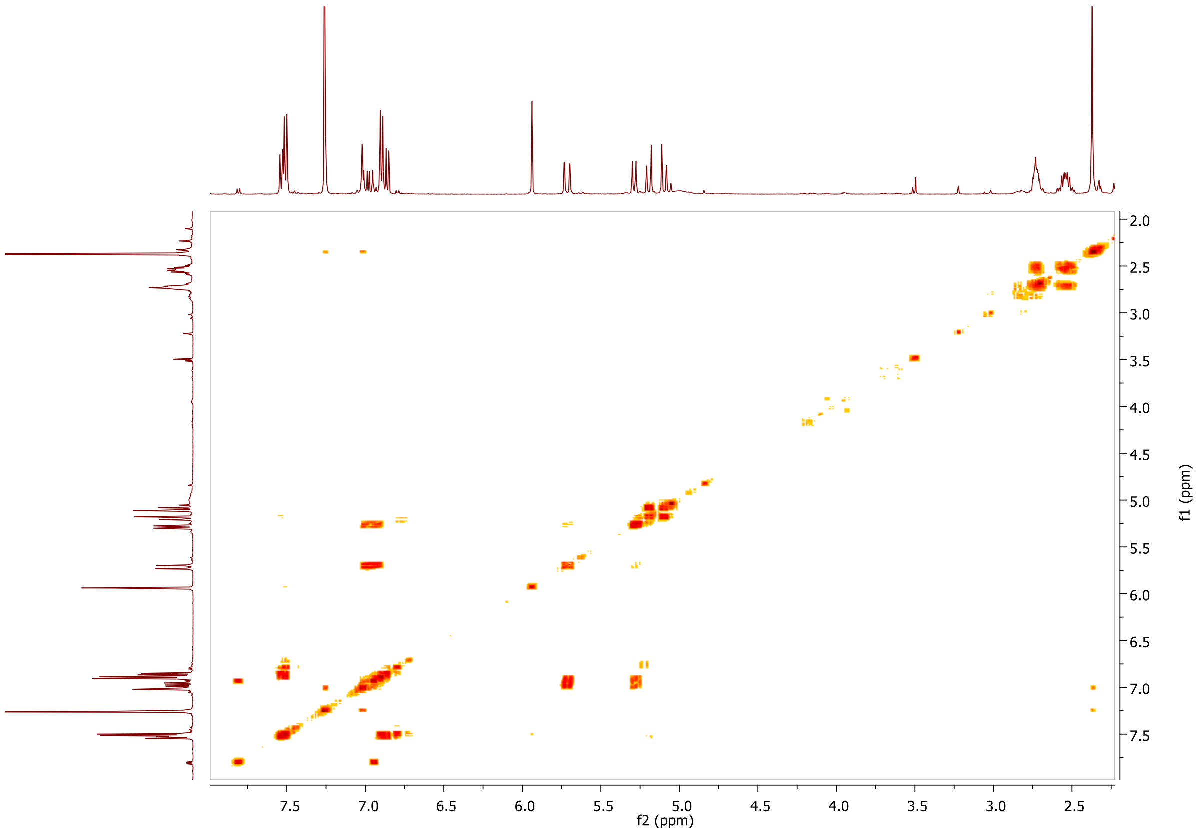

Figure S10. ^{1}H-^{1}H COSY spectrum of compound **2** (in $CDCl_3$).

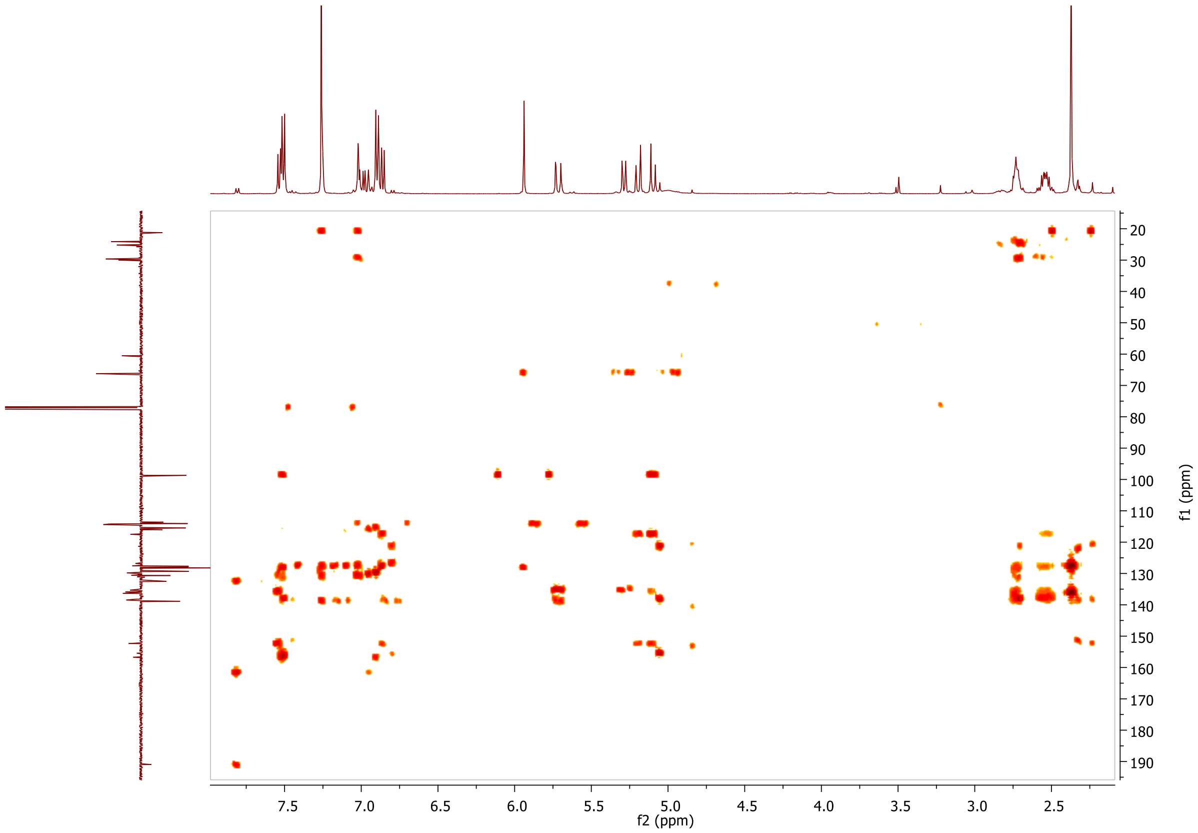

Figure S11. HMBC spectrum of compound **2** (in $CDCl_3$).

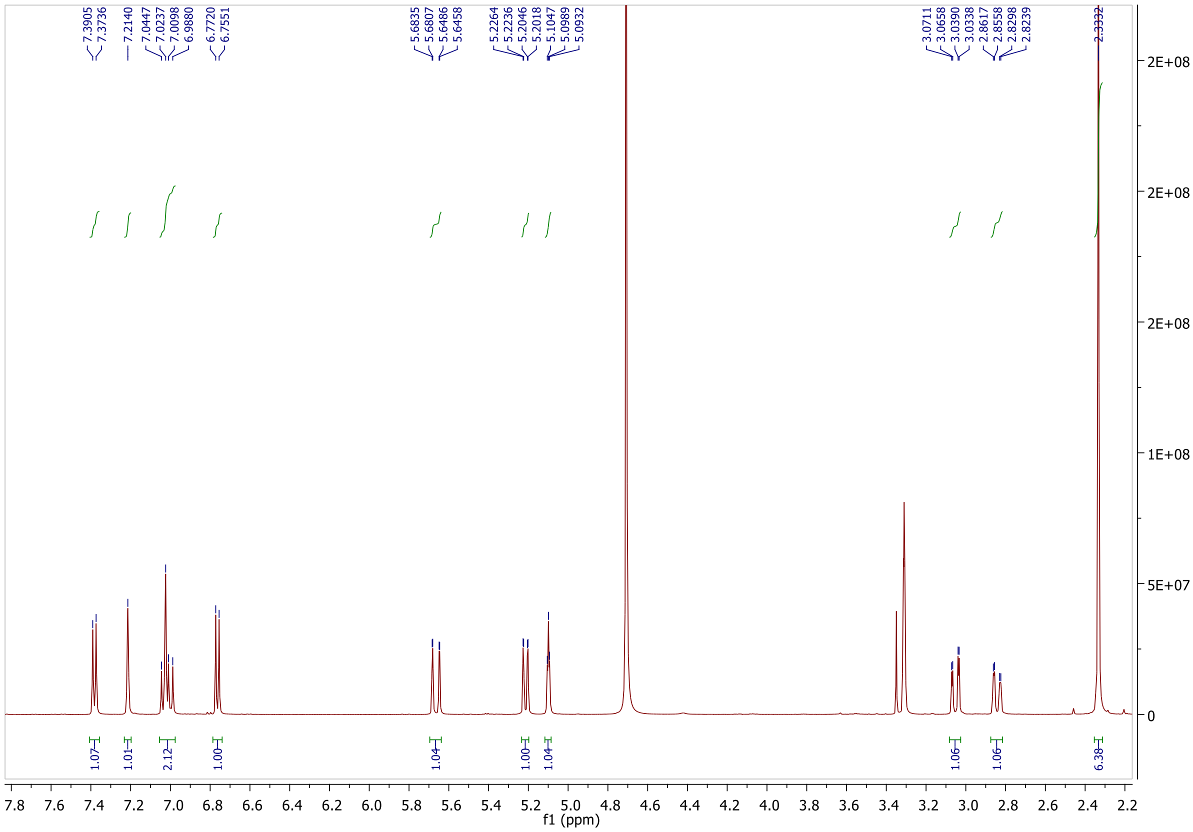

Figure S12. ^{1}H NMR spectrum of compound **3** (in CD_3OD).

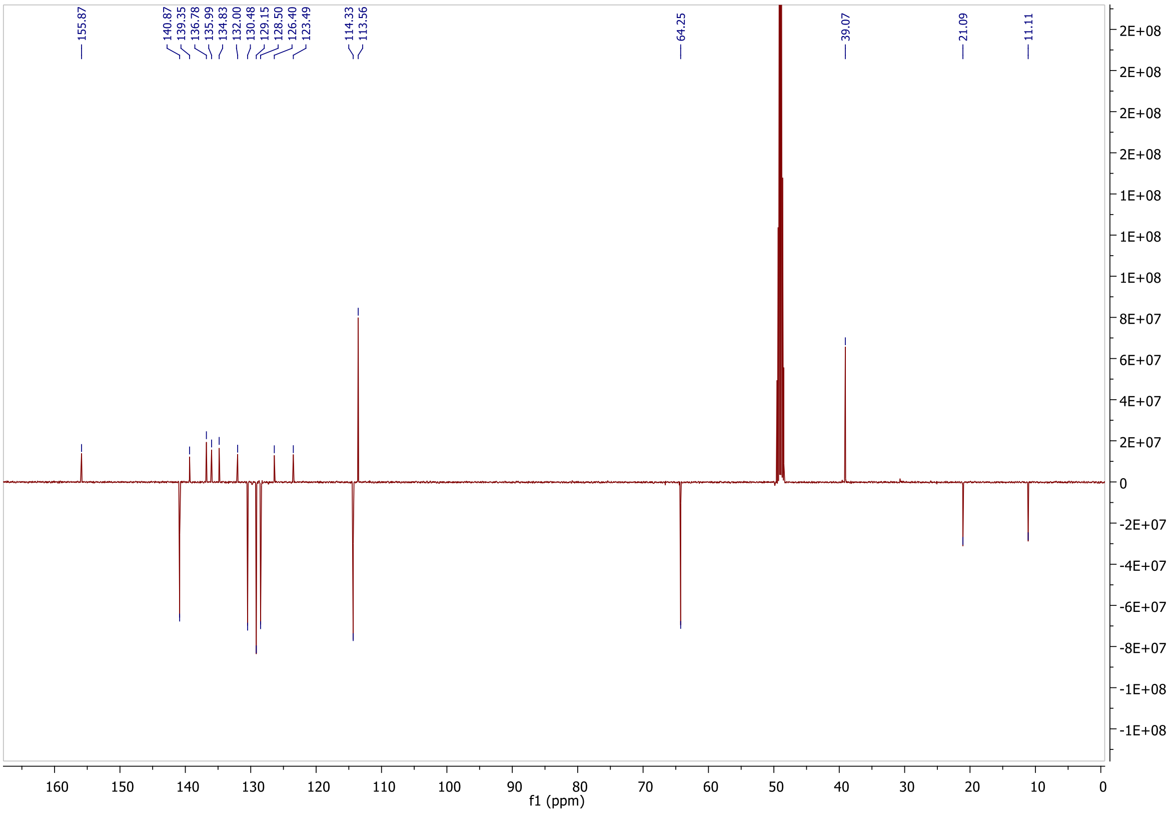

Figure S13. ^{13}C (JMOD) NMR spectrum of compound **3** (in CD_3OD).

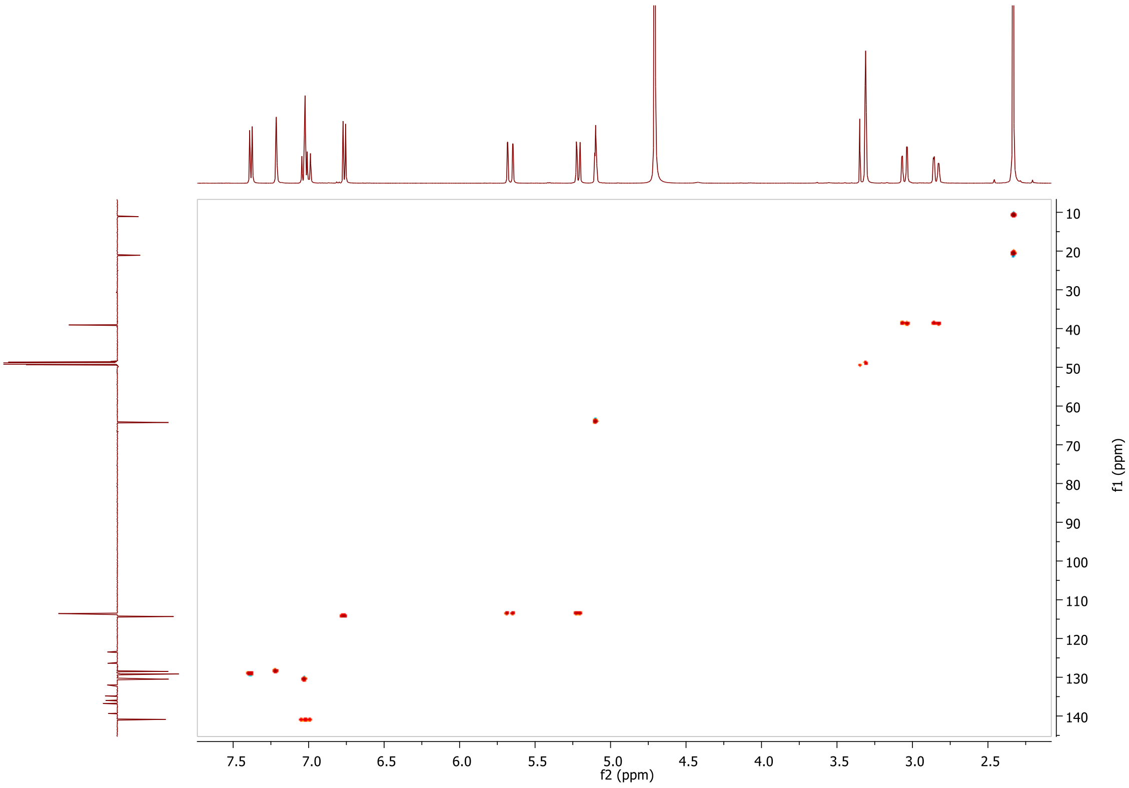

Figure S14. HSQC spectrum of compound **3** (in CD_3OD).

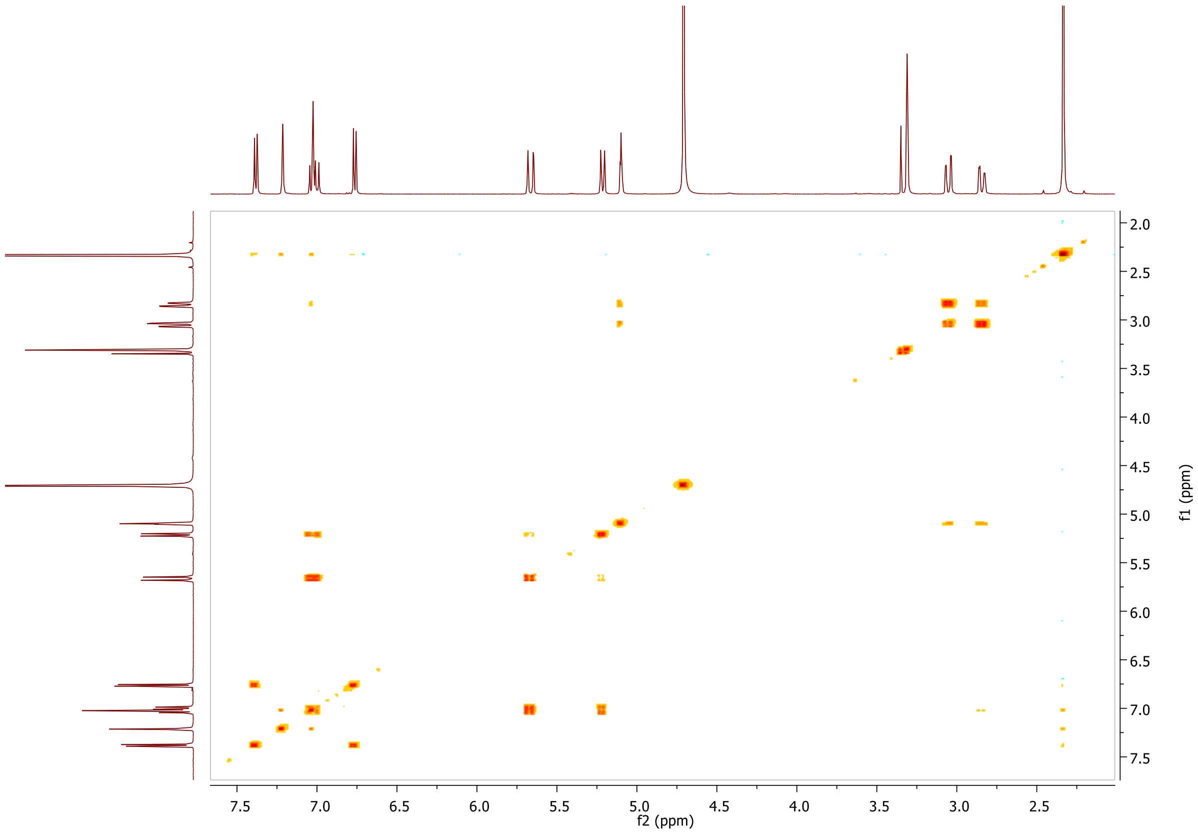

Figure S15. ^1H-^1H COSY spectrum of compound **3** (in CD_3OD).

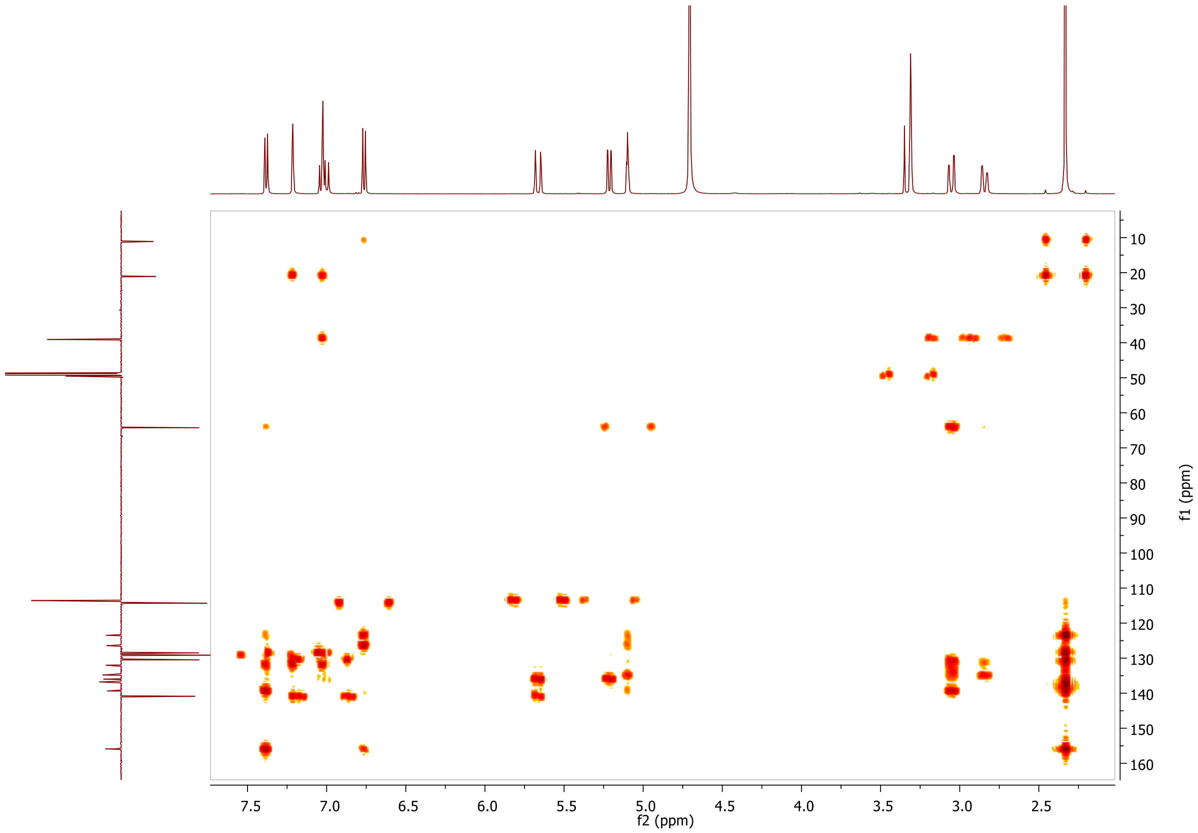

Figure S16. HMBC spectrum of compound **3** (in CD_3OD).

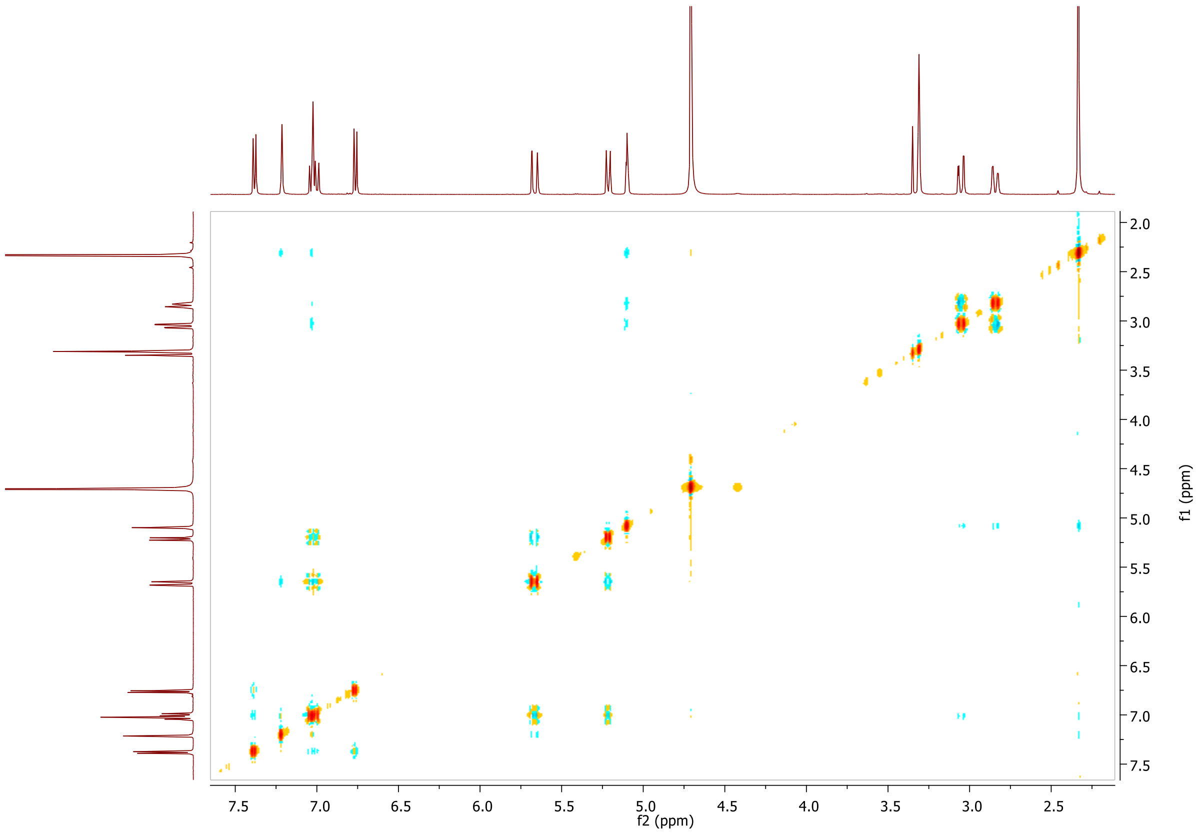

Figure S17. NOESY spectrum of compound **3** (in CD_3OD).

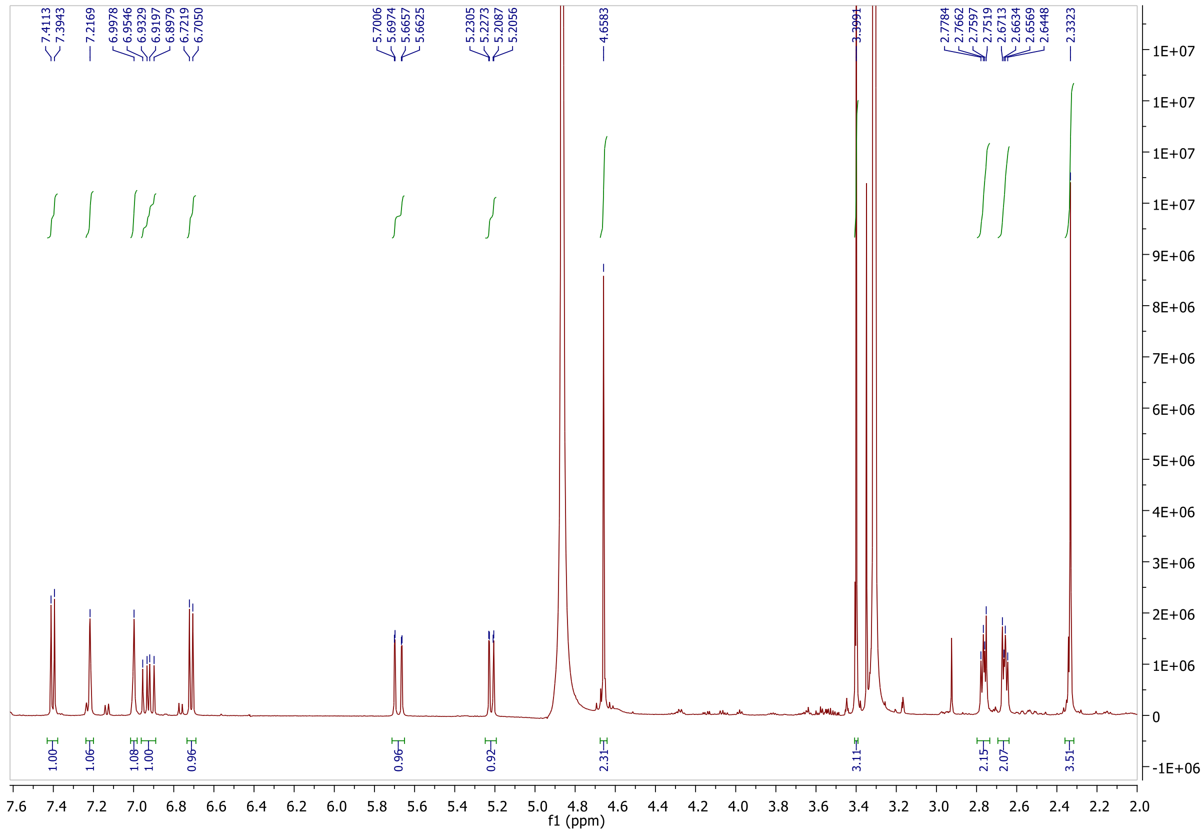

Figure S18. ^{1}H NMR spectrum of compound **4** (in CD_3OD).

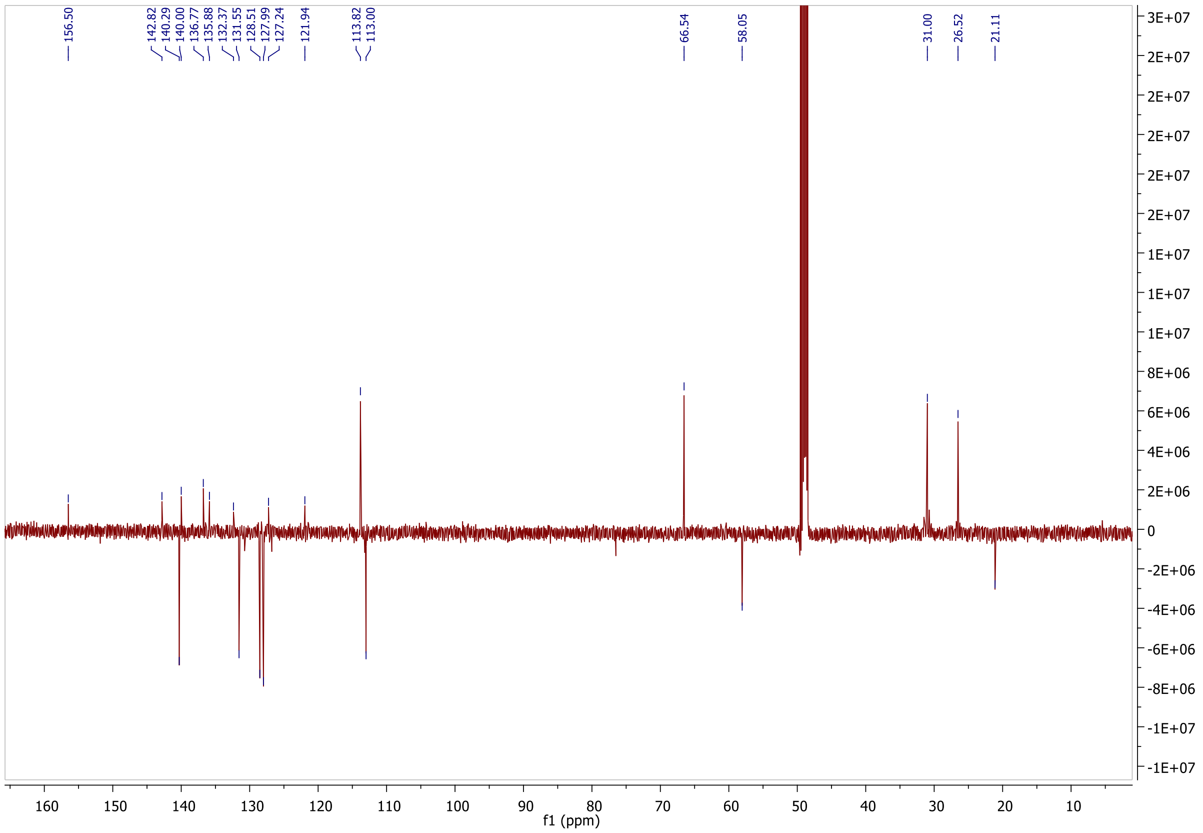

Figure S19. ^{13}C (JMOD) NMR spectrum of compound **4** (in CD_3OD).

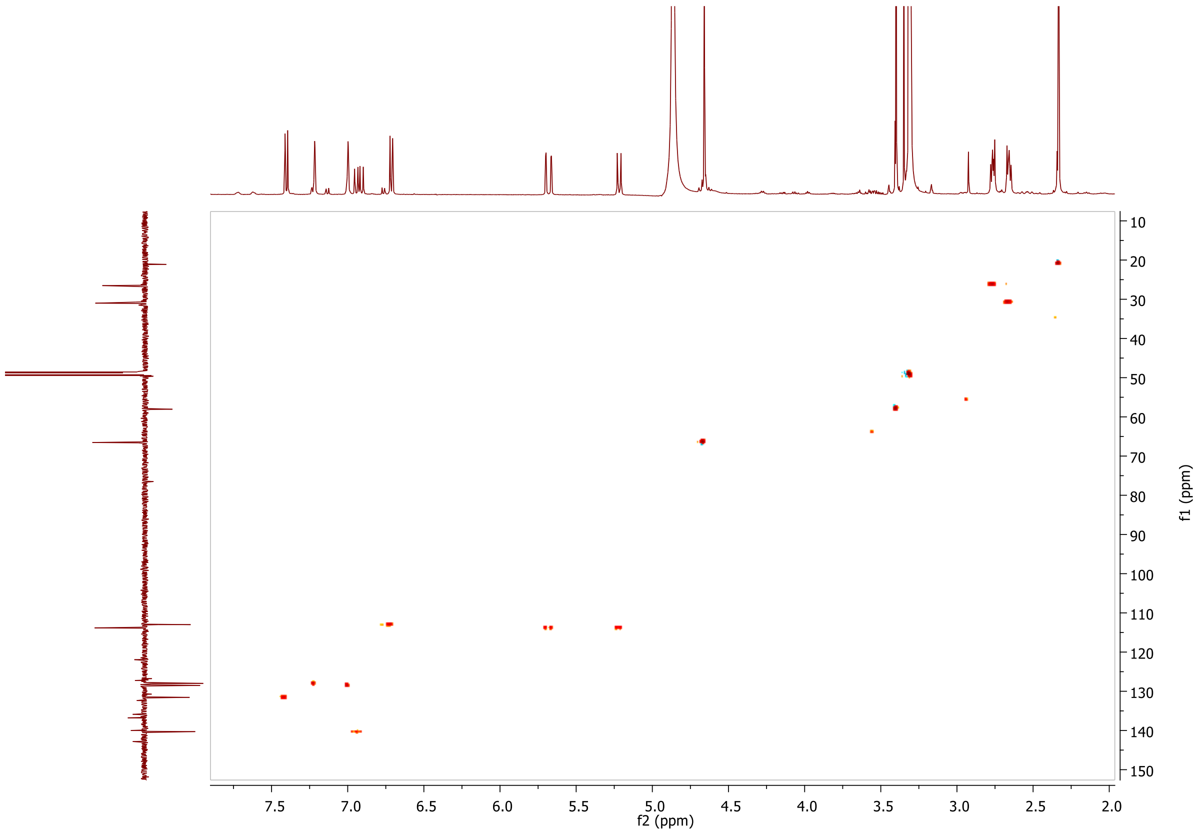

Figure S20. HSQC spectrum of compound **4** (in CD_3OD).

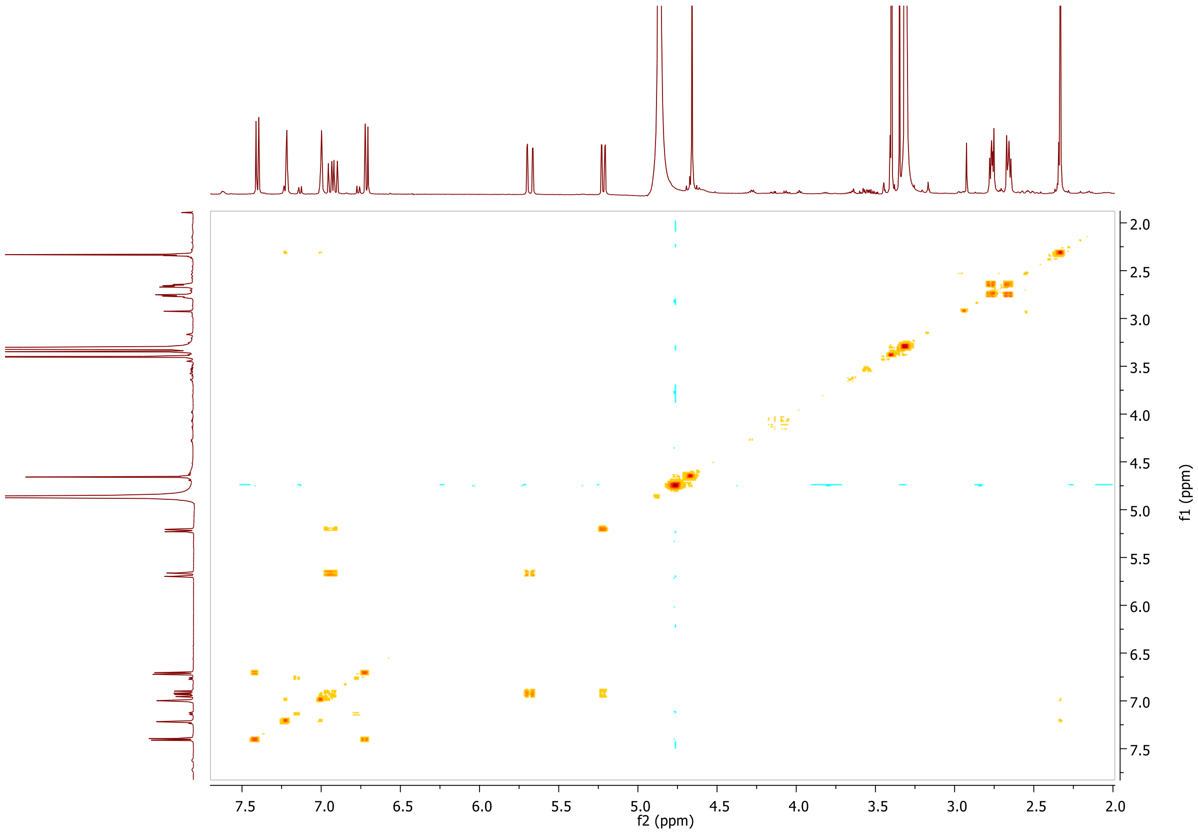

Figure S21. ^1H-^1H COSY spectrum of compound **4** (in CD_3OD).

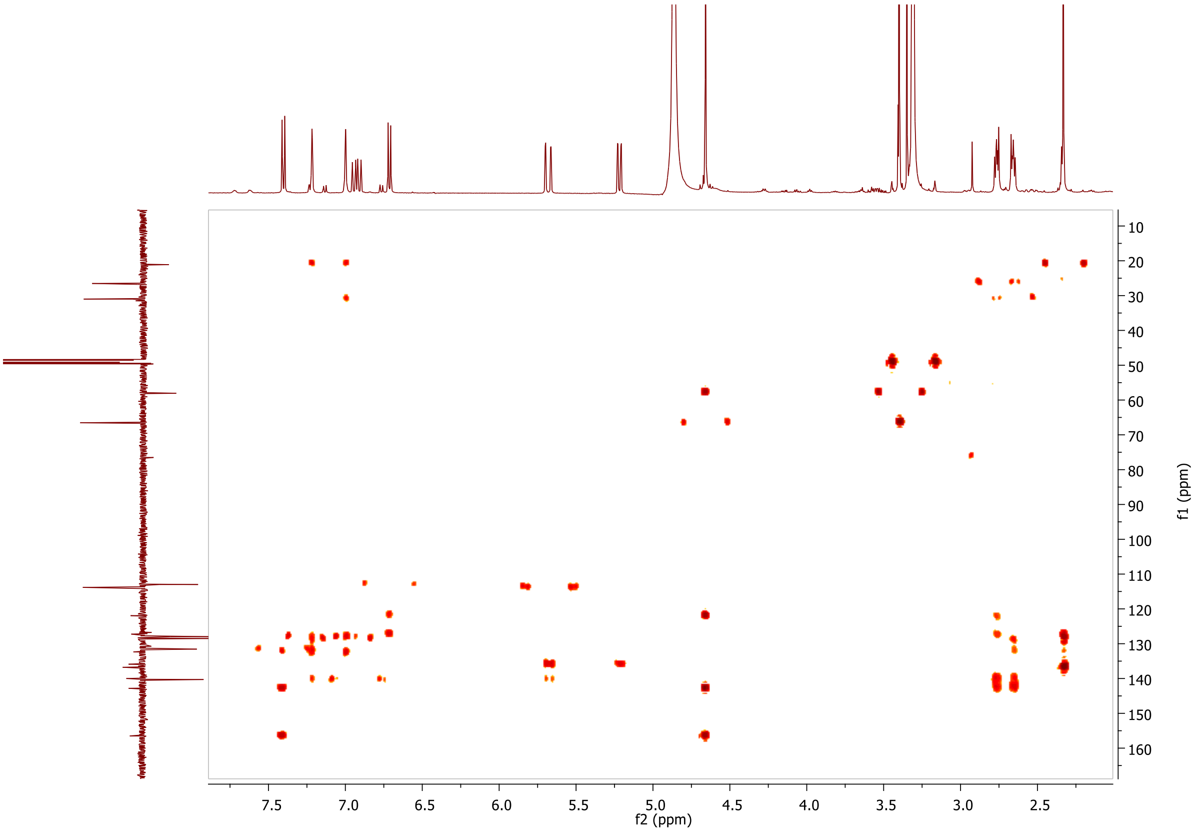

Figure S22. HMBC spectrum of compound **4** (in CD_3OD).

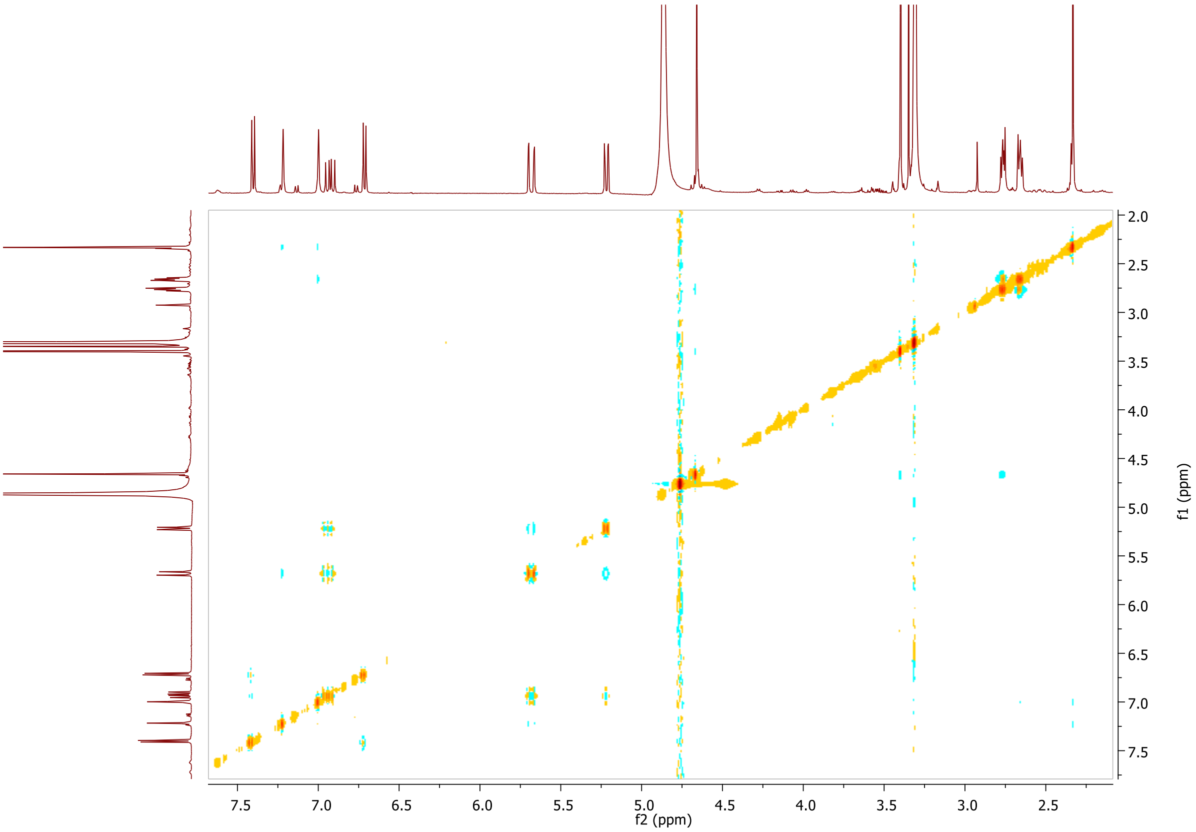

Figure S23. NOESY spectrum of compound **4** (in CD_3OD).

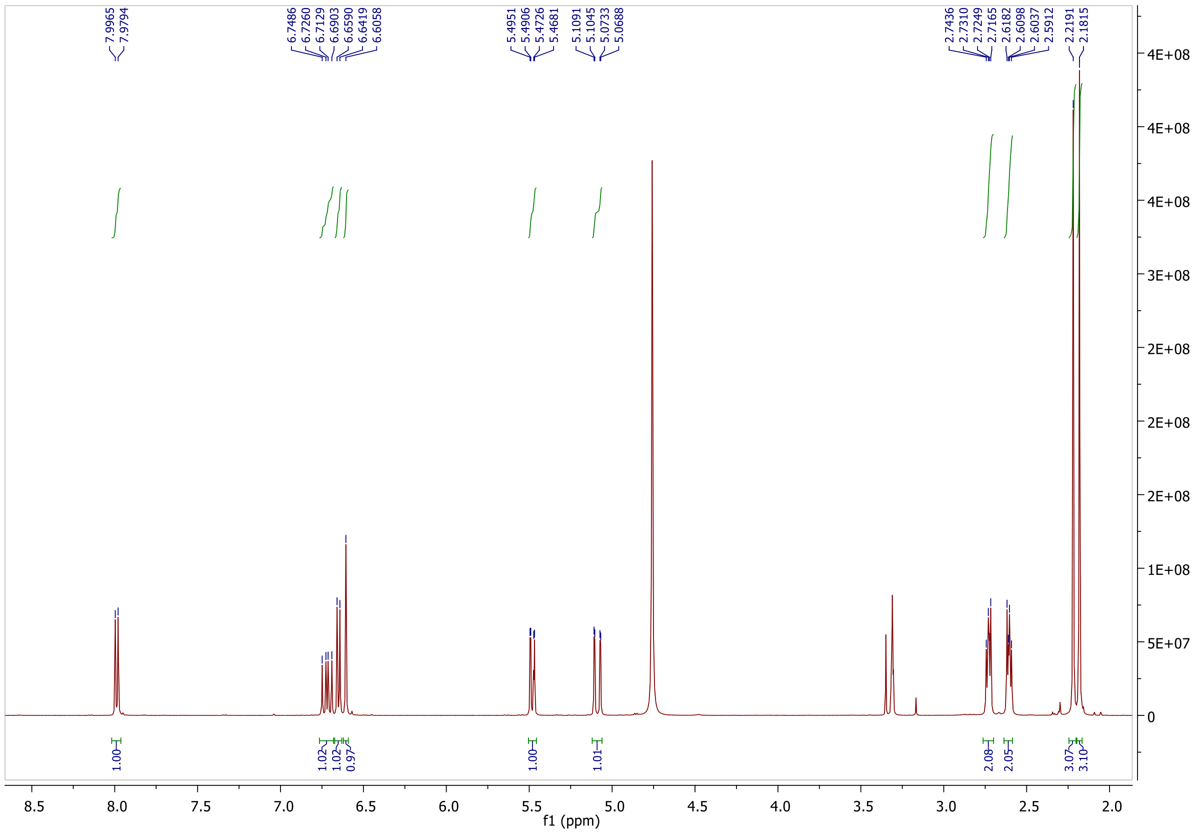

Figure S24. ^1H NMR spectrum of compound **5** (in CD_3OD).

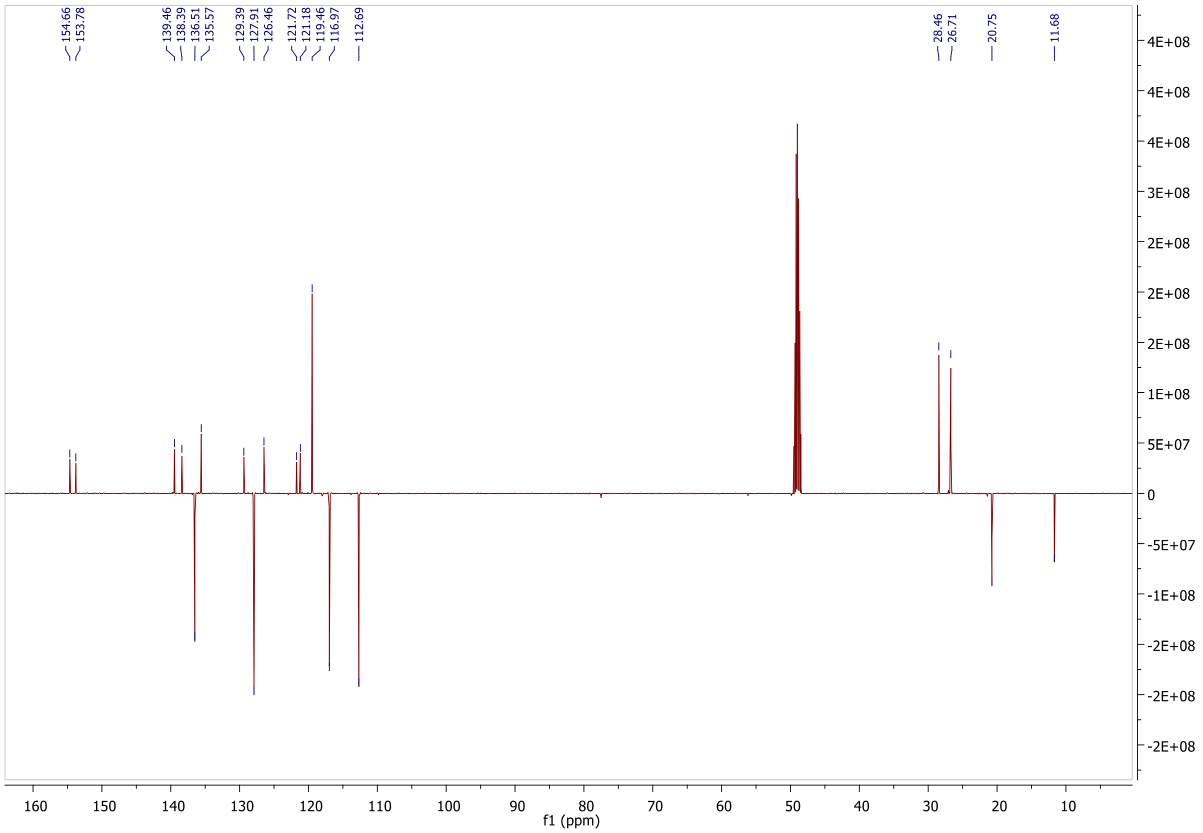

Figure S25. ^{13}C (JMOD) NMR spectrum of compound **5** (in CD3OD).

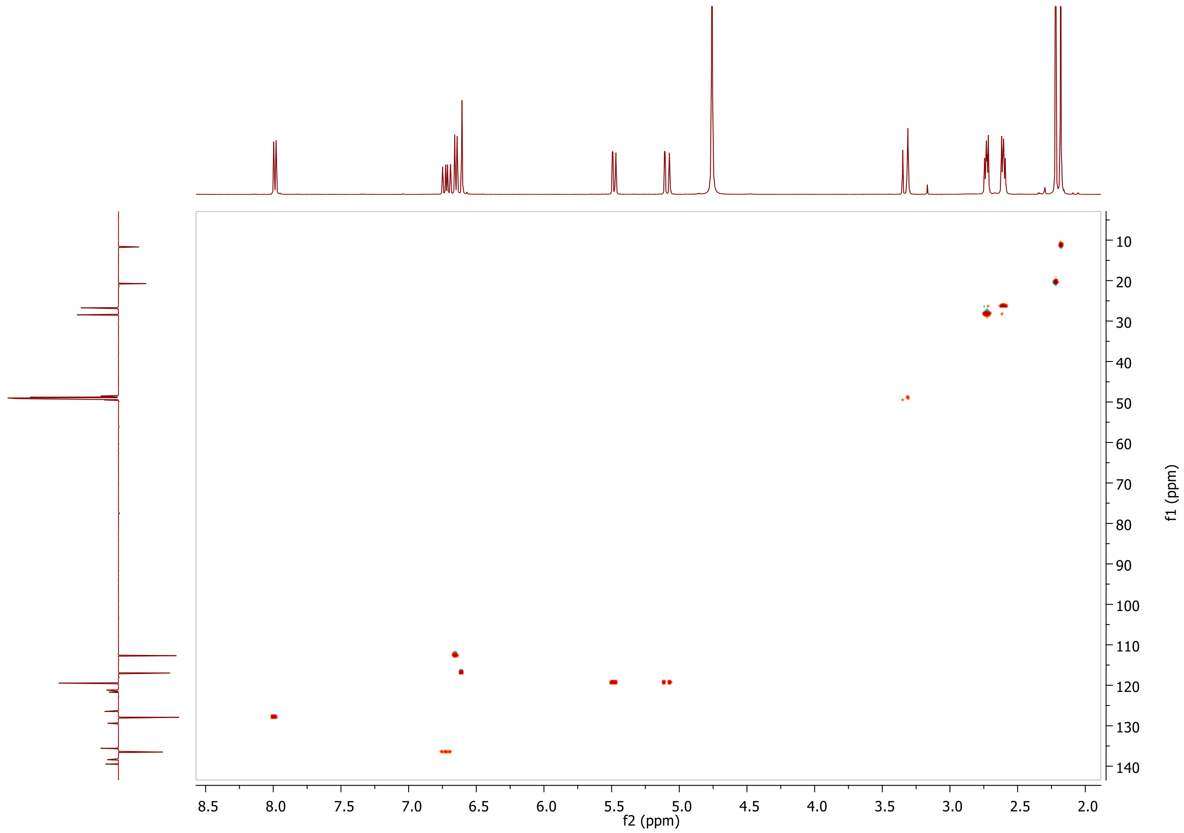

Figure S25. HSQC spectrum of compound **5** (in CD_3OD).

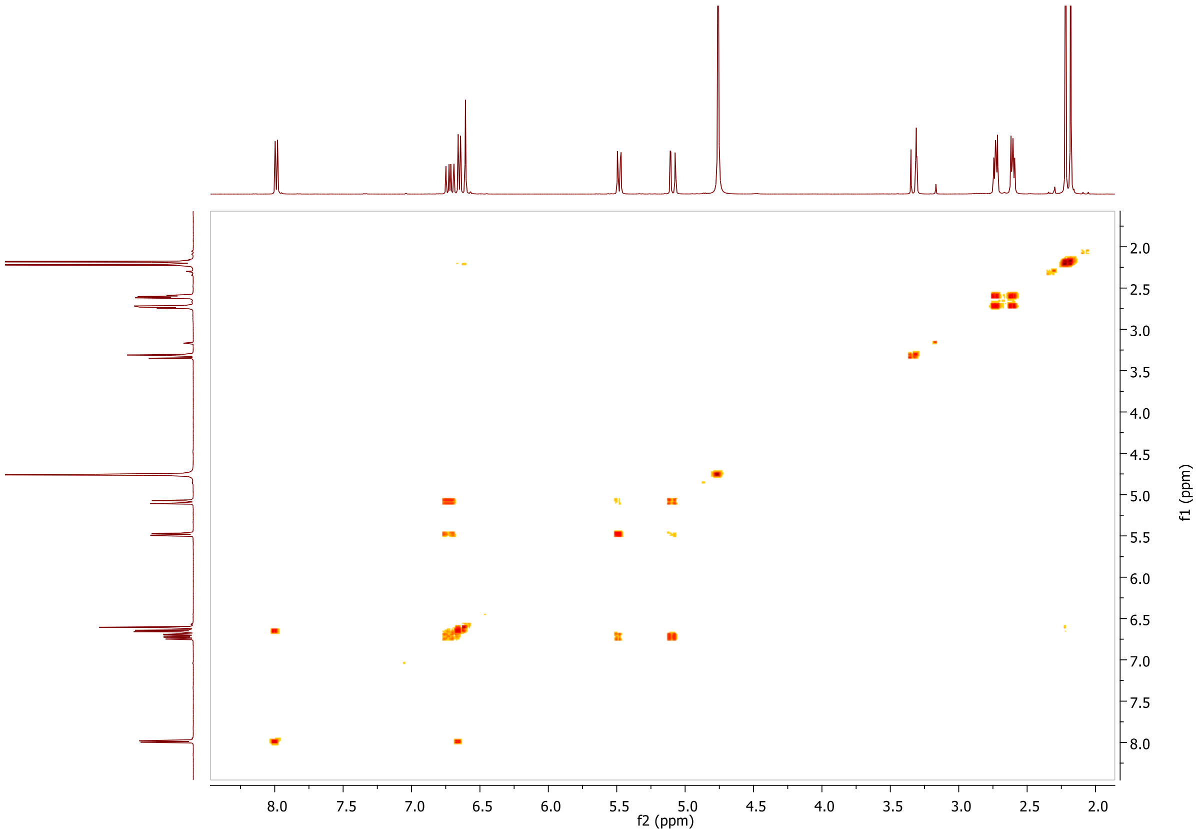

Figure S26. ^{1}H-^{1}H COSY spectrum of compound **5** (in CD_3OD).

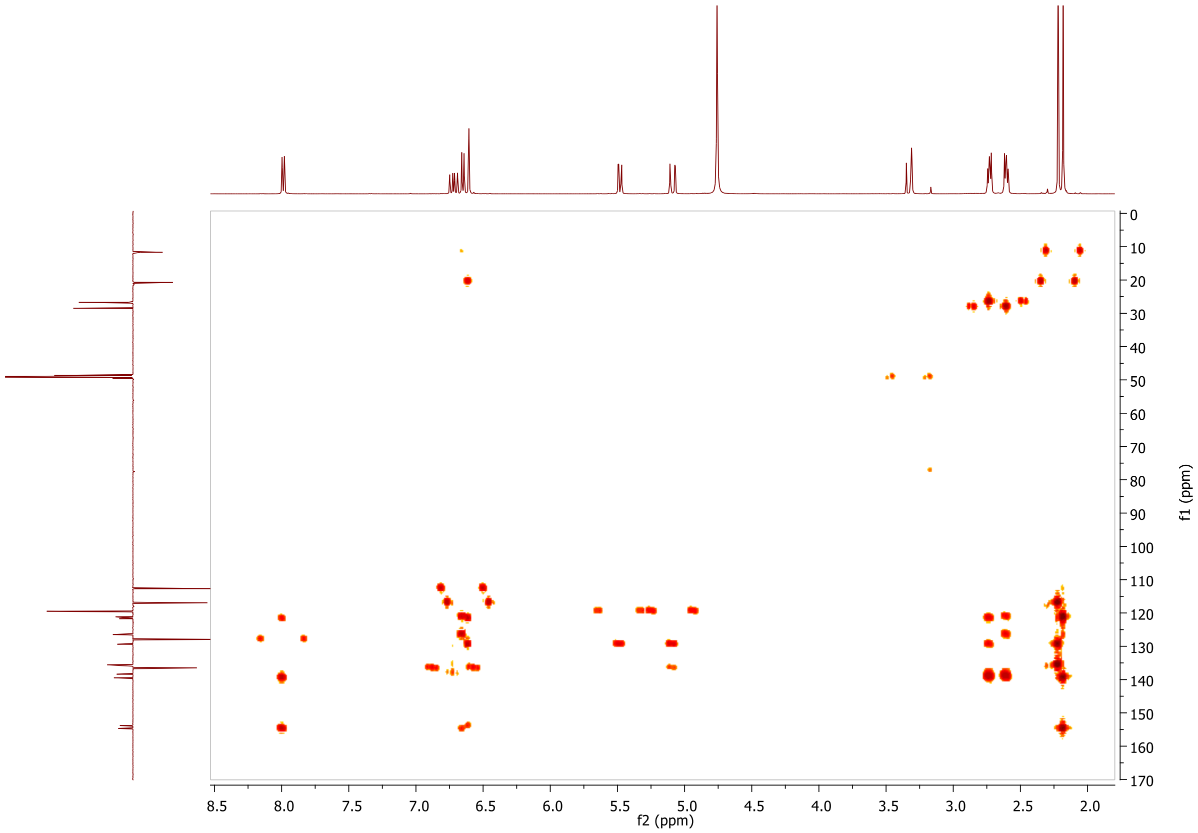

Figure S27. HMBC spectrum of compound **5** (in CD_3OD).

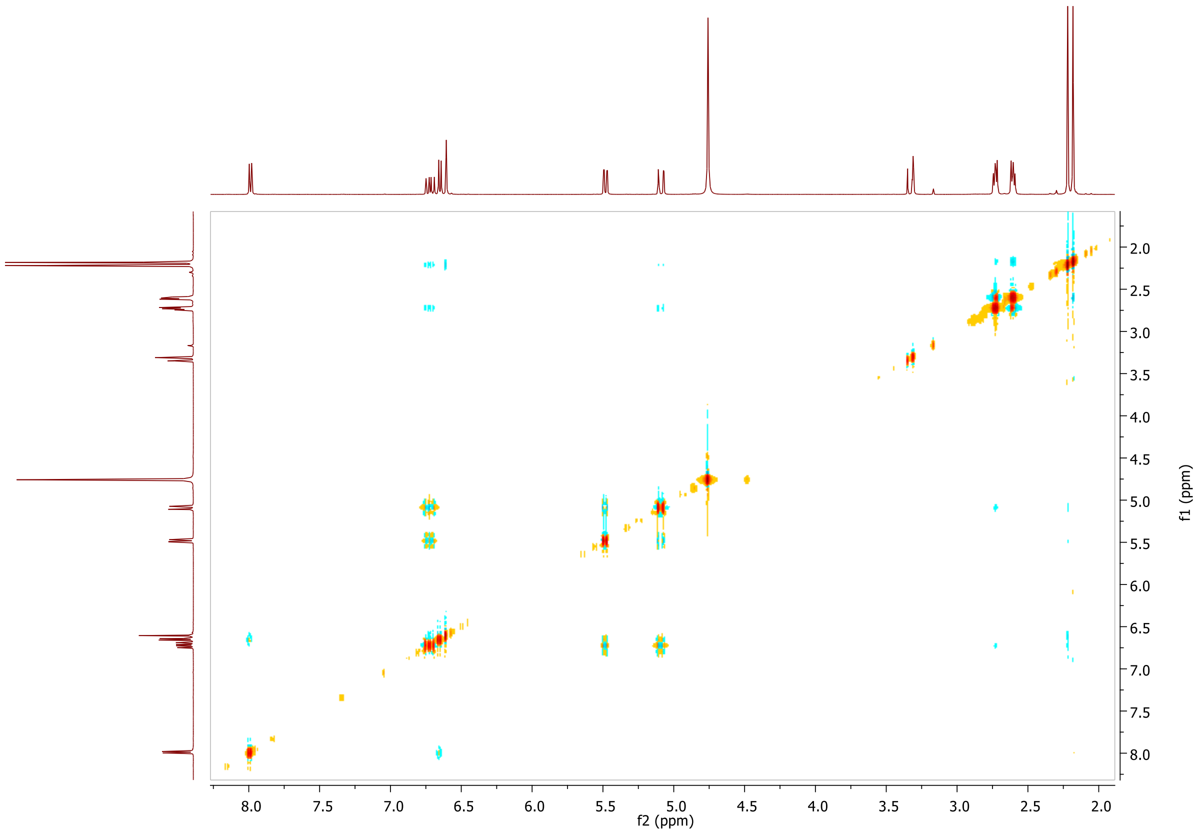

Figure S28. NOESY spectrum of compound **5** (in CD_3OD).

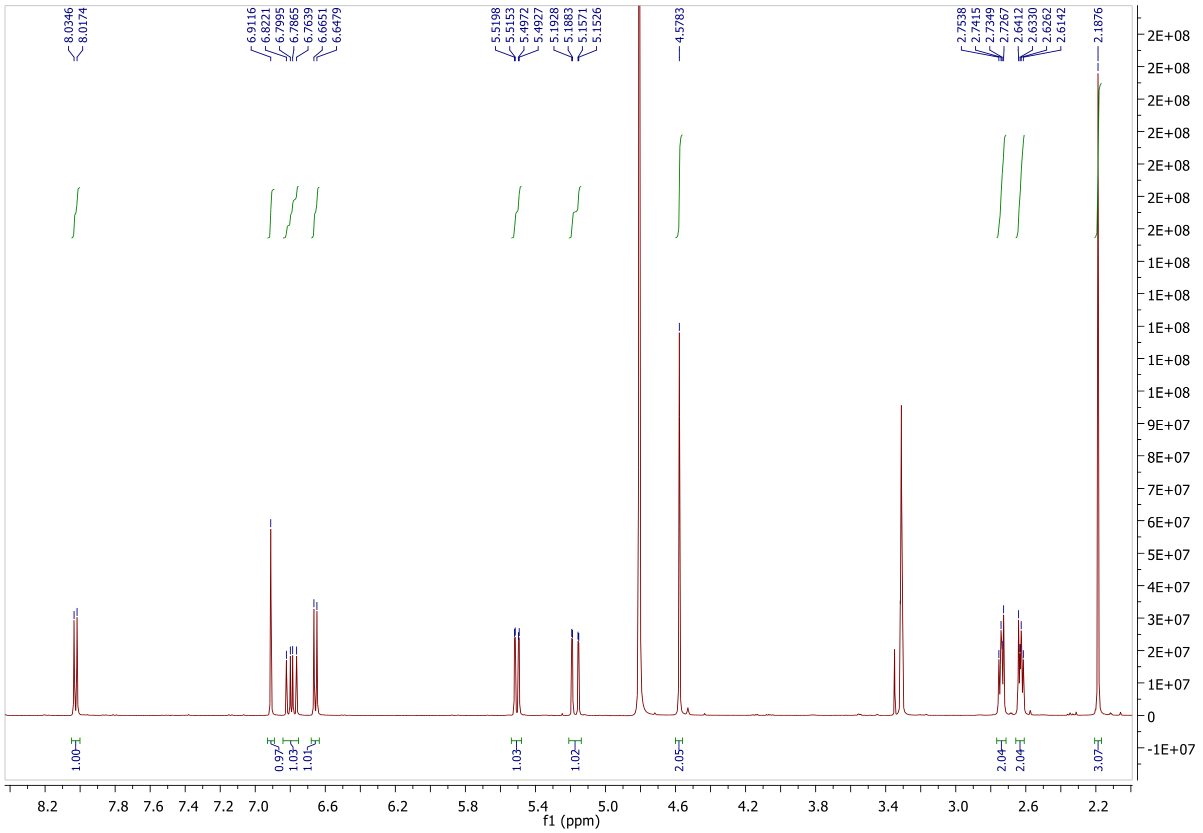

Figure S29. ^{1}H NMR spectrum of compound **6** (in CD_3OD).

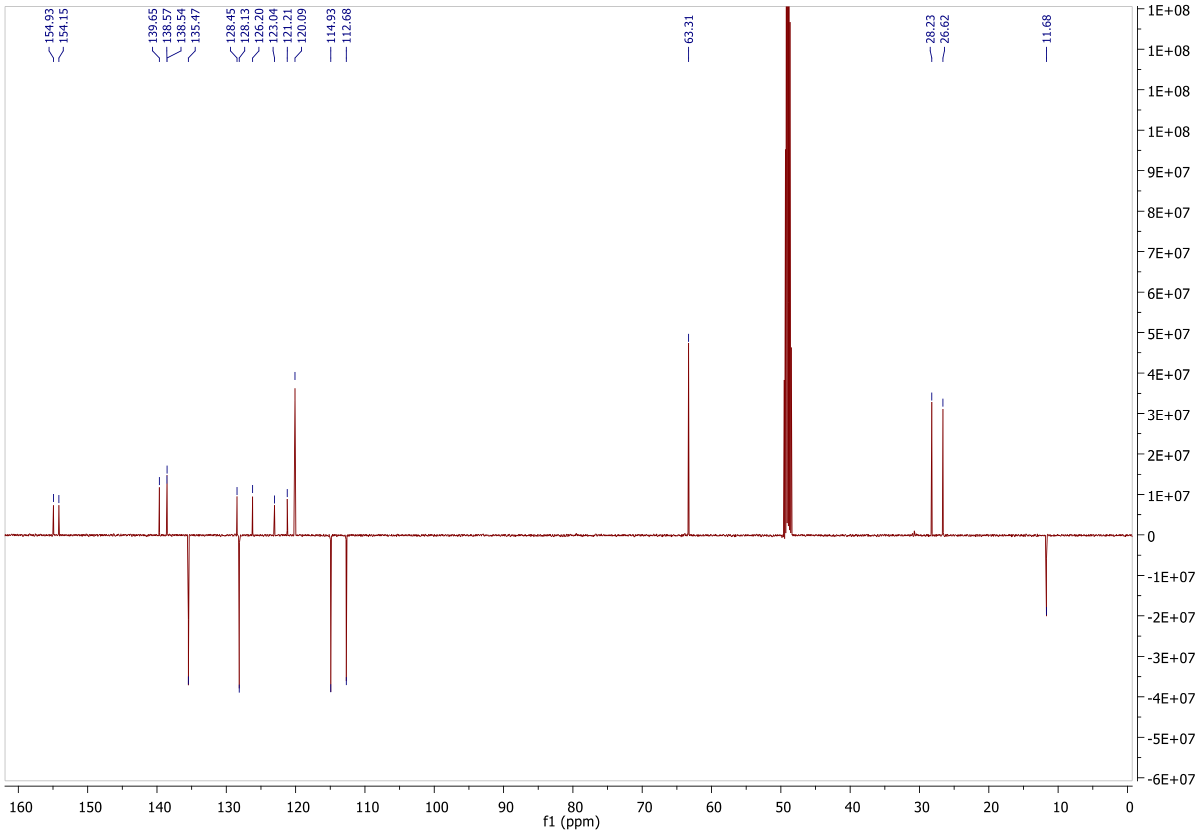

Figure S30. ^{13}C (JMOD) NMR spectrum of compound **6** (in CD_3OD).

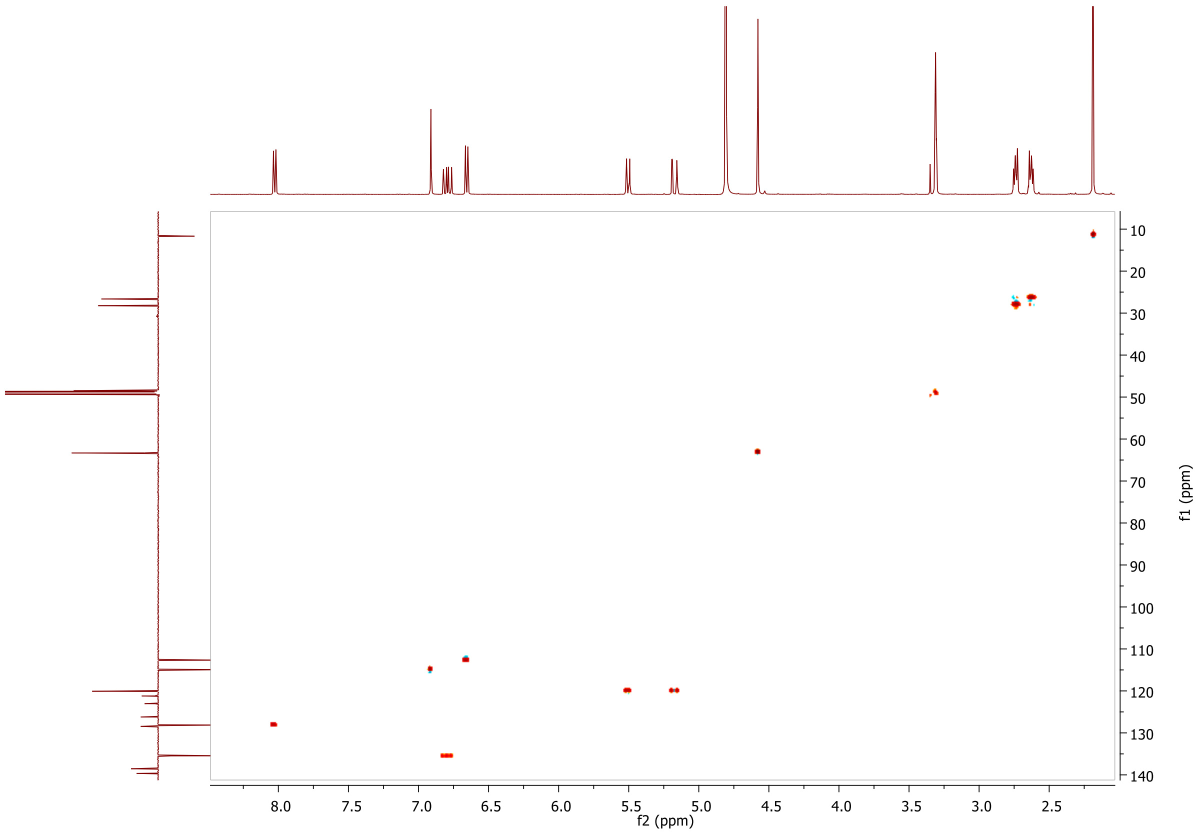

Figure S31. HSQC spectrum of compound **6** (in CD_3OD).

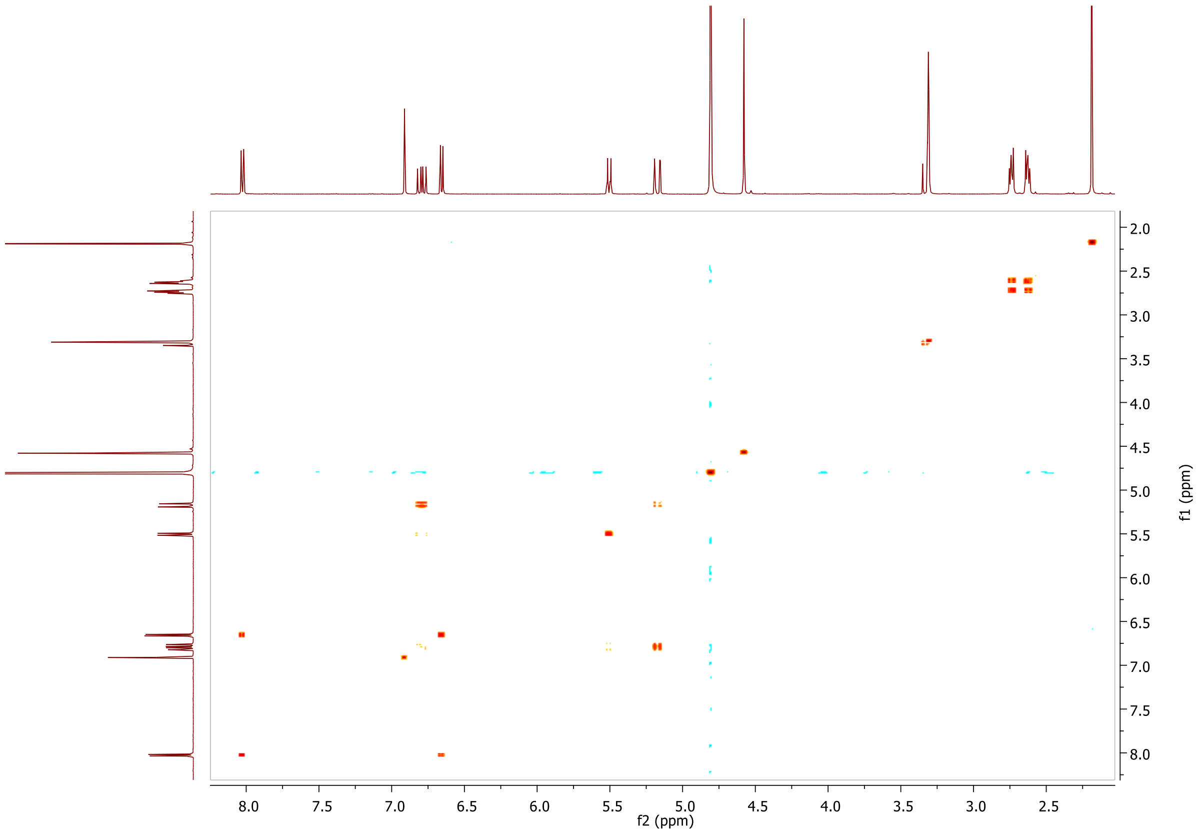

Figure S32. ^{1}H-^{1}H COSY spectrum of compound **6** (in CD_3OD).

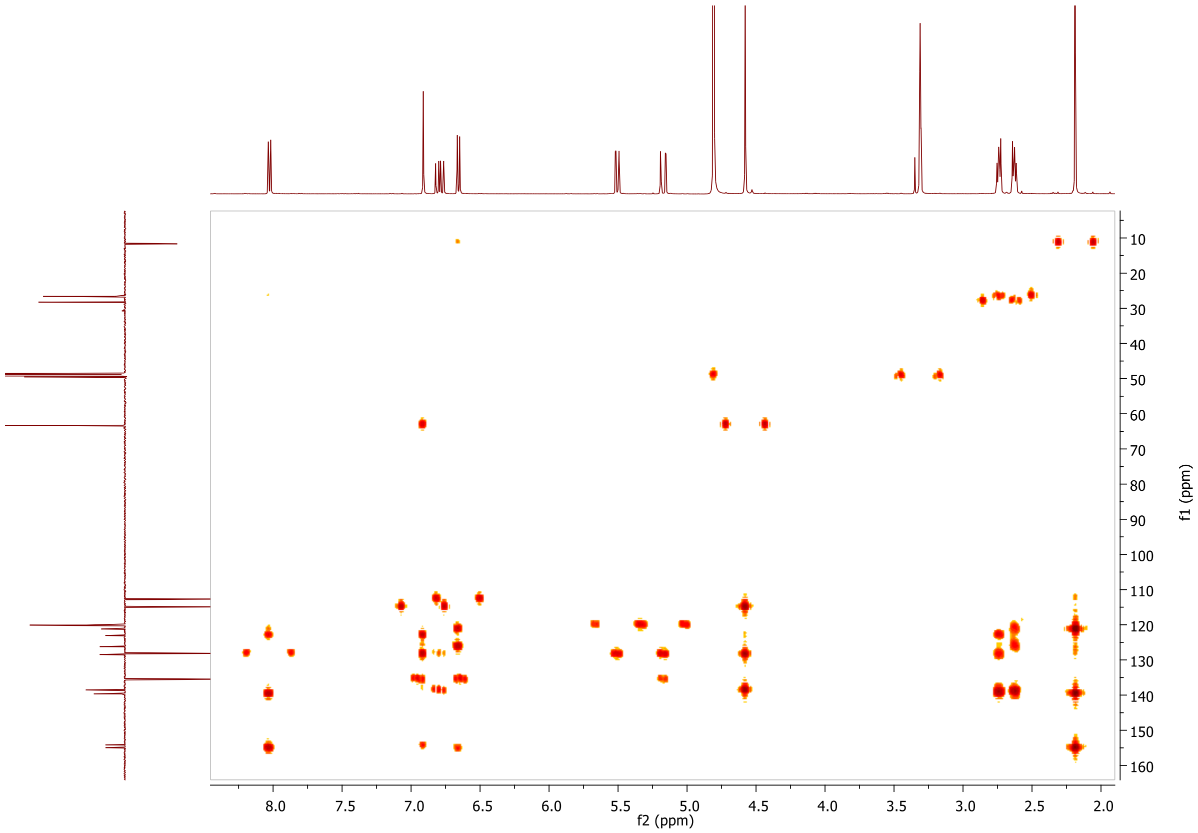

Figure S33. HMBC spectrum of compound **6** (in CD_3OD).

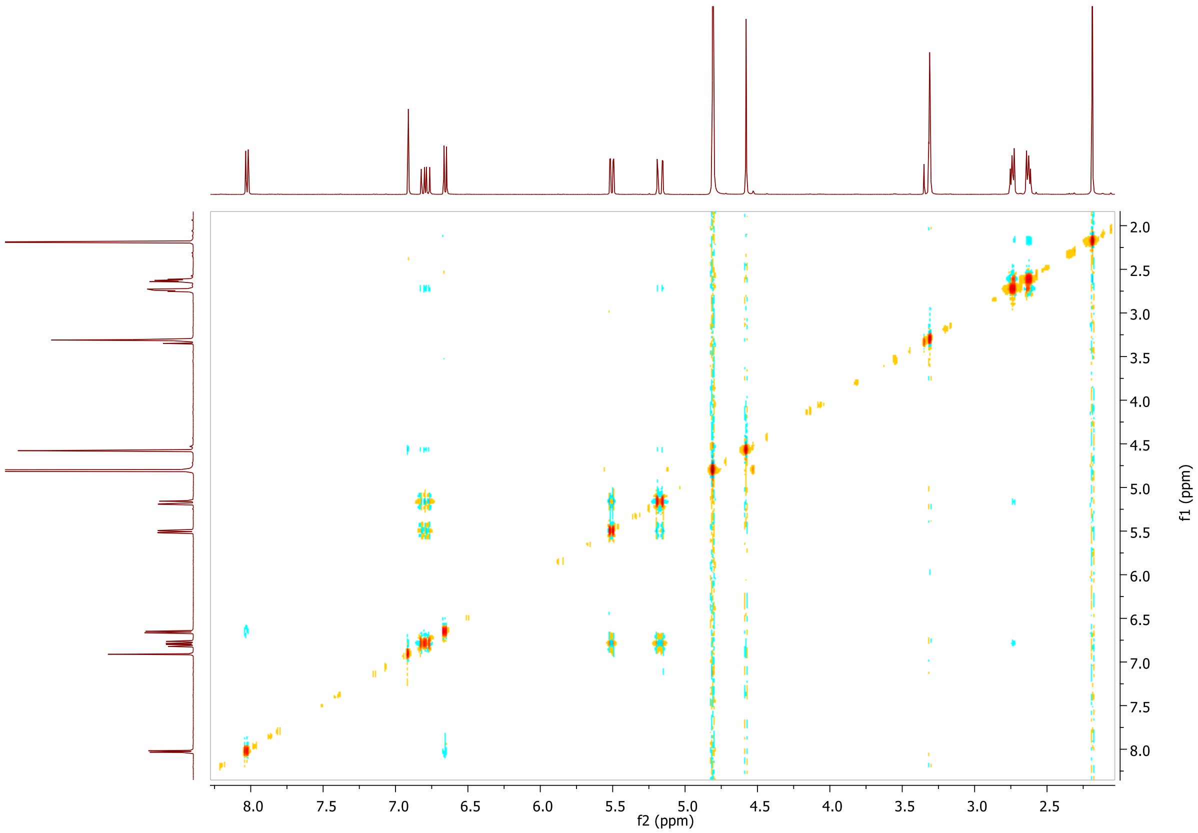

Figure S34. NOESY spectrum of compound **6** (in CD_3OD).

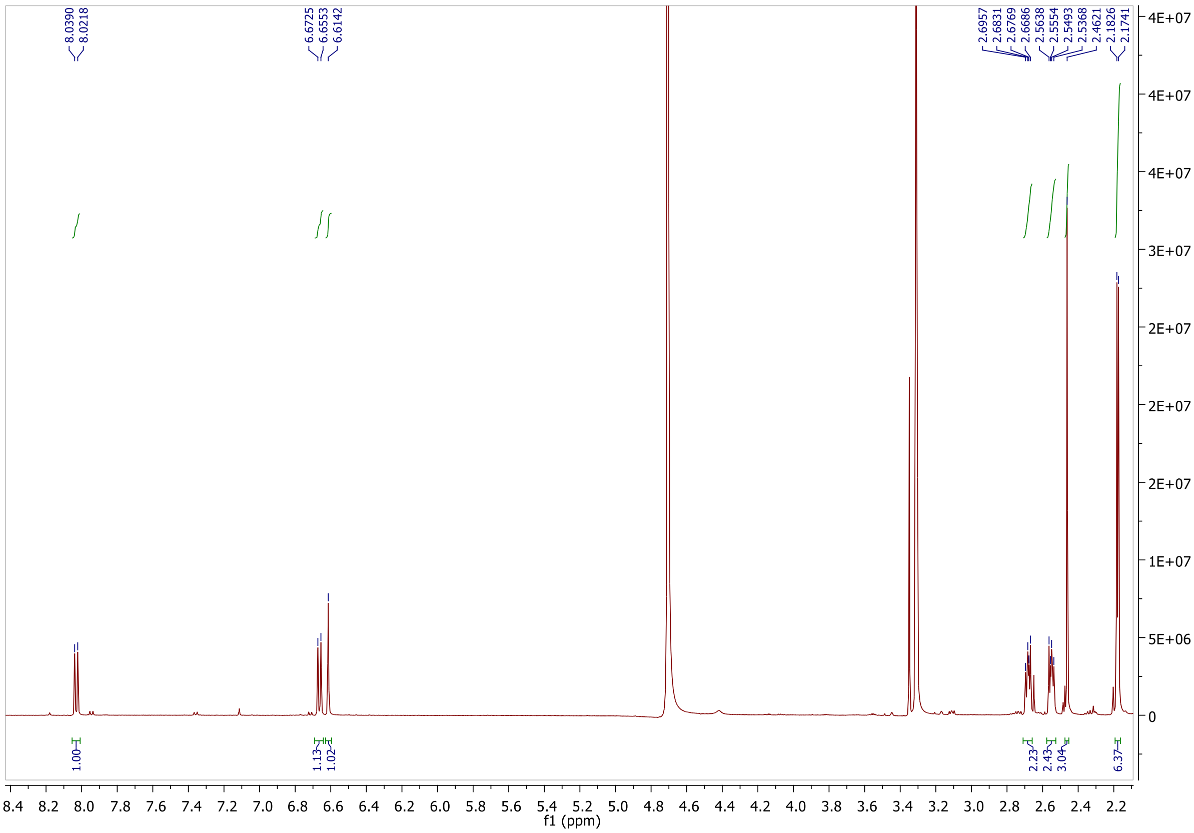

Figure S35. ^{1}H NMR spectrum of compound **7** (in CD_3OD).

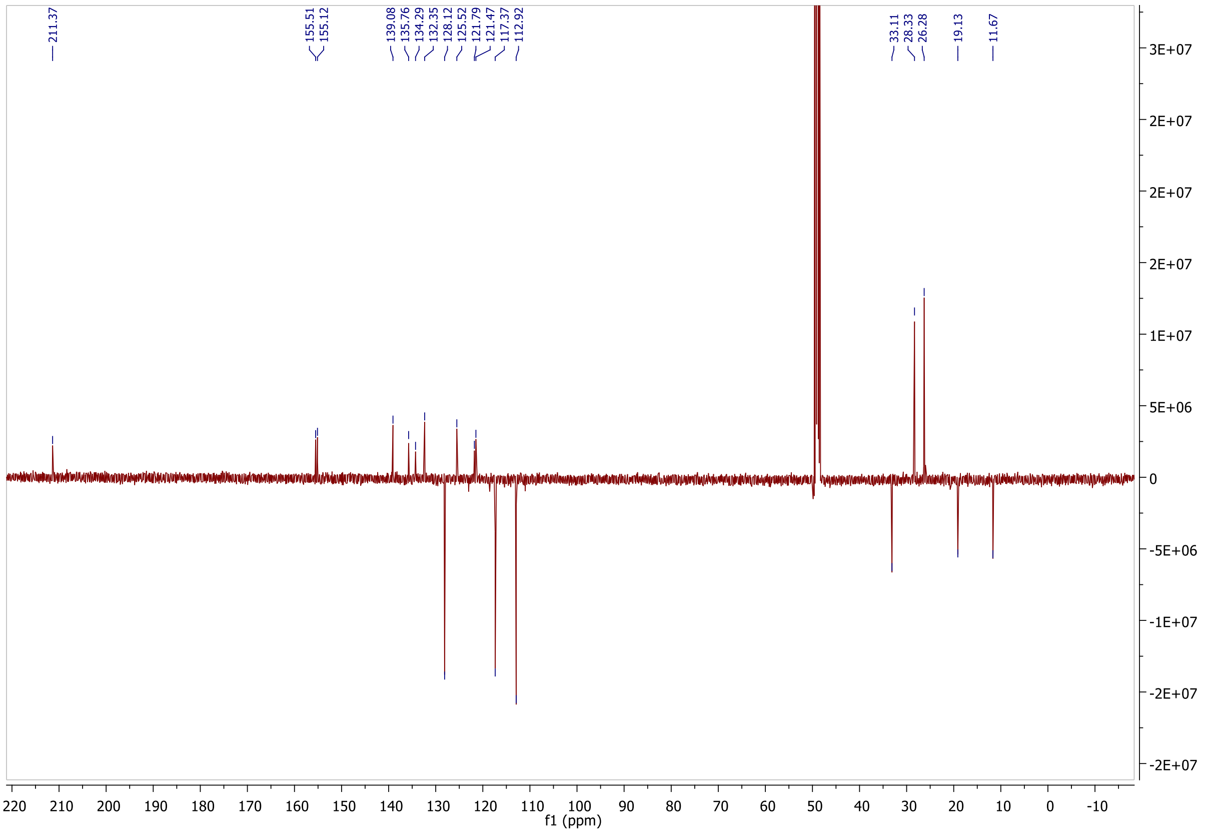

Figure S36. ^{13}C (JMOD) spectrum of compound **7** (in CD_3OD).

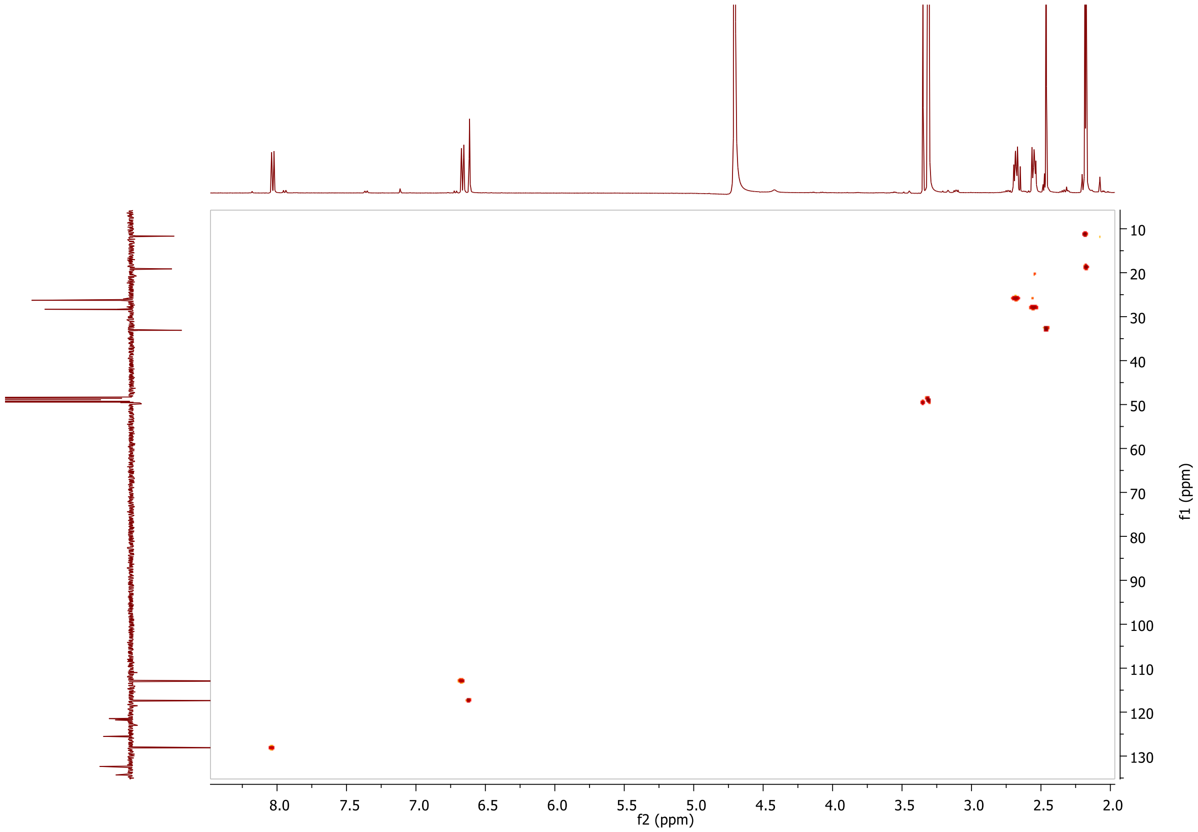

Figure S37. HSQC spectrum of compound **7** (in CD_3OD).

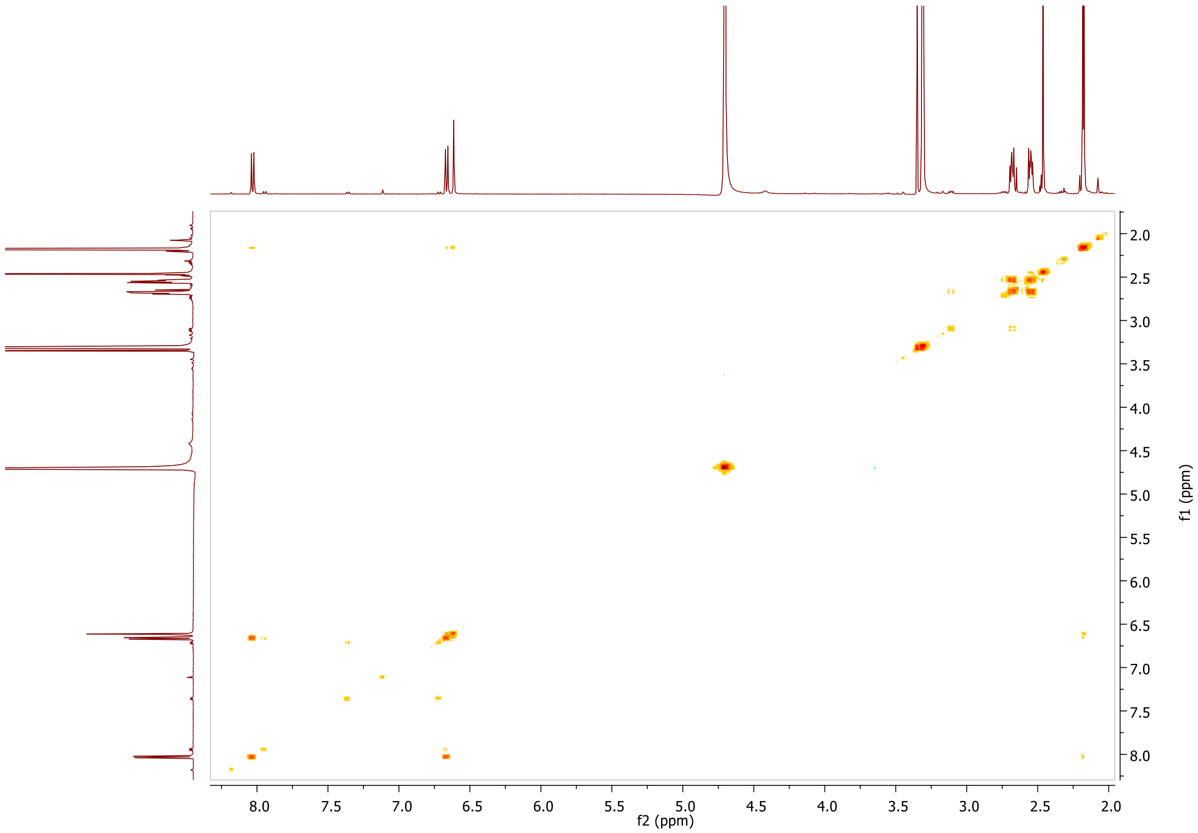

Figure S38. ^{1}H-^{1}H COSY spectrum of compound **7** (in CD_3OD).

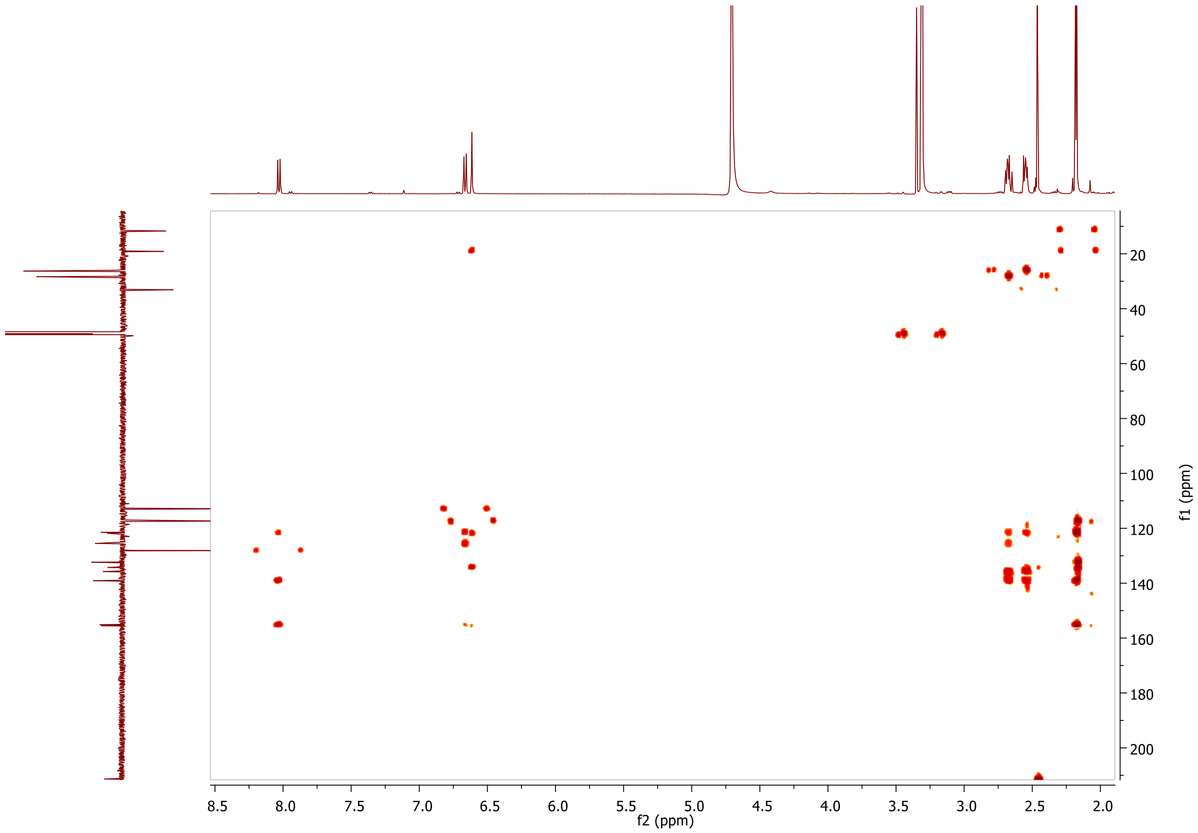

Figure S39. HMBC spectrum of compound **7** (in CD_3OD).

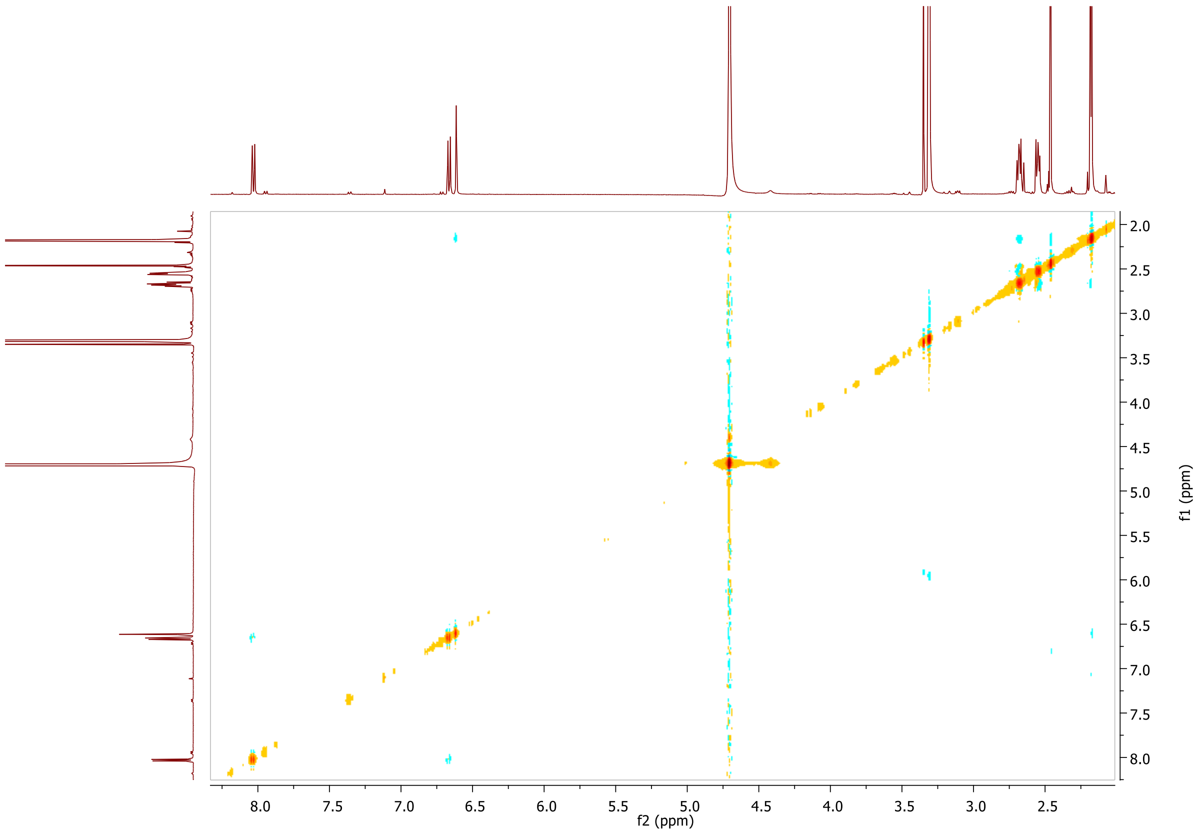

Figure S40. NOESY spectrum of compound **7** (in CD_3OD).

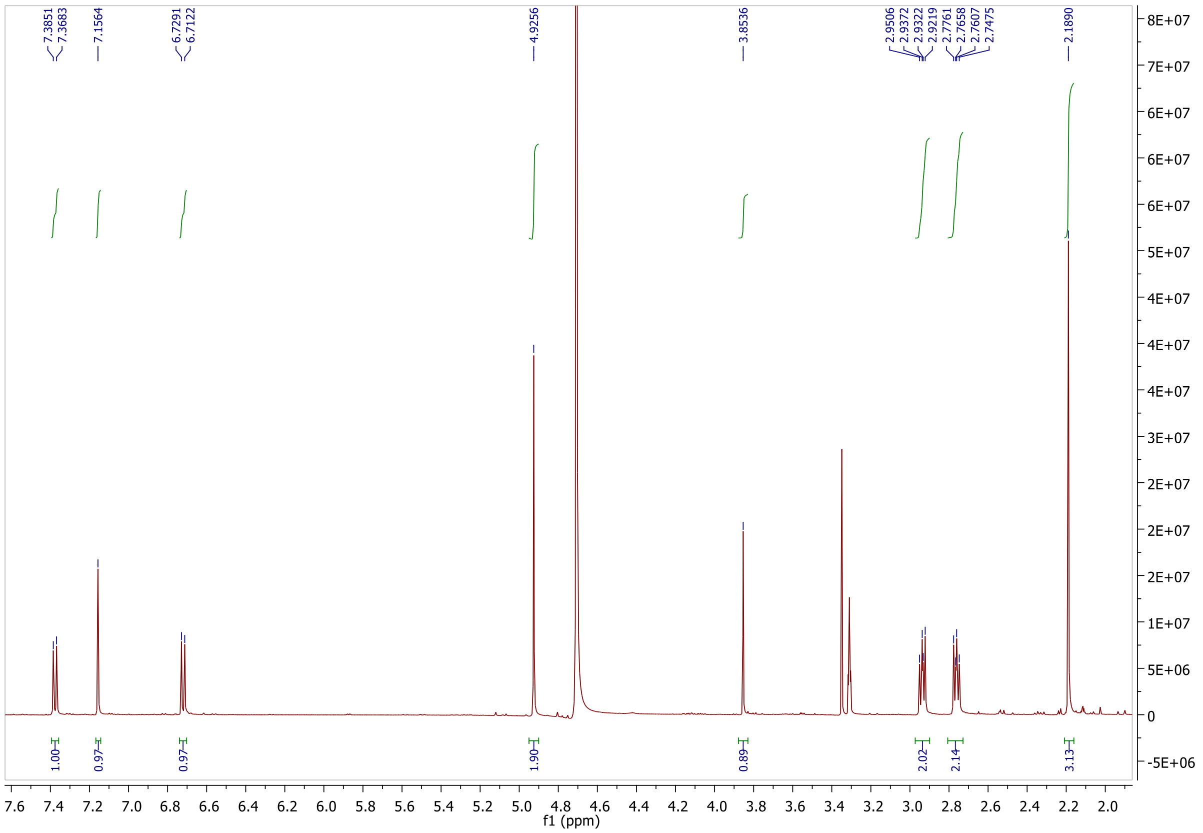

Figure S41. ^{1}H NMR spectrum of compound **8** (in CD_3OD).

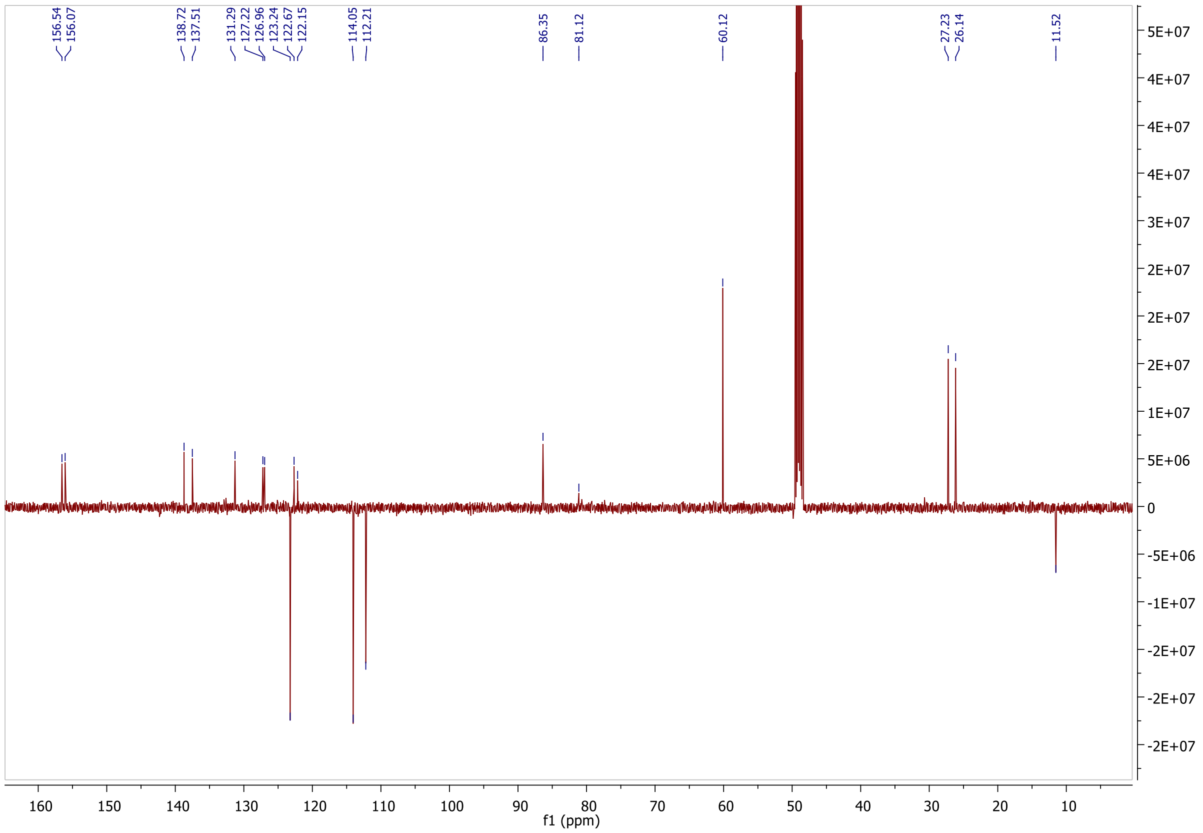

Figure S42. ^{13}C (JMOD) NMR spectrum of compound **8** (in CD_3OD).

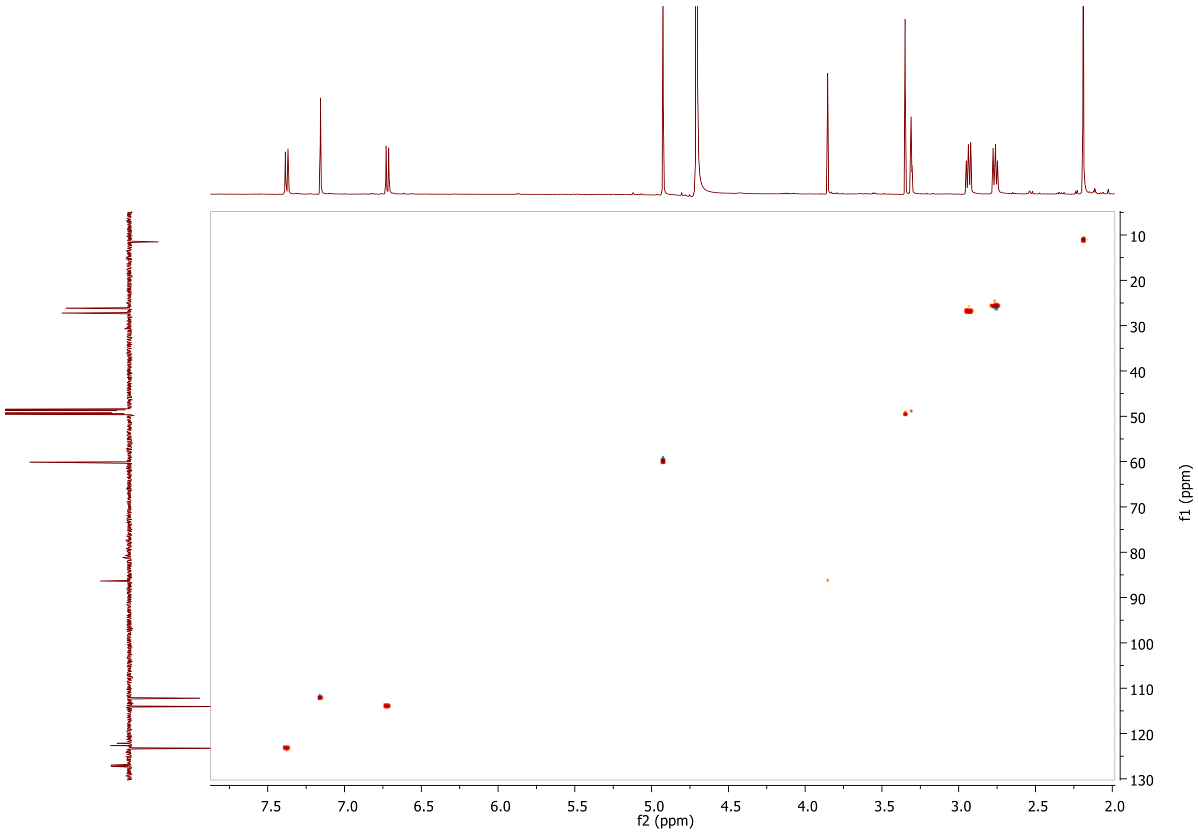

Figure S43. HSQC spectrum of compound **8** (in CD_3OD).

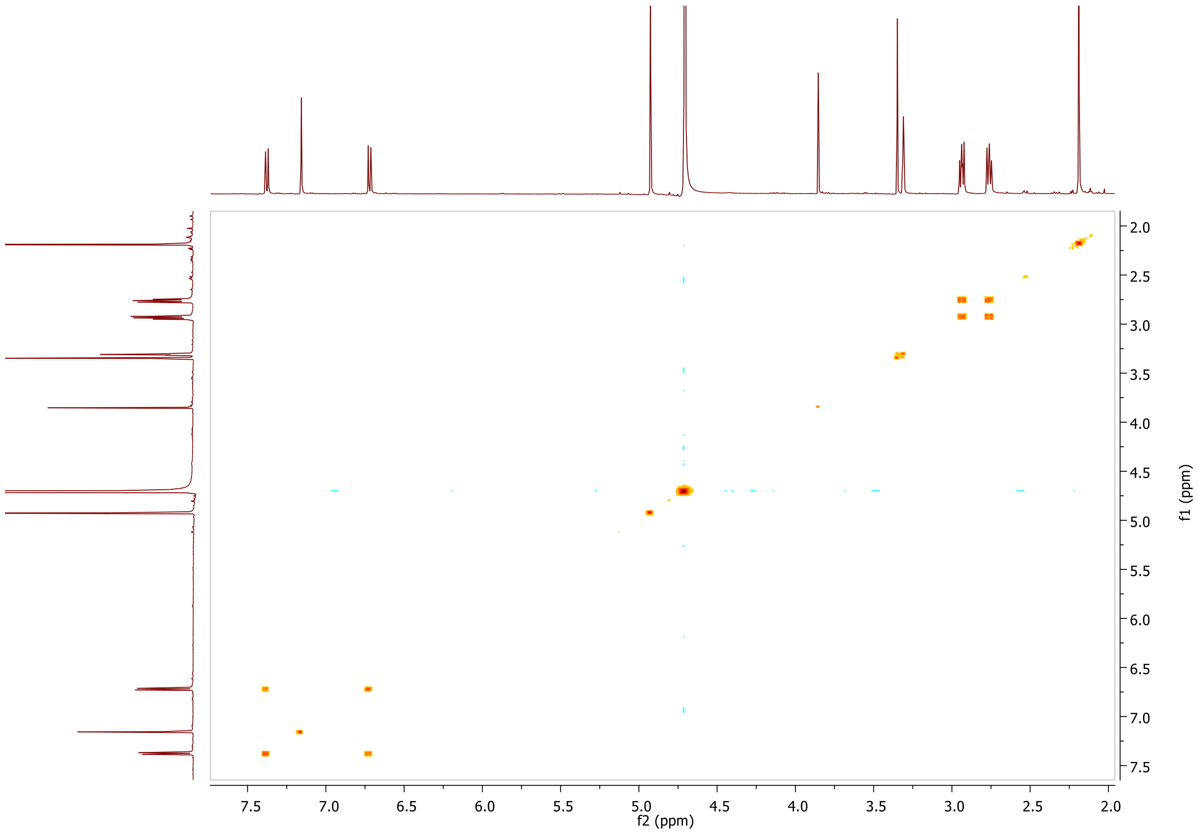

Figure S44. ^1H-^1H COSY spectrum of compound **8** (in CD_3OD).

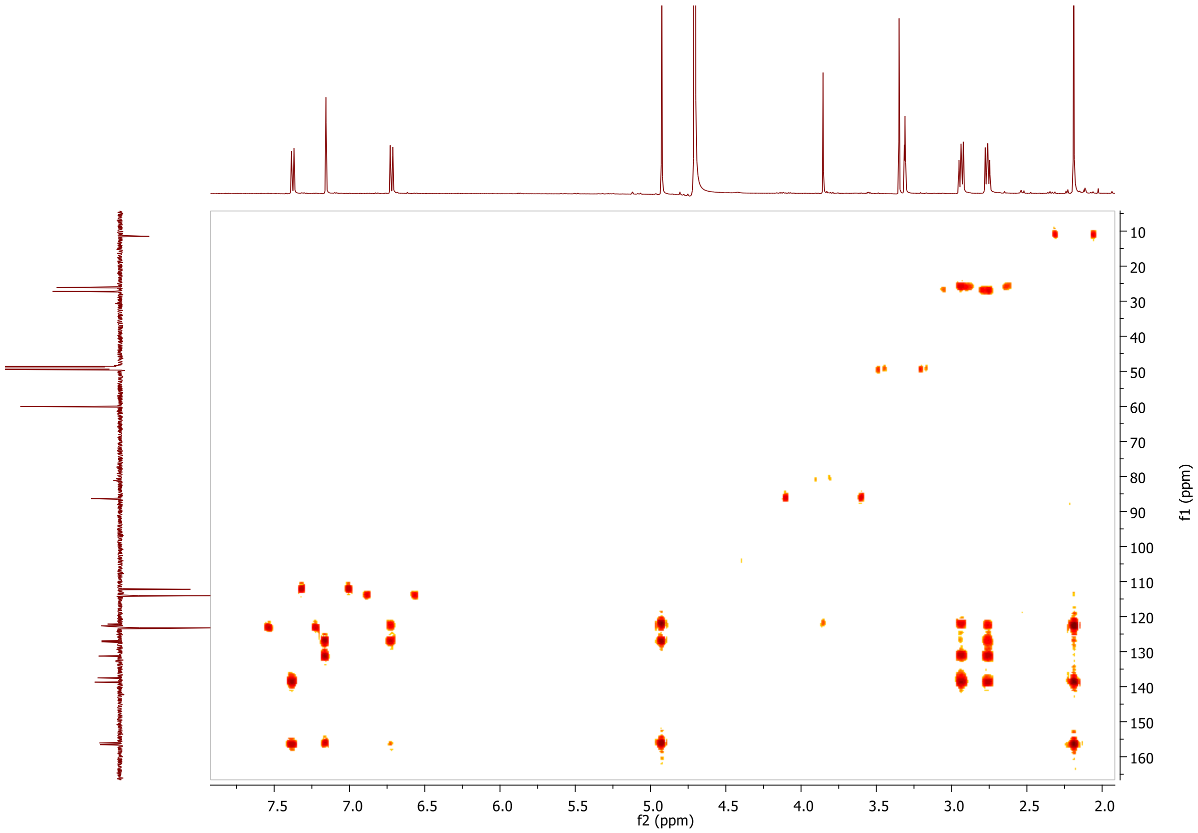

Figure S45. HMBC spectrum of compound **8** (in CD_3OD).

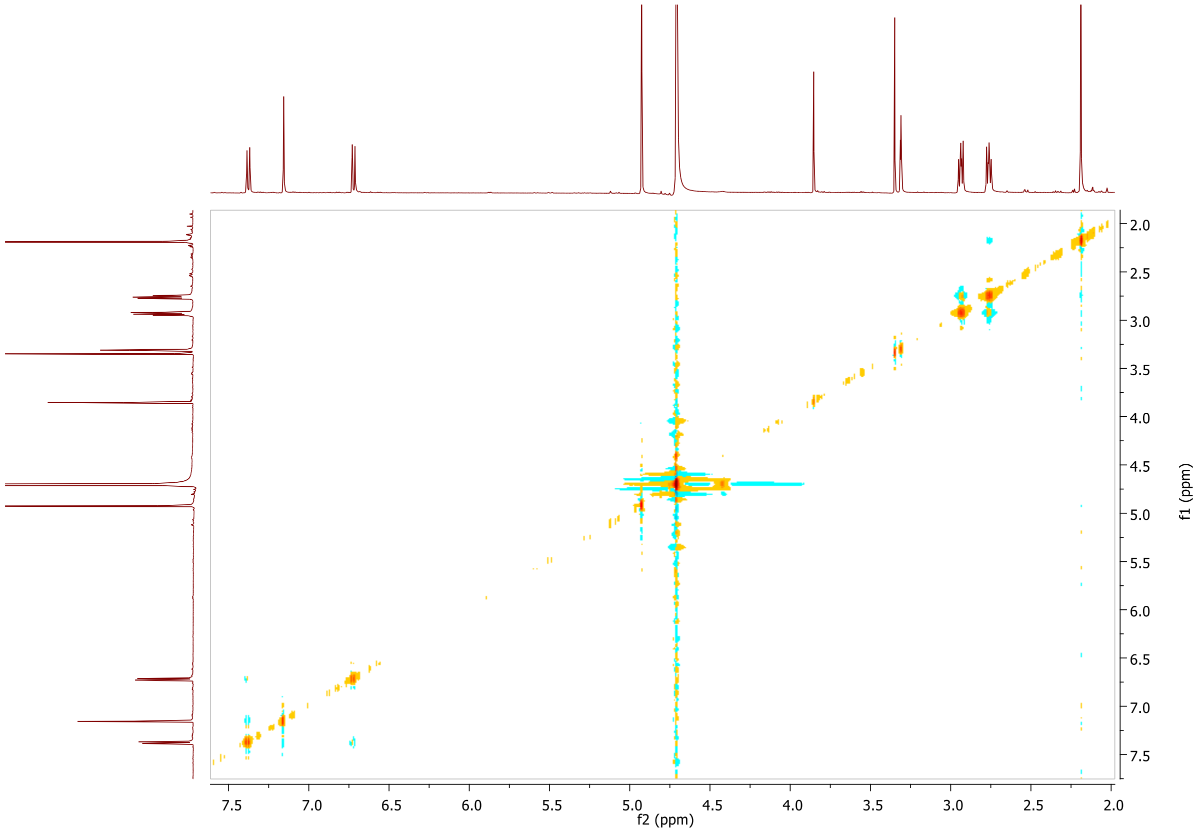

Figure S46. NOESY spectrum of compound **8** (in CD_3OD).

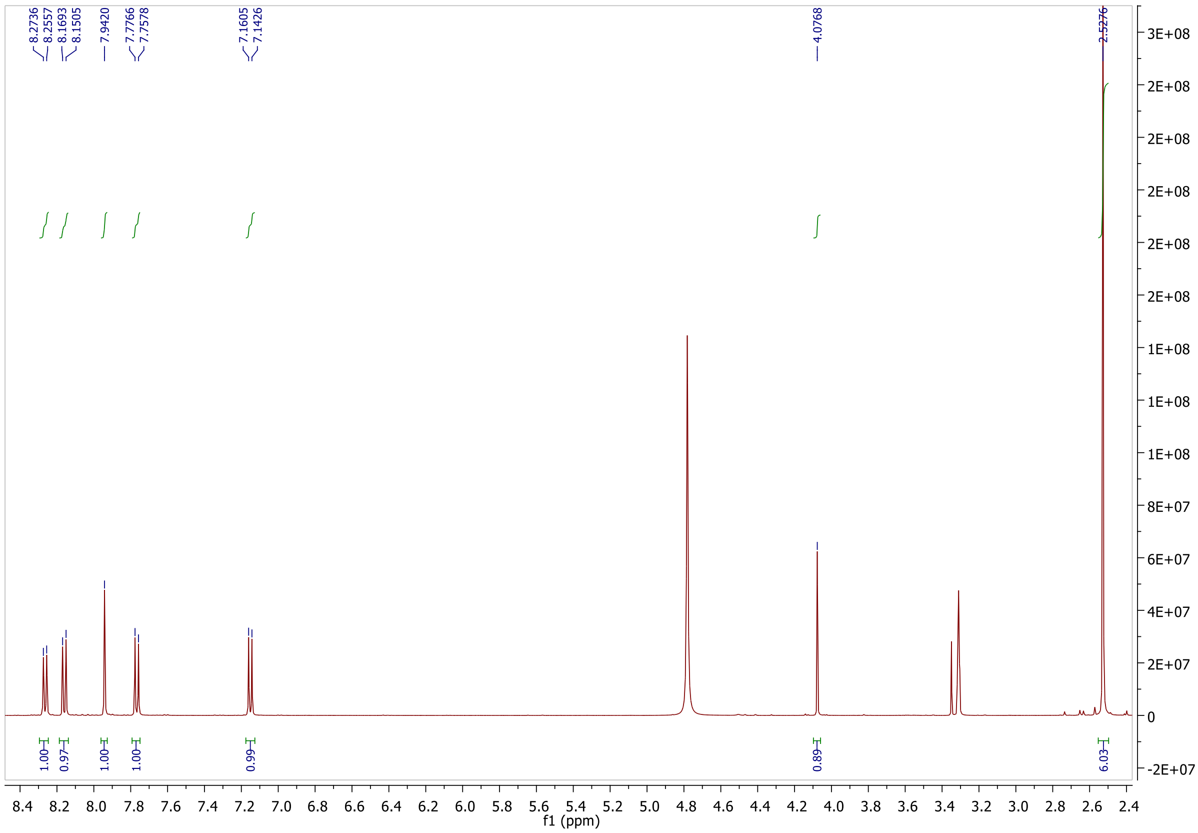

Figure S47. ^{1}H NMR spectrum of compound **9** (in $CDCl_3$).

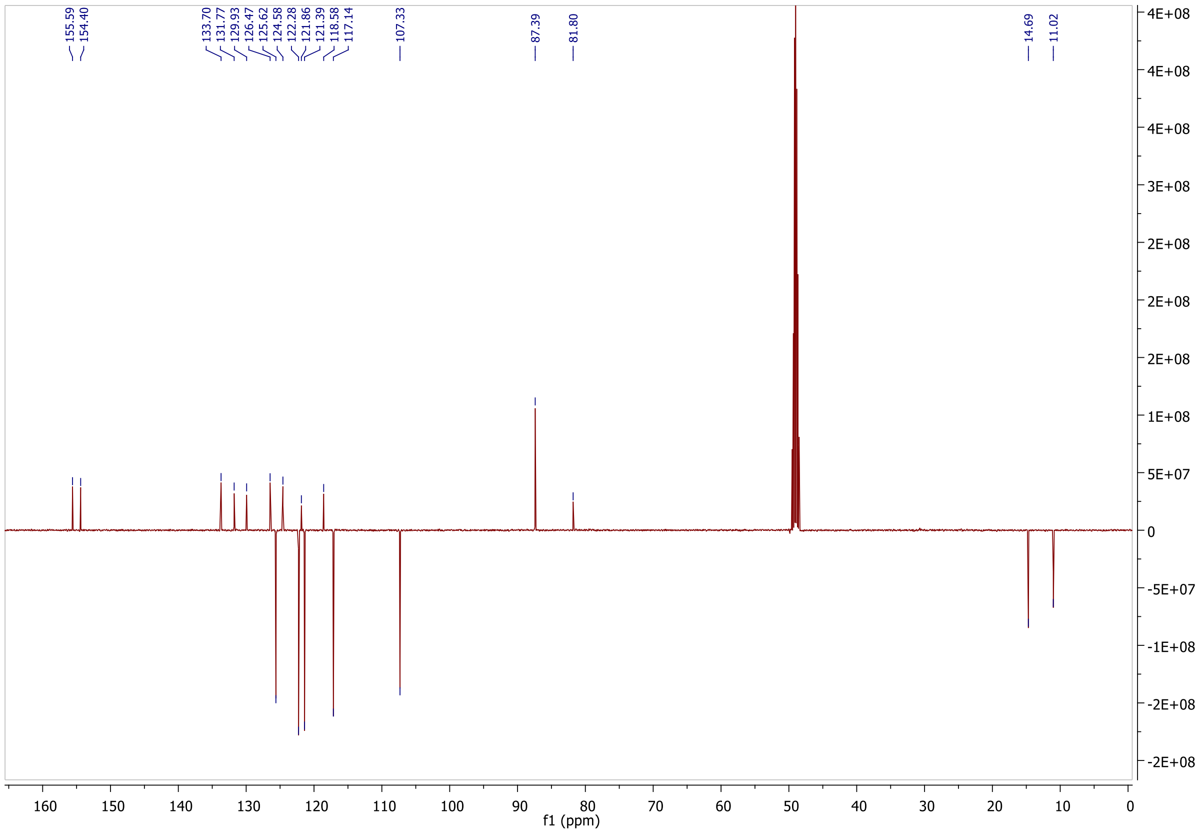

Figure S48. ^{13}C (JMOD) NMR spectrum of compound **9** (in $CDCl_3$).

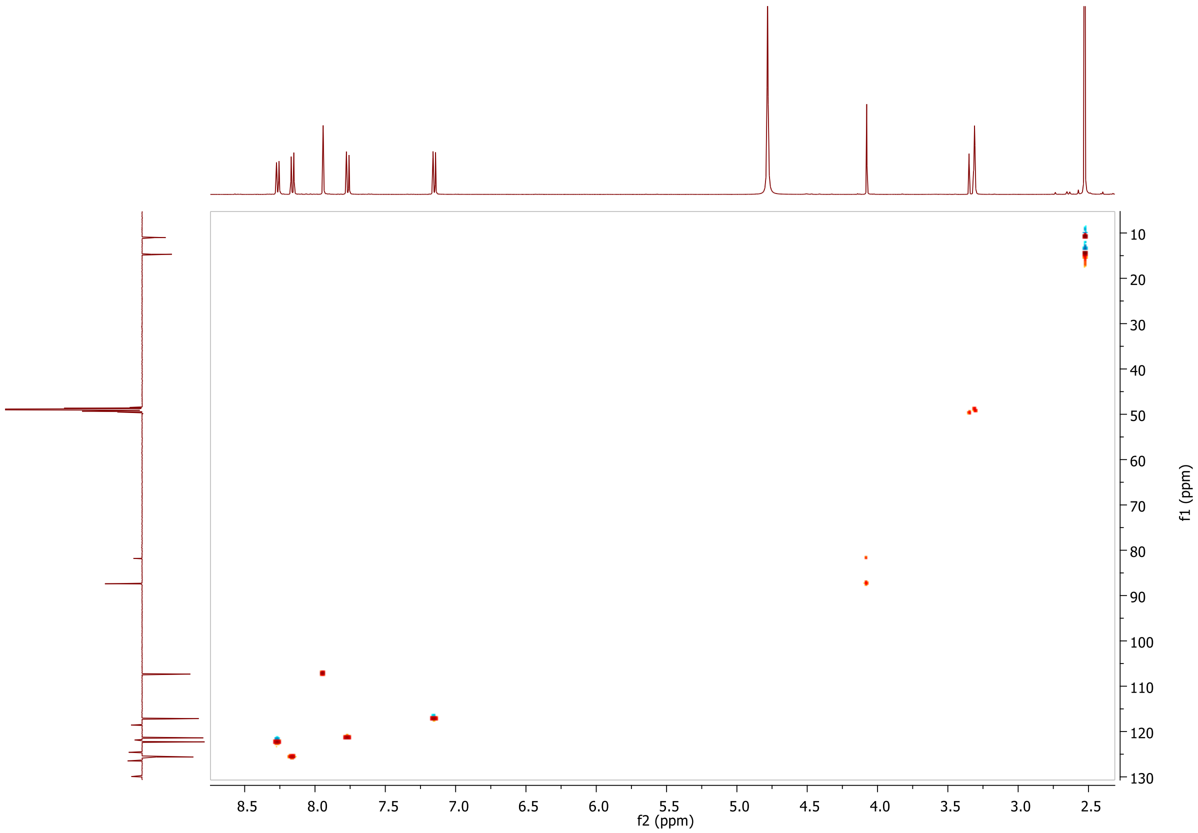

Figure S49. HSQC spectrum of compound **9** (in $CDCl_3$).

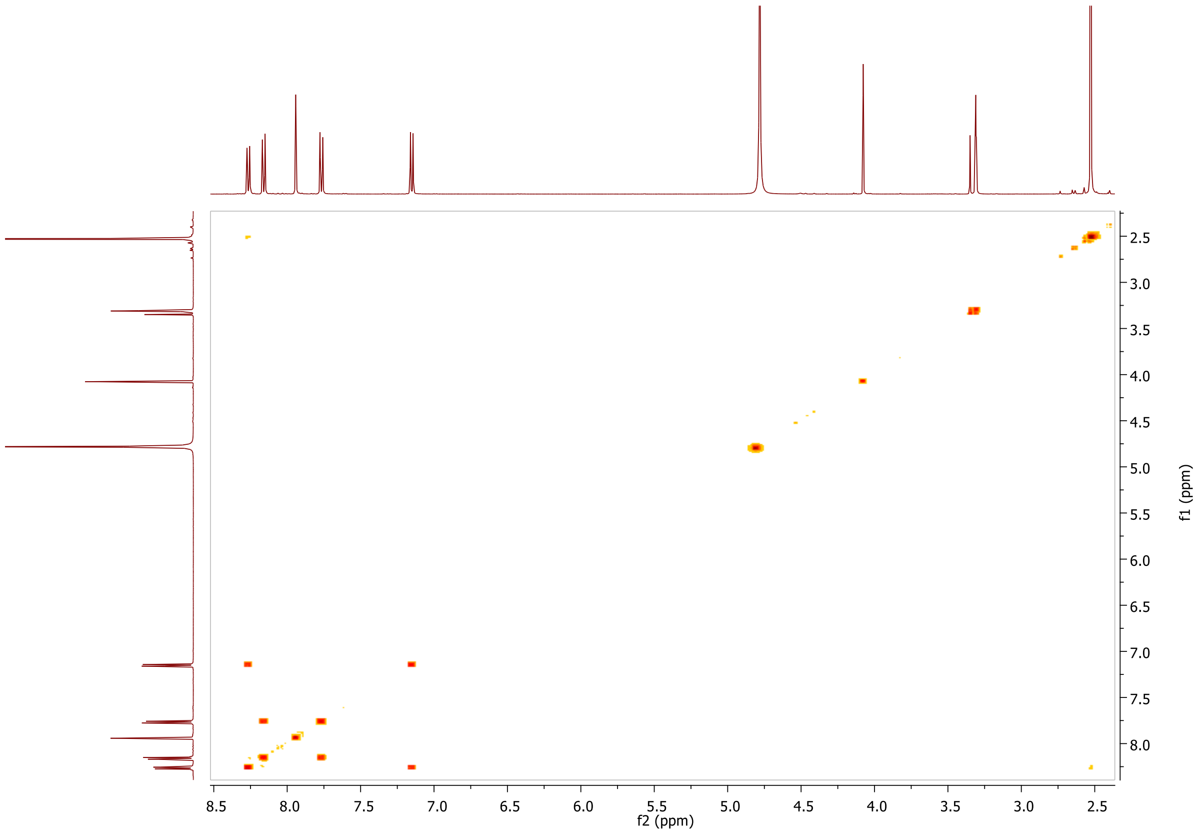

Figure S50. ${}^{1}H$-${}^{1}H$ COSY spectrum of compound **9** (in $CDCl_3$).

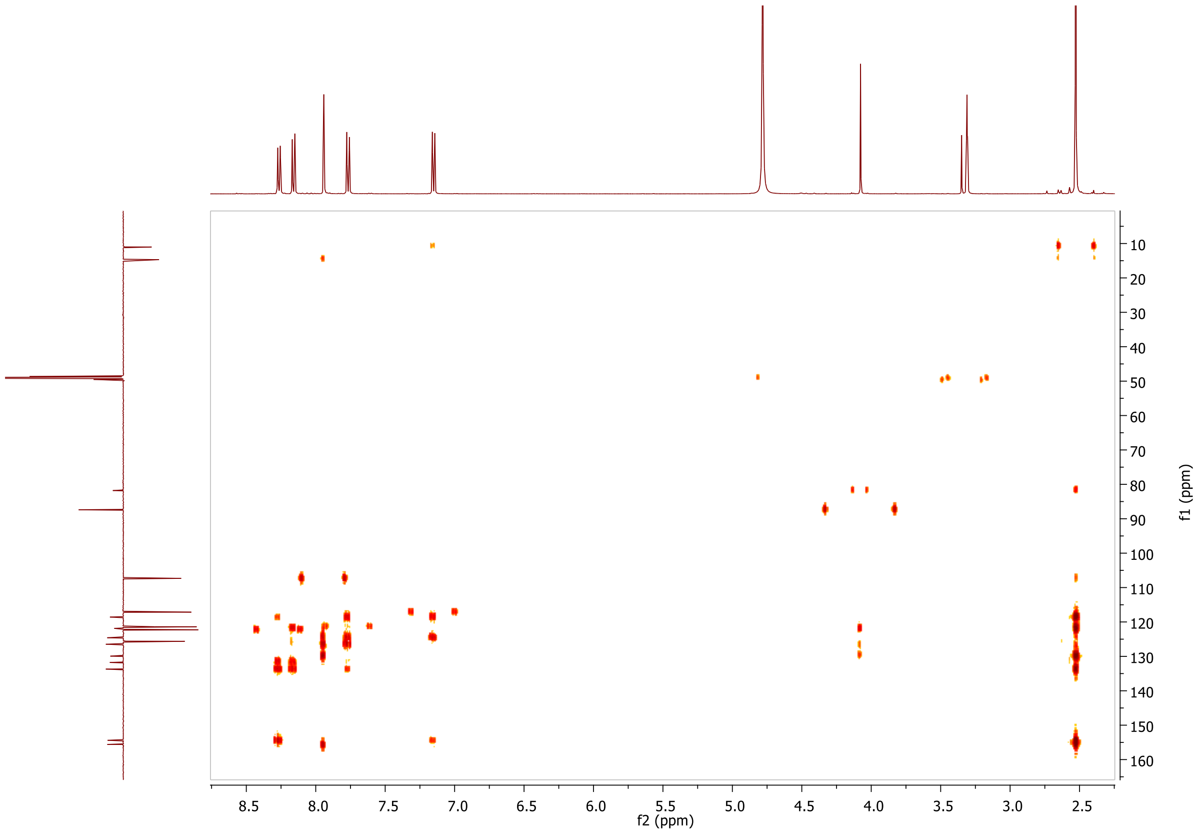

Figure S51. HMBC spectrum of compound **9** (in $CDCl_3$).

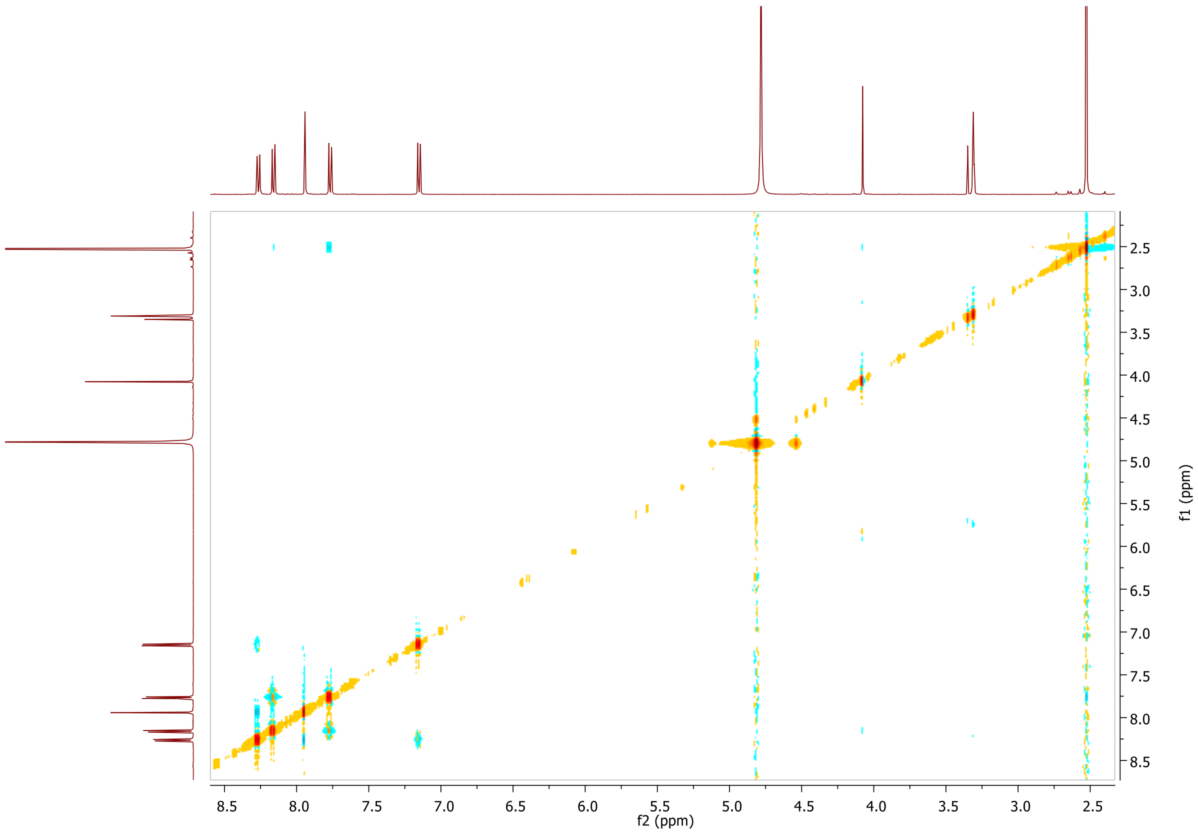

Figure S52. NOESY spectrum of compound **9** (in $CDCl_3$).

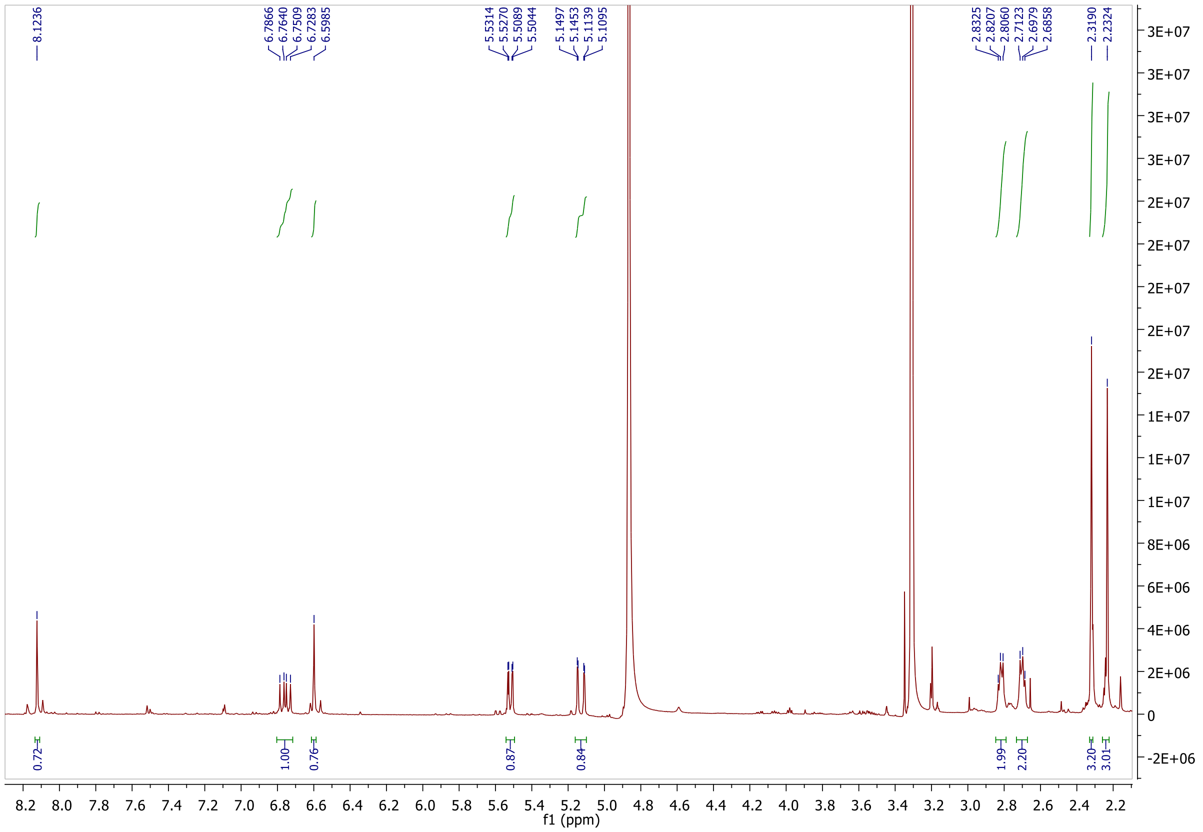

Figure S53. ^{1}H NMR spectrum of compound **10** (in CD_3OD).

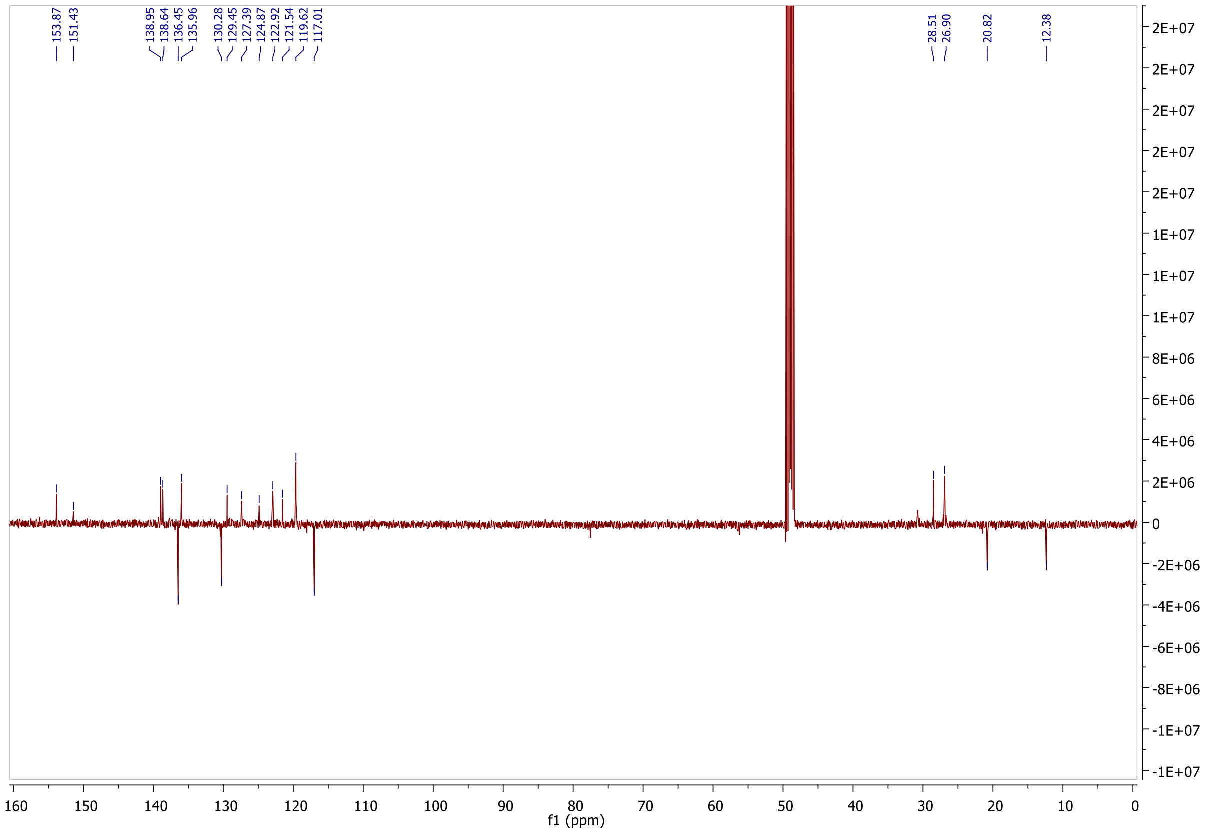

Figure S54. ^{13}C (JMOD) NMR spectrum of compound **10** (in CD_3OD).

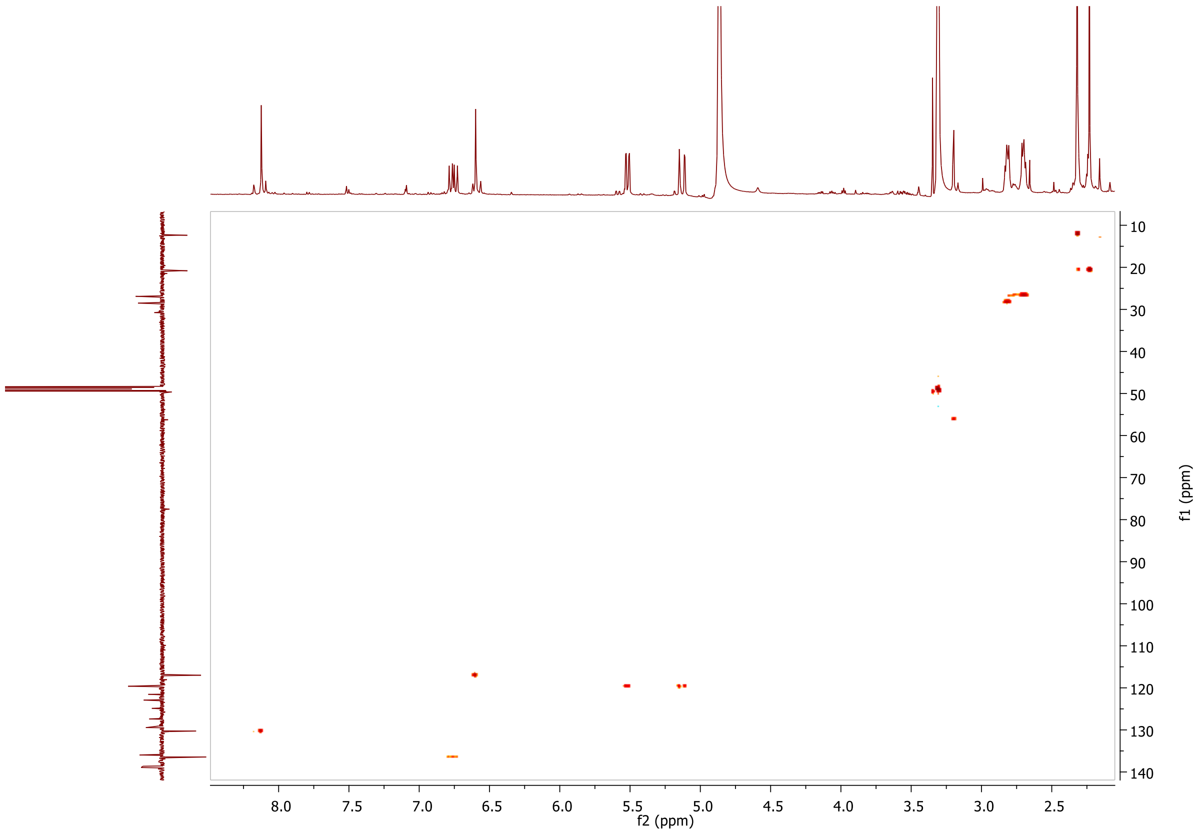

Figure S55. HSQC spectrum of compound **10** (in CD_3OD).

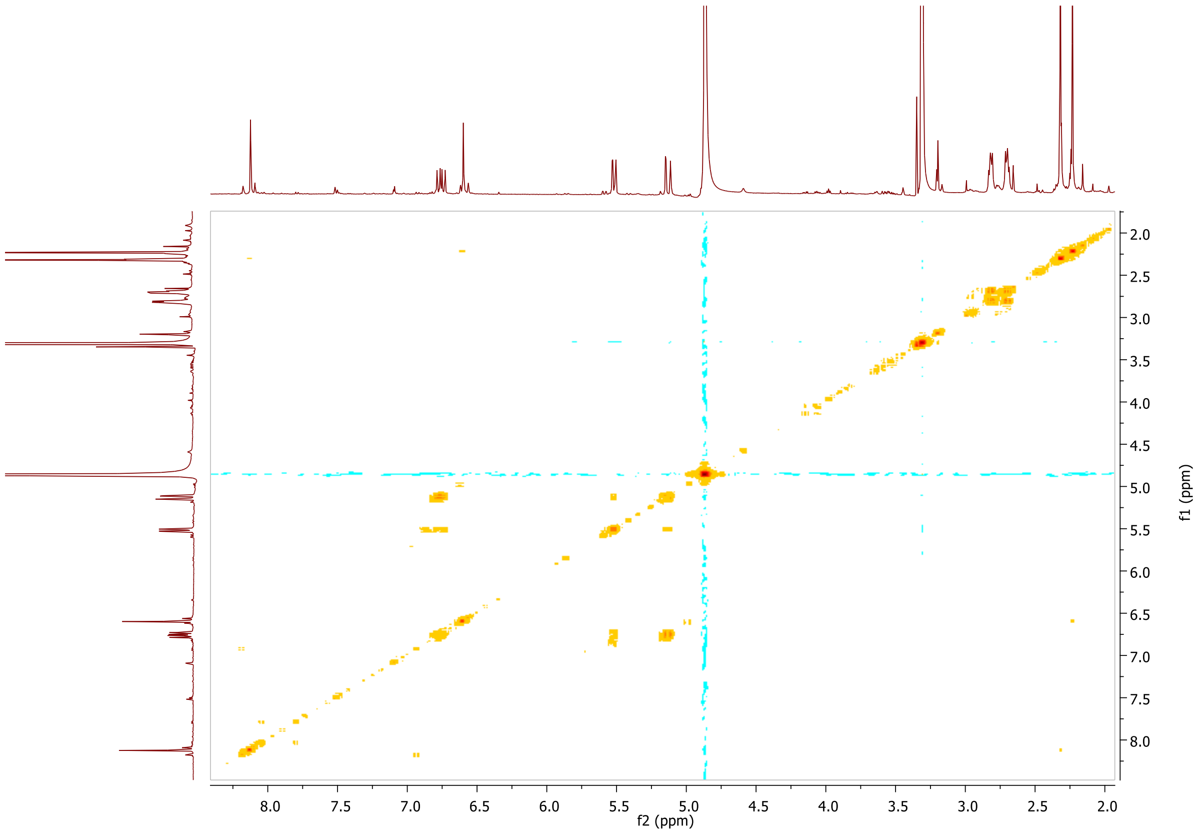

Figure S56. ^1H-^1H COSY spectrum of compound **10** (in CD_3OD).

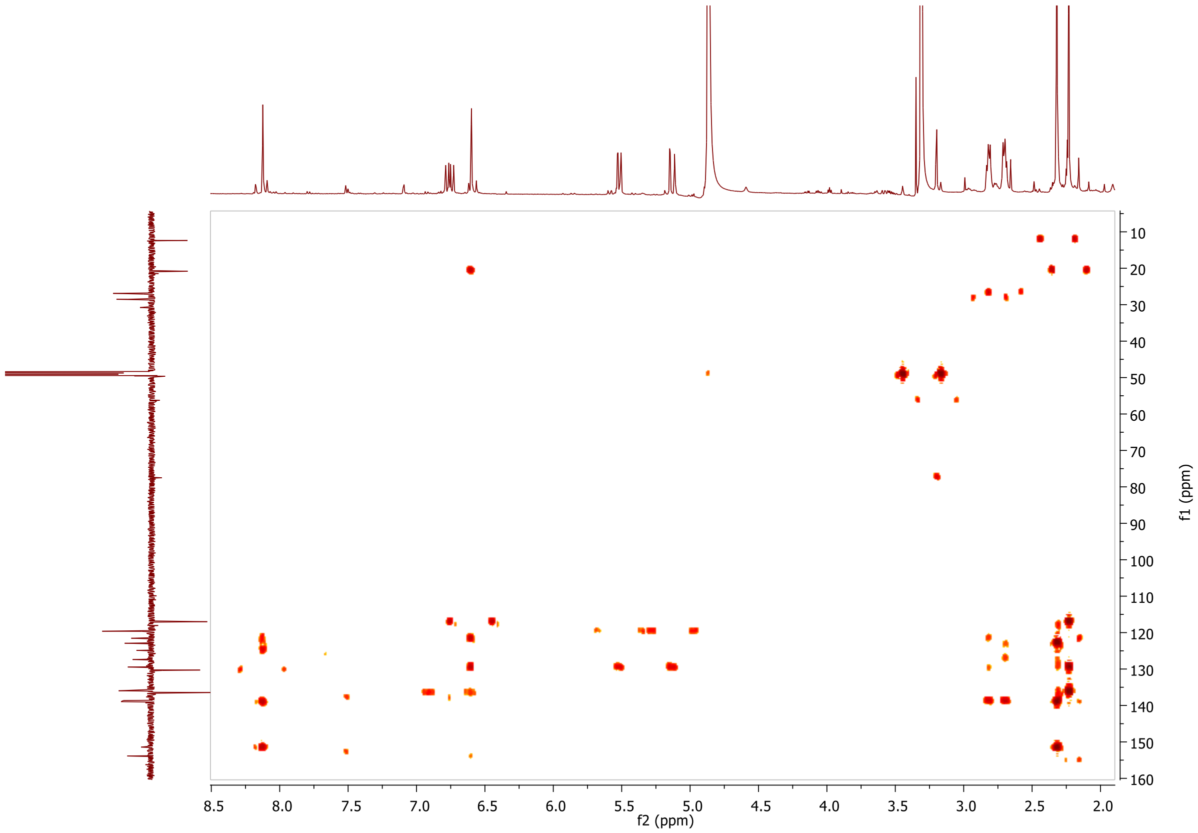

Figure S57. HMBC spectrum of compound **10** (in CD_3OD).

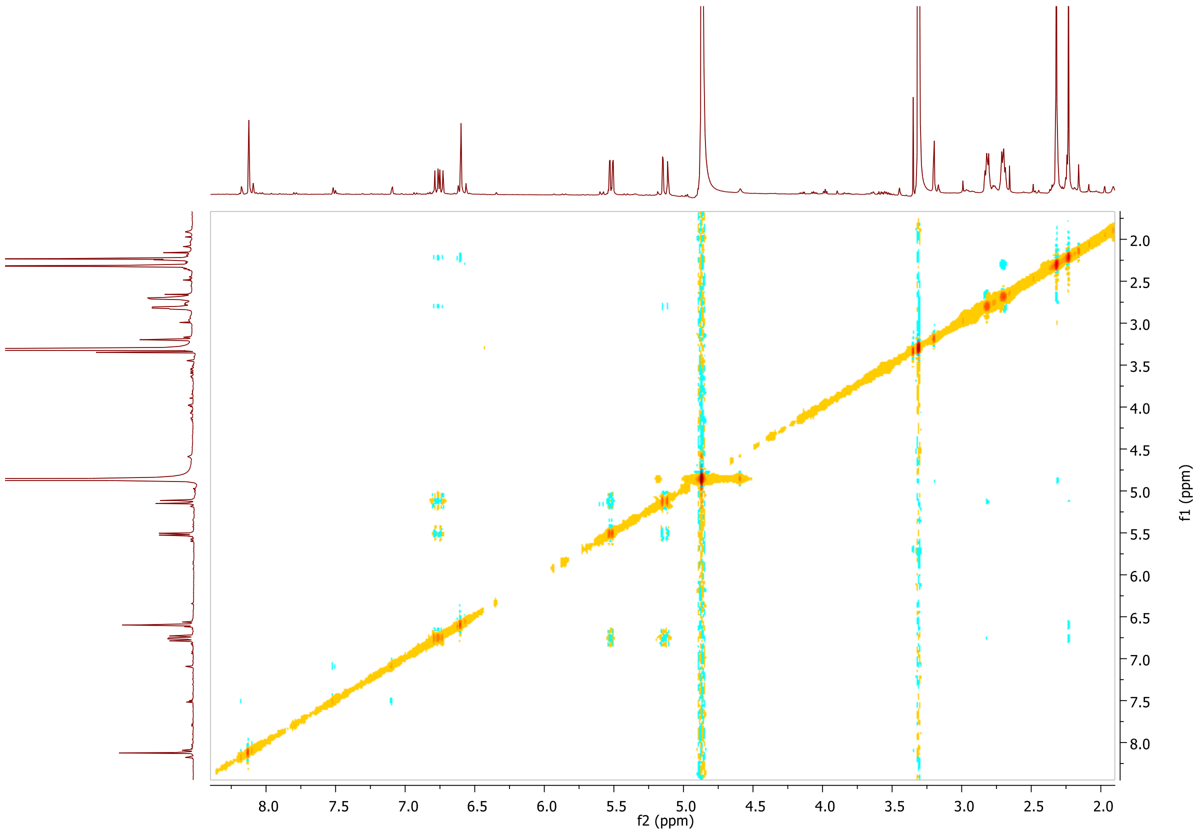

Figure S58. NOESY spectrum of compound **10** (in CD_3OD).

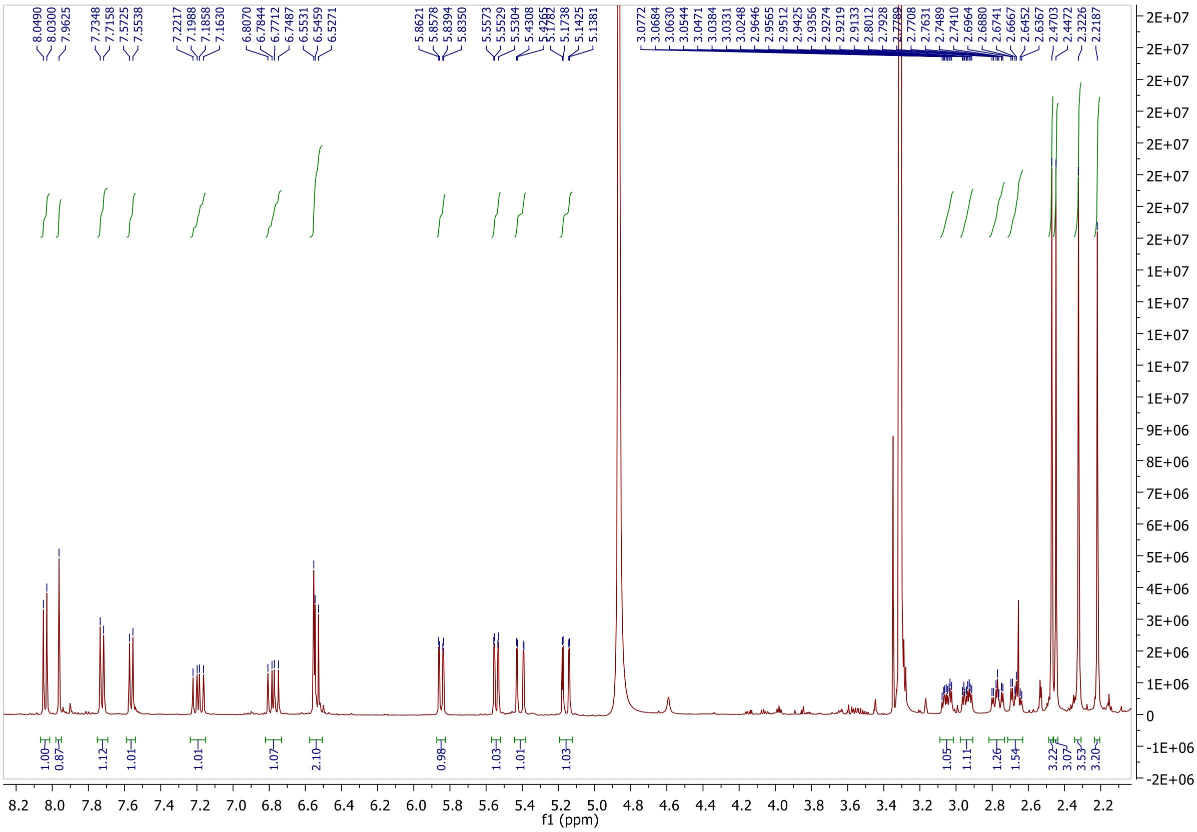

Figure S59. ^1H NMR spectrum of compound **11** (in CD_3OD).

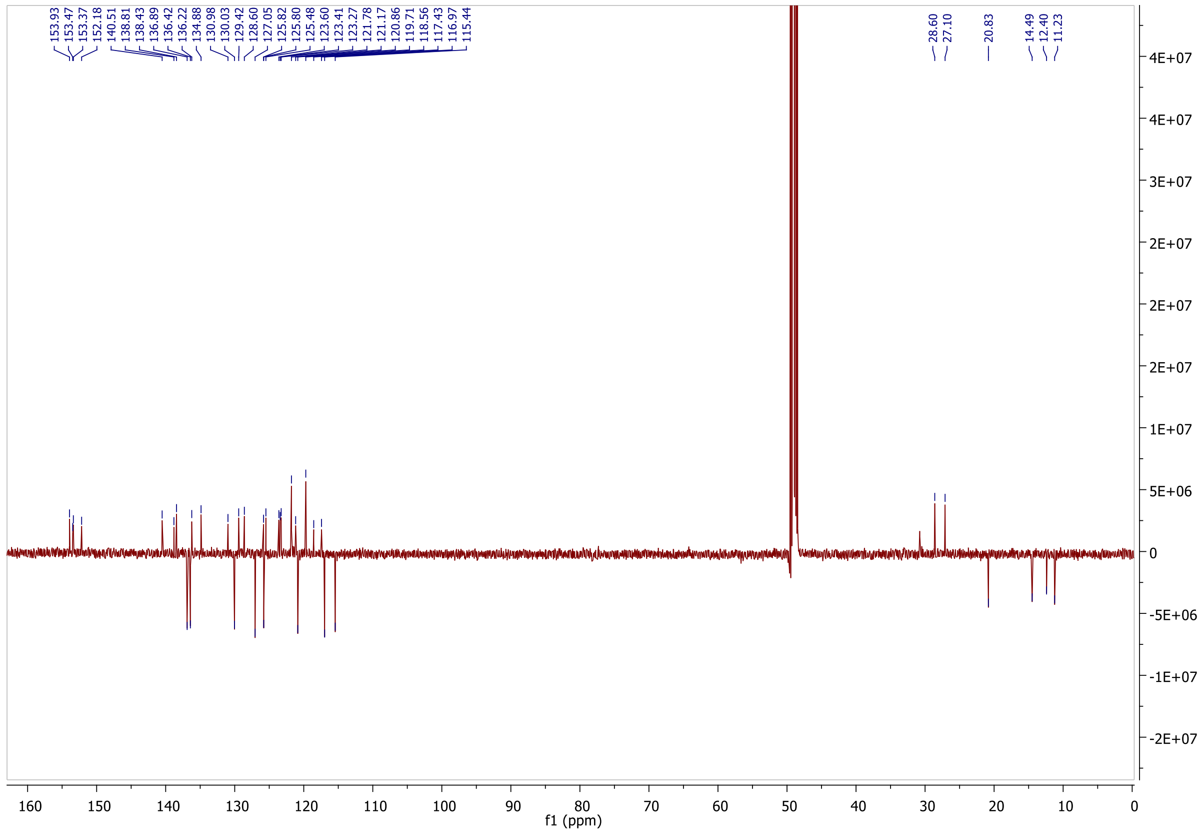

Figure S60. ^{13}C (JMOD) NMR spectrum of compound **11** (in CD_3OD).

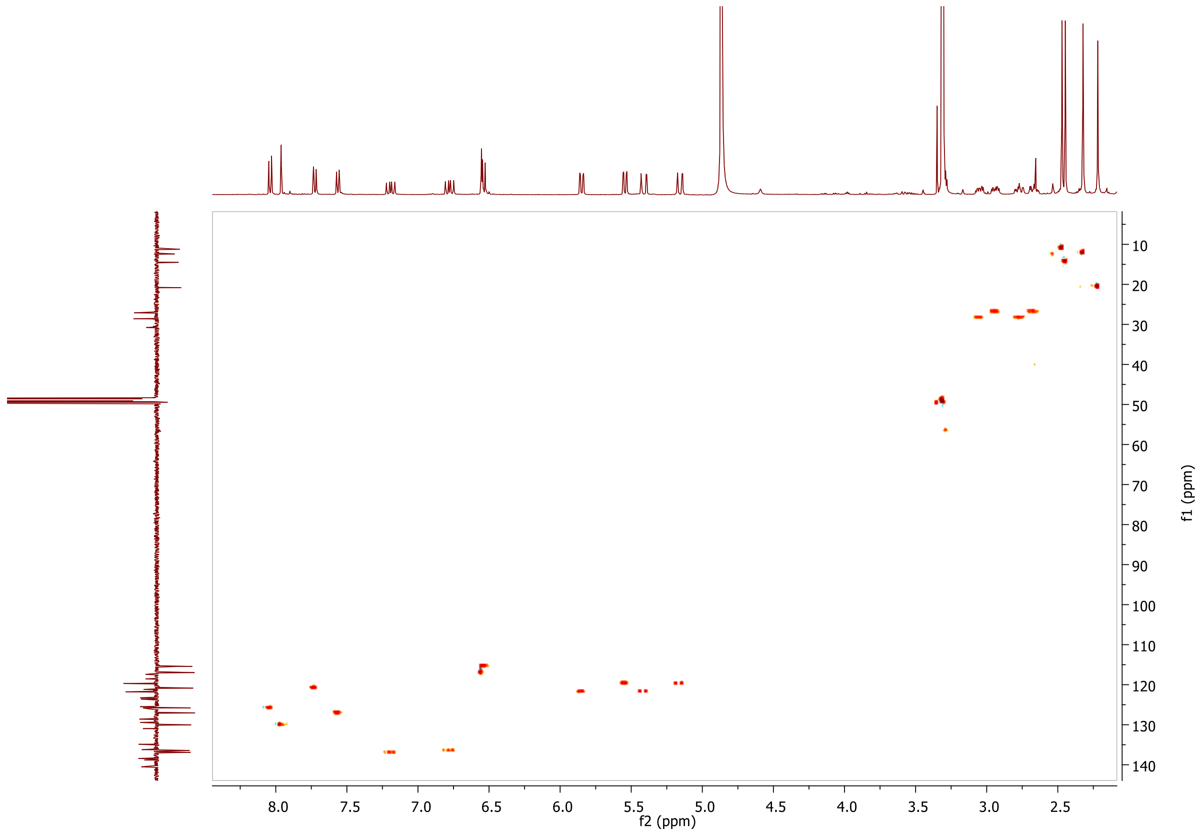

Figure S61. HSQC spectrum of compound **11** (in CD_3OD).

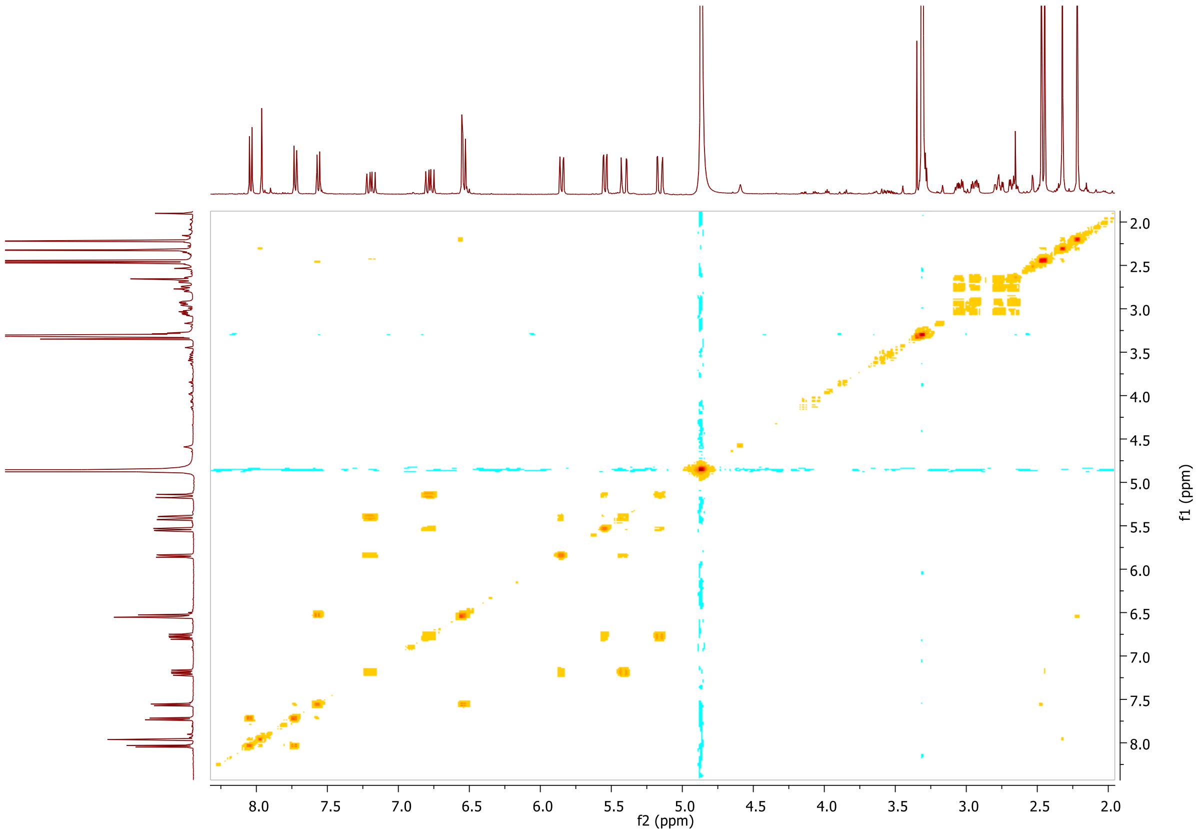

Figure S62. ^{1}H-^{1}H COSY spectrum of compound **11** (in CD_3OD).

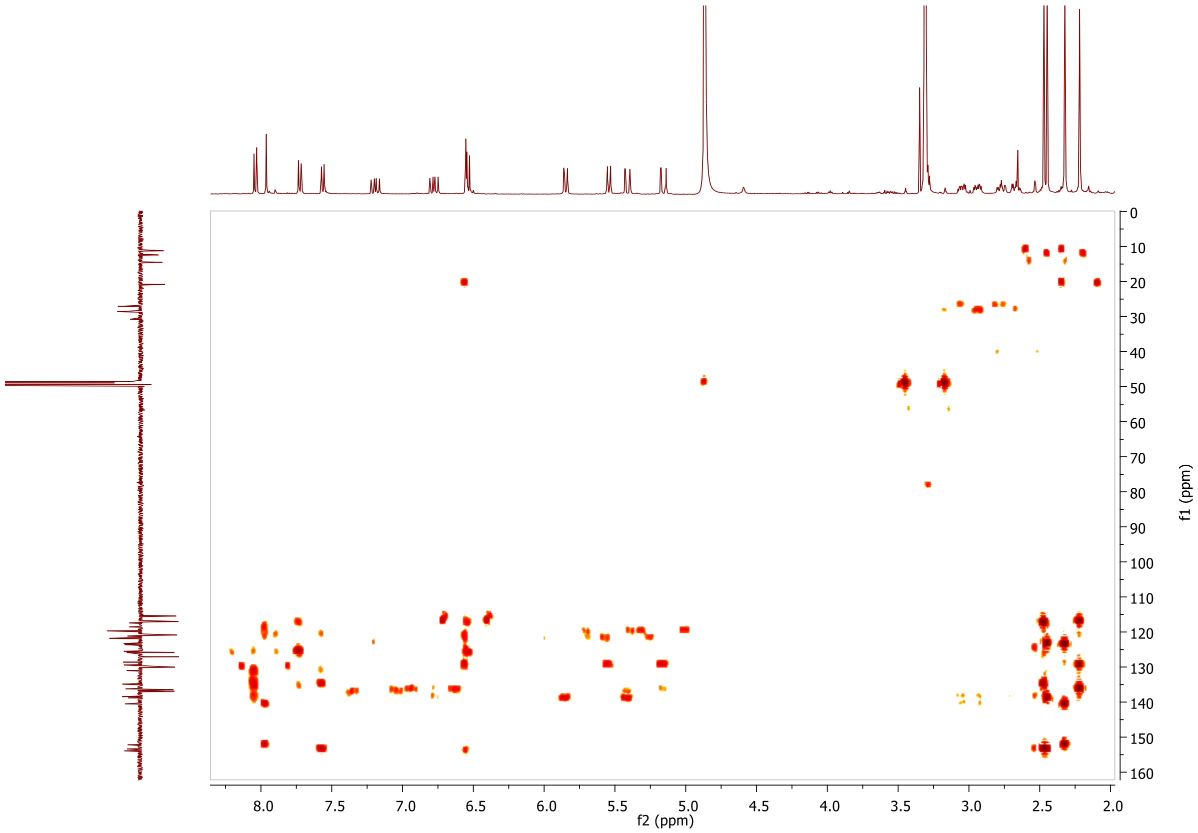

Figure S63. HMBC spectrum of compound **11** (in CD_3OD).

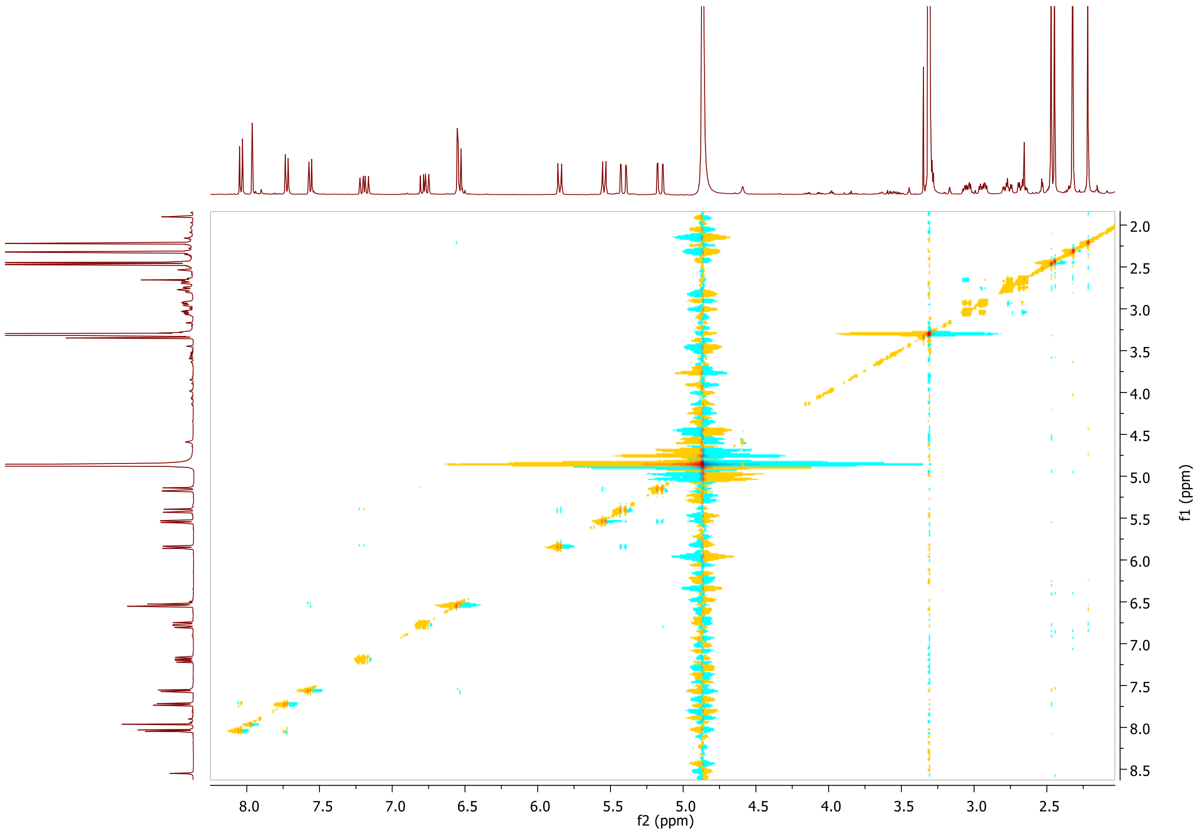

Figure S64. NOESY spectrum of compound **11** (in CD_3OD).

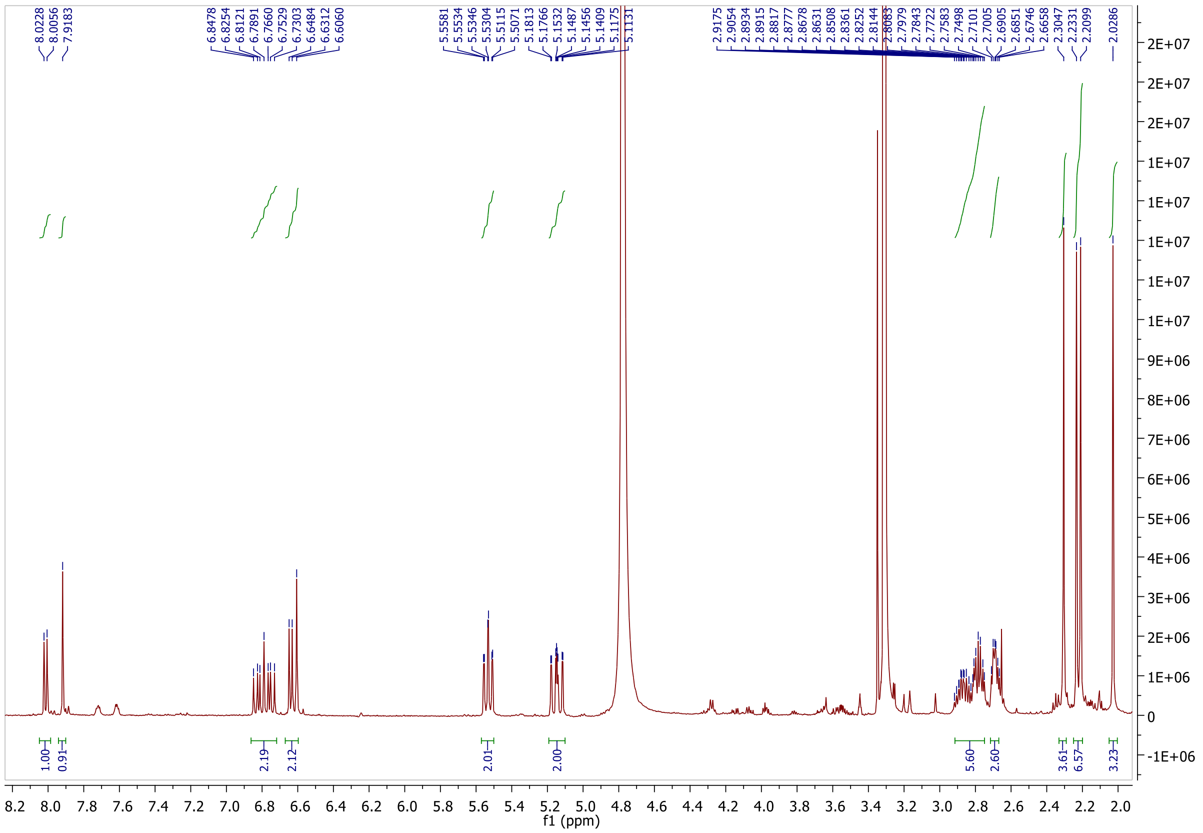

Figure S65. ^{1}H NMR spectrum of compound **12** (in CD_3OD).

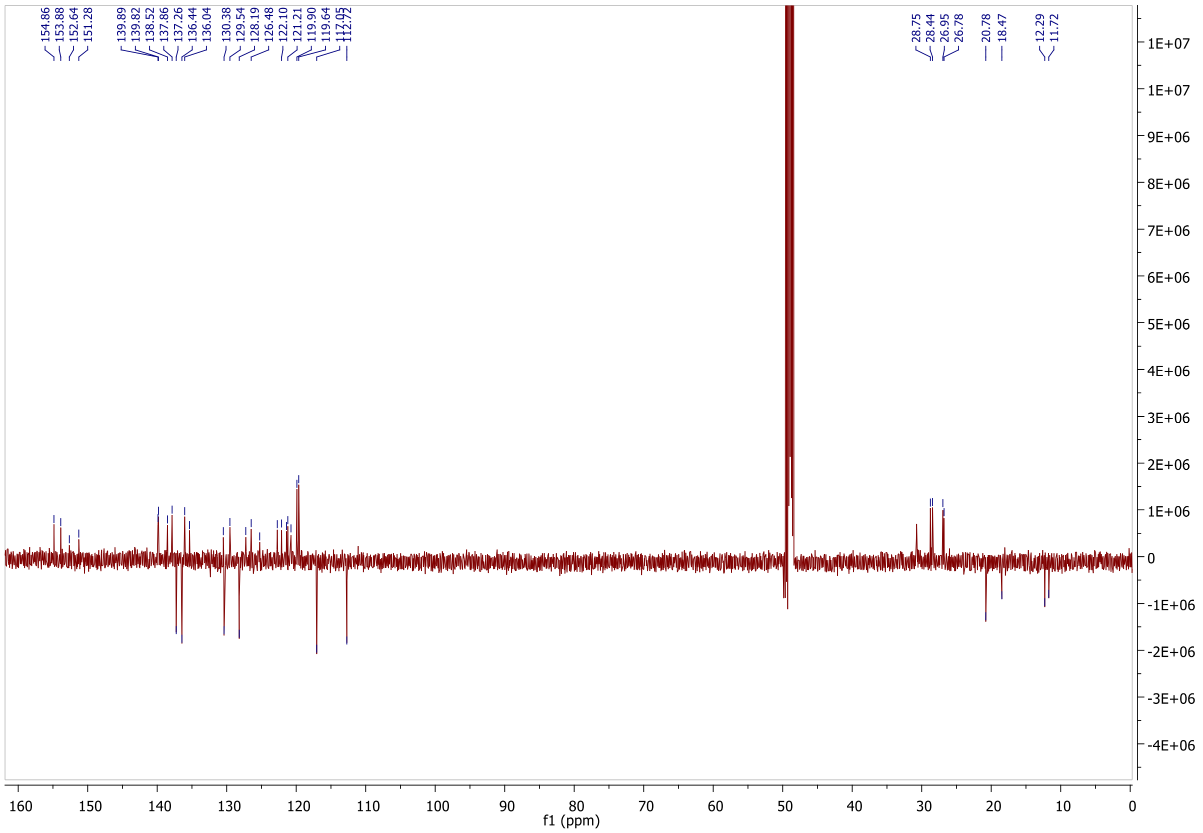

Figure S66. ^{13}C (JMOD) NMR spectrum of compound **12** (in CD_3OD).

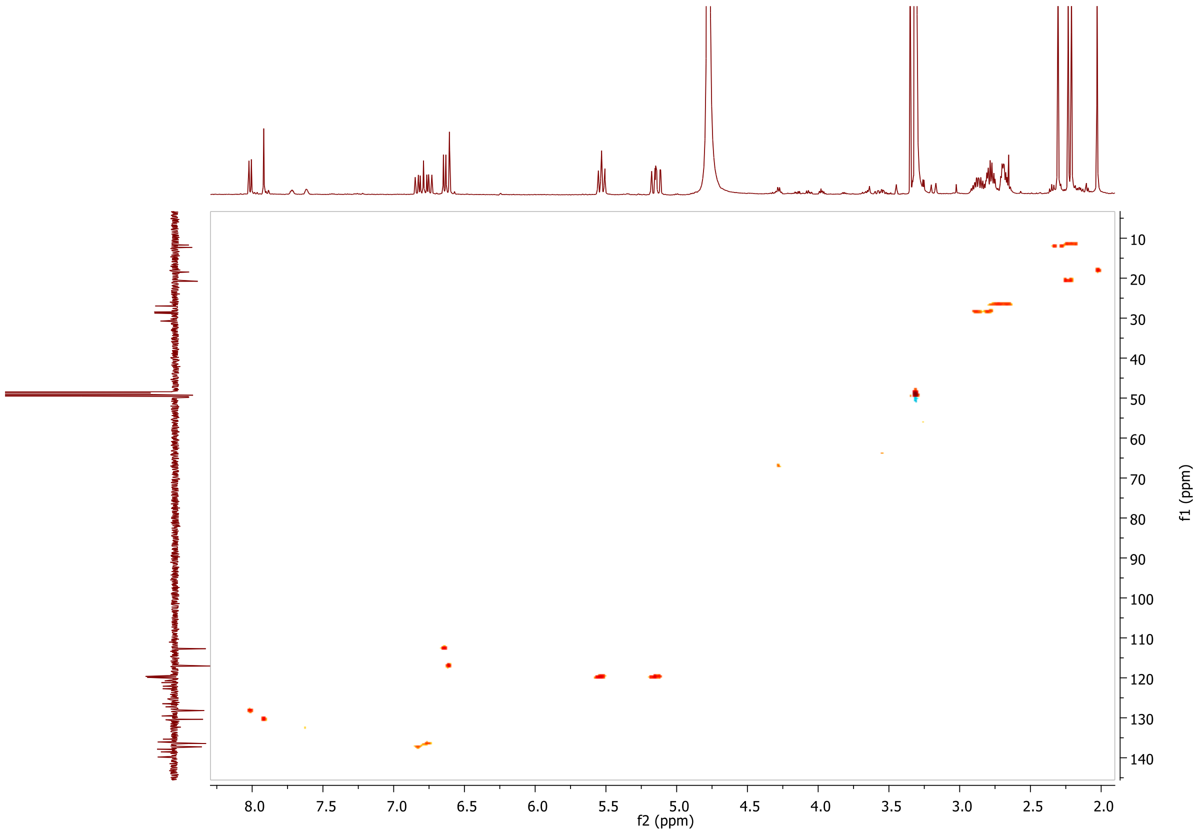

Figure S67. HSQC spectrum of compound **12** (in CD_3OD).

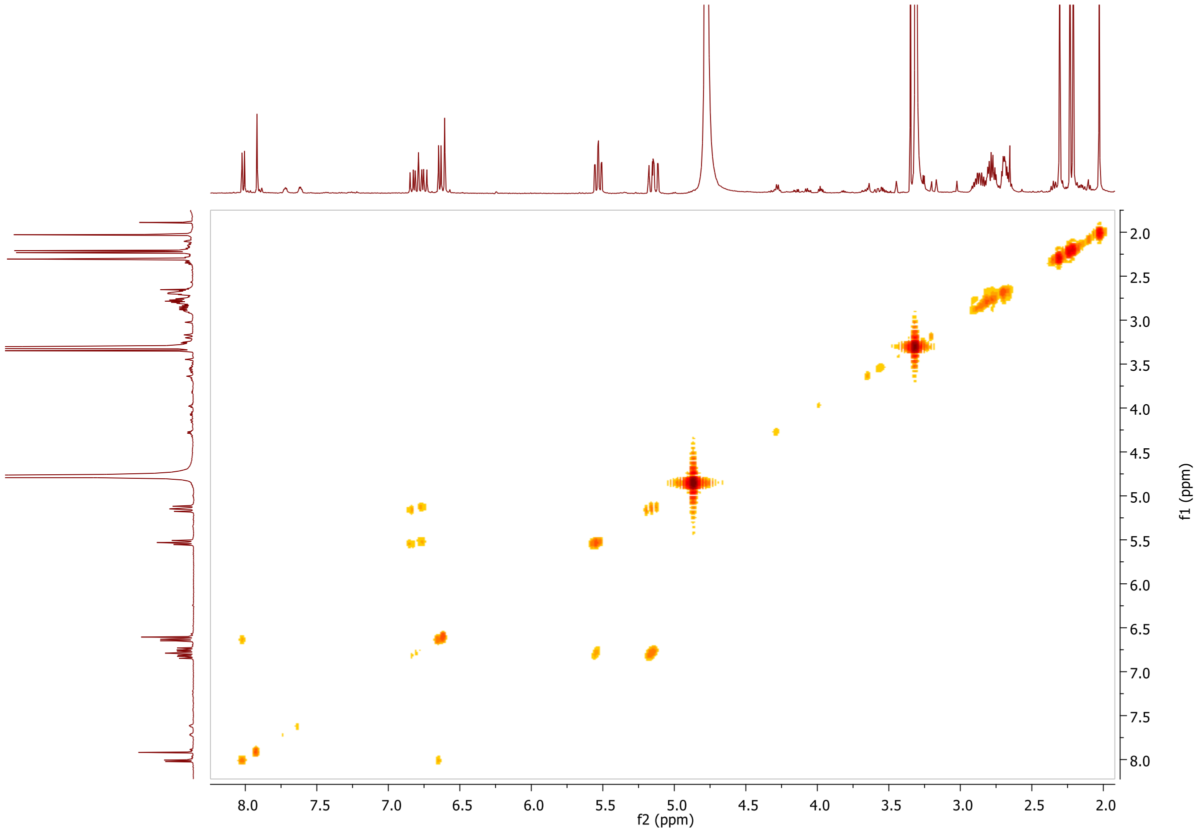

Figure S68. ¹H-¹H COSY spectrum of compound **12** (in CD_3OD).

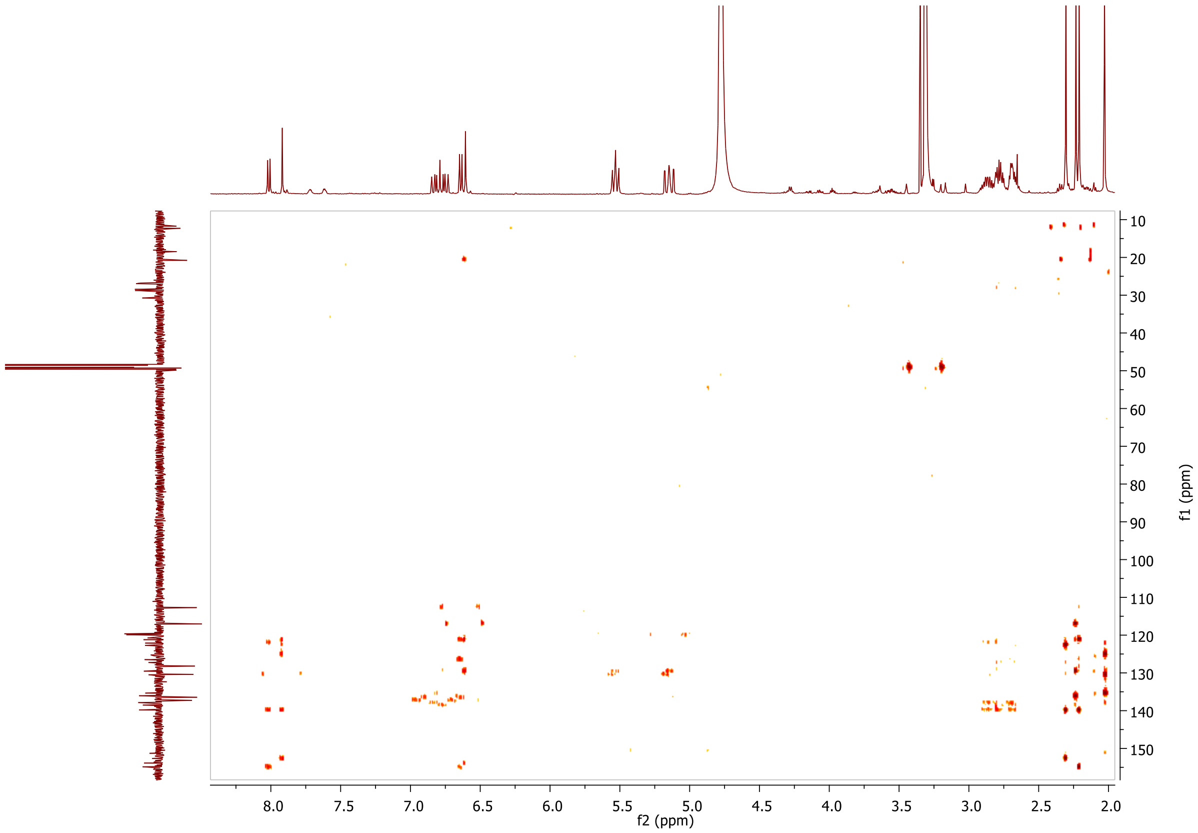

Figure S69. HMBC spectrum of compound **12** (in CD_3OD).

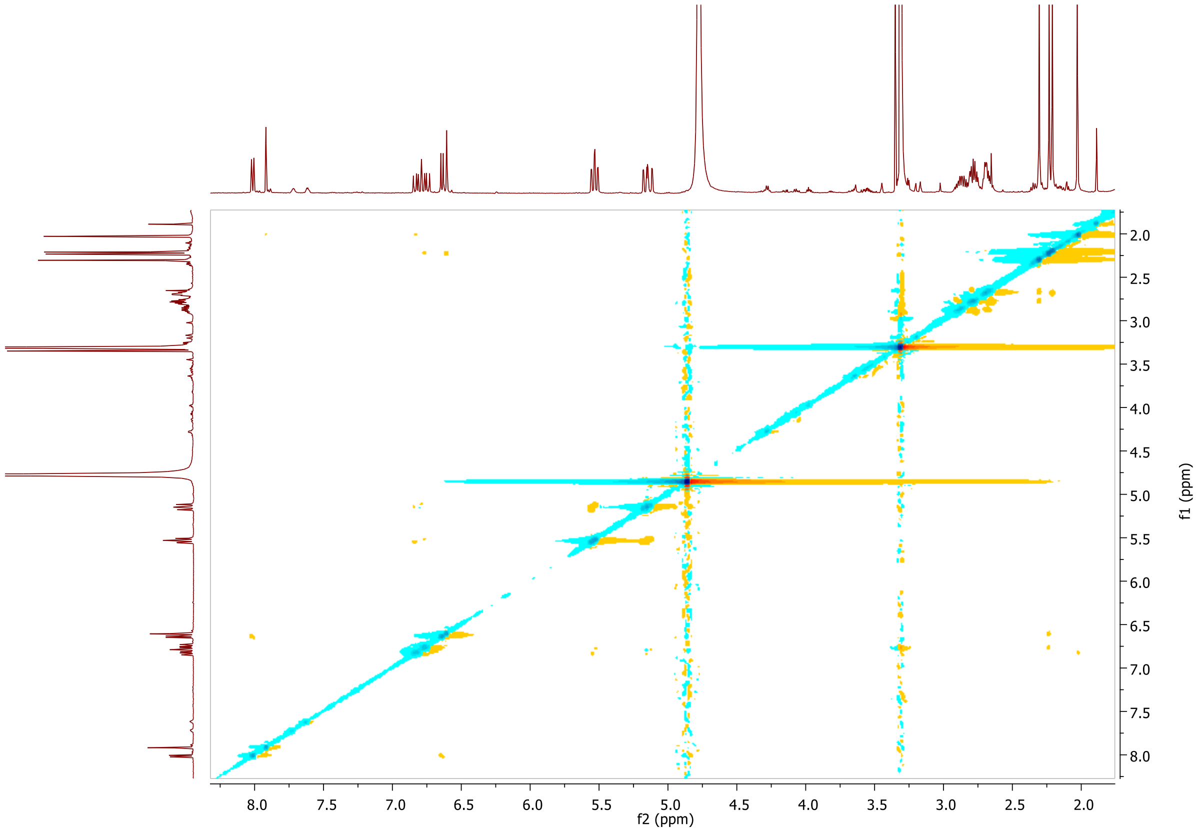

Figure S72. NOESY spectrum of compound **12** (in CD_3OD).

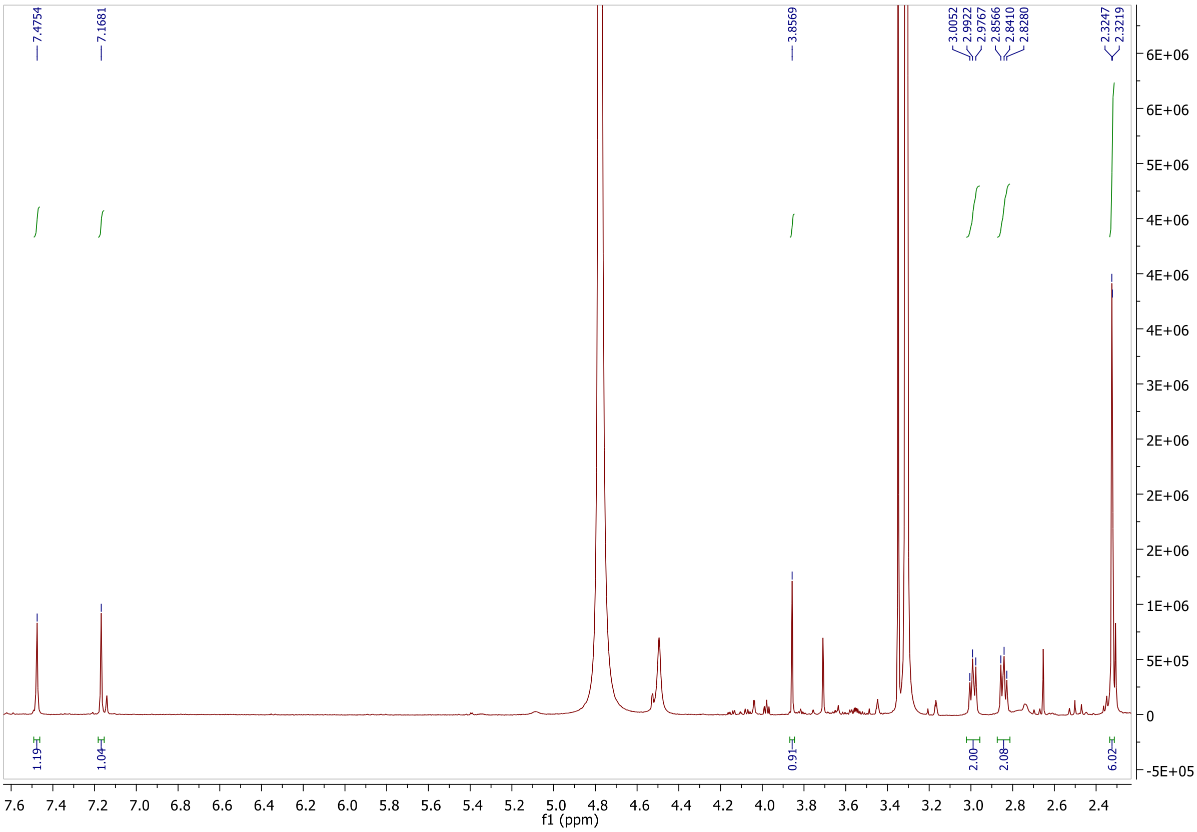

Figure S73. ^{1}H NMR spectrum of compound **13** (in CD_3OD).

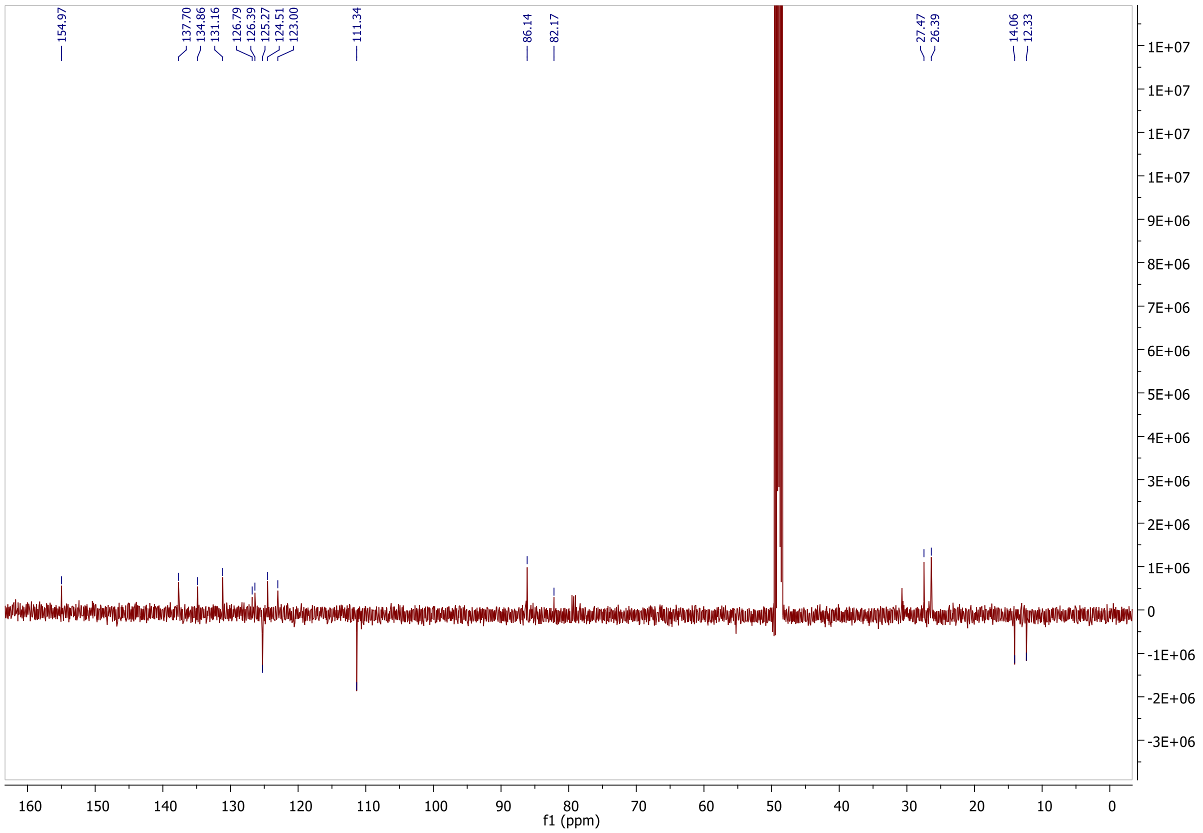

Figure S74. ^{13}C (JMOD) NMR spectrum of compound **13** (in CD_3OD).

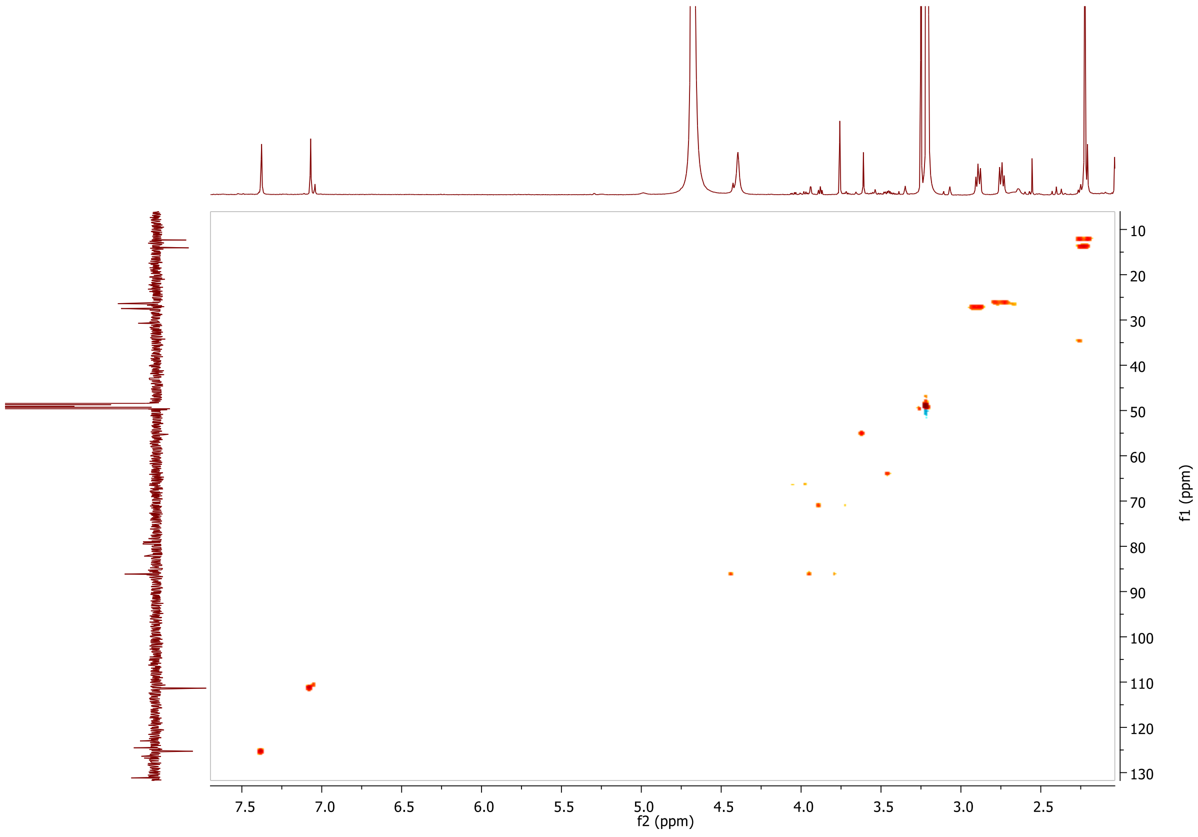

Figure S75. HSQC spectrum of compound **13** (in CD_3OD).

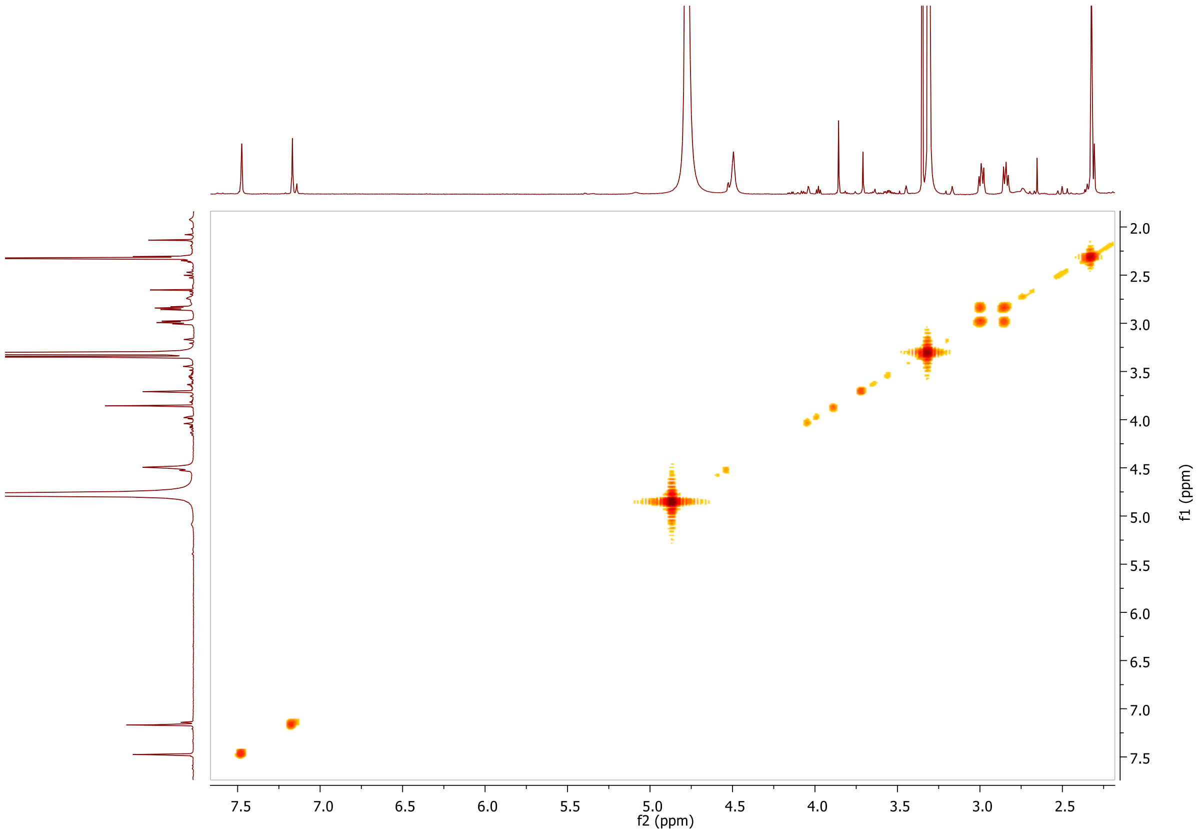

Figure S76. ^1H-^1H COSY spectrum of compound **13** (in CD_3OD).

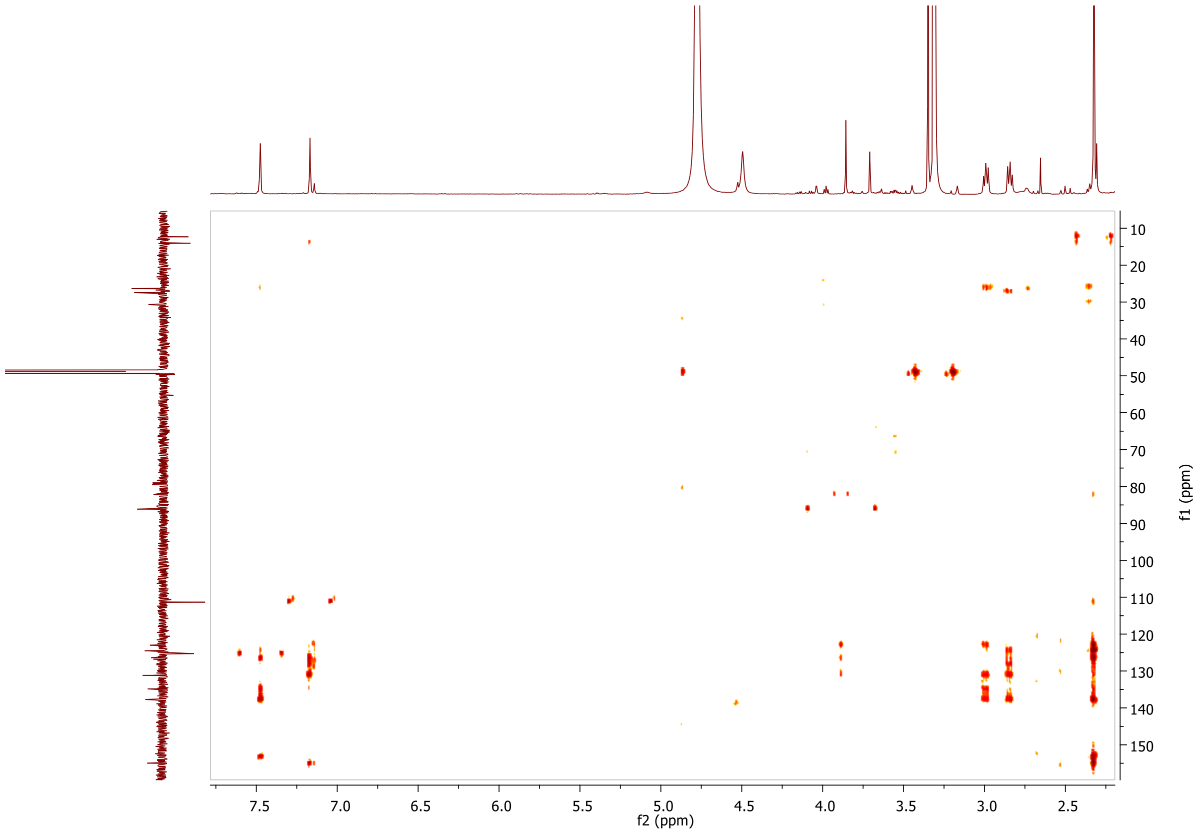

Figure S77. HMBC spectrum of compound **13** (in CD_3OD).

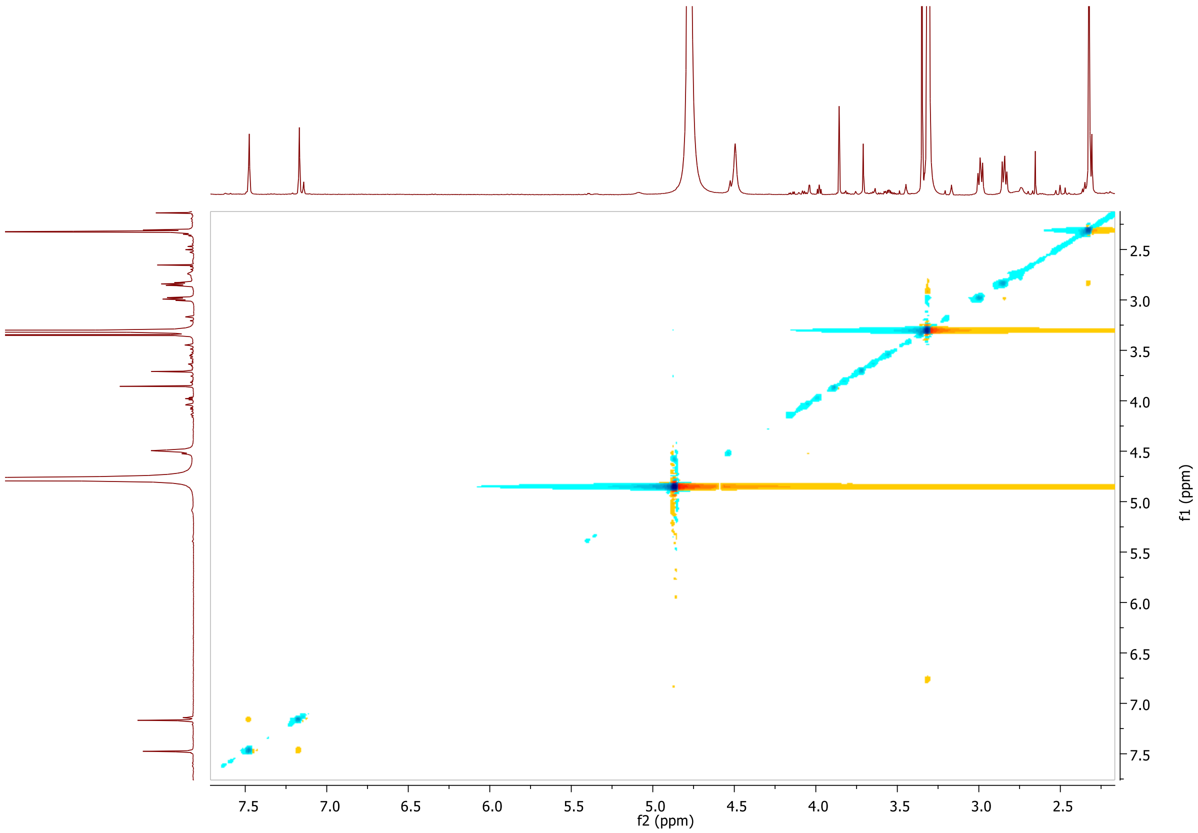

Figure S78. NOESY spectrum of compound **13** (in CD_3OD).
